# Dissecting Response to Cancer Immunotherapy by Applying Bayesian Network Analysis to Flow Cytometry Data

**DOI:** 10.3390/ijms22052316

**Published:** 2021-02-26

**Authors:** Andrei S. Rodin, Grigoriy Gogoshin, Seth Hilliard, Lei Wang, Colt Egelston, Russell C. Rockne, Joseph Chao, Peter P. Lee

**Affiliations:** 1 City of Hope National Medical Center, Department of Computational and Quantitative Medicine, Beckman Research Institute, 1500 East Duarte Road, Duarte, CA 91010, USA; ggogoshin@coh.org (G.G.); shilliard@coh.org (S.H.); rrockne@coh.org (R.C.R.); 2 City of Hope National Medical Center, Department of Immuno-Oncology, Beckman Research Institute, 1500 East Duarte Road, Duarte, CA 91010, USA; lwang@coh.org (L.W.); cegelston@coh.org (C.E.); 3 City of Hope National Medical Center, Department of Medical Oncology & Therapeutics Research, 1500 East Duarte Road, Duarte, CA 91010, USA; jchao@coh.org

**Keywords:** Bayesian networks, machine learning, flow cytometry, immuno-oncology, FACS, immune networks, gating

## Abstract

Cancer immunotherapy, specifically immune checkpoint blockade, has been found to be effective in the treatment of metastatic cancers. However, only a subset of patients achieve clinical responses. Elucidating pretreatment biomarkers predictive of sustained clinical response is a major research priority. Another research priority is evaluating changes in the immune system before and after treatment in responders vs. nonresponders. Our group has been studying immune networks as an accurate reflection of the global immune state. Flow cytometry (FACS, fluorescence-activated cell sorting) data characterizing immune cell panels in peripheral blood mononuclear cells (PBMC) from gastroesophageal adenocarcinoma (GEA) patients were used to analyze changes in immune networks in this setting. Here, we describe a novel computational pipeline to perform secondary analyses of FACS data using systems biology/machine learning techniques and concepts. The pipeline is centered around comparative Bayesian network analyses of immune networks and is capable of detecting strong signals that conventional methods (such as FlowJo manual gating) might miss. Future studies are planned to validate and follow up the immune biomarkers (and combinations/interactions thereof) associated with clinical responses identified with this computational pipeline.

## 1. Introduction

The automated analysis of high-dimensional cytometry data is an active bioinformatics research area [1,2]. Increasingly more sophisticated approaches, mostly of the data mining/machine learning variety, have recently been proposed for the primary analysis of such data [3,4,5,6,7]. However, the emphasis remains on primary data analysis, which is largely reduced to automated gating, clustering, visualization and, subsequently and optionally, cellular identity/population type assignment [8,9,10]. The identification of clinically useful markers (and, perhaps even more importantly, marker combinations) is a secondary data analysis task, belonging to the realm of computational systems biology. To the best of our knowledge, there is currently no systems biology data analysis pipeline aimed specifically at high-dimensional data (including cytometry) in the immuno-oncology domain [11,12,13,14]. For example, recent works in the field [15,16,17,18,19] either reduce the markers’ deduction to classification and semimanual/pairwise combinatorics, or rely on ontological protein–protein interaction networks. Although these analyses are elegant and valid, their completeness and generalizability are uncertain. Notably, such analyses are deficient in automatically inferring higher-order interactions from the data. This is where network-based approaches, such as Bayesian network (BN) modeling, may be useful. 

Only one flow cytometry dataset (RAF signaling pathway) has been subjected to the BN treatment to-date [20,21,22,23,24]. While this pioneering and important work was valuable as a proof-of-concept [20,21], and as a useful platform for further BN methodology development [22,23,24], it did not lead to wider acceptance of the BN methodology in the field of computational flow cytometry. In this study, we introduce a comprehensive BN-centered analysis strategy aimed at FACS (fluorescence-activated cell sorting) data analysis in the immuno-oncology context. As a part of this strategy, we introduce a novel technique, based on the “indicator/contrast variable”, for comparing similar but distinct BNs (such as generated in responders vs. nonresponders, before vs. after treatment, etc.).

Here we show an application of BN analysis to gastroesophageal adenocarcinomas (GEA) immuno-oncology data. GEA are comprised of both stomach and esophageal cancers in aggregate, accounting for a large proportion of global cancer-related mortality [25]. In addition to surgery, chemotherapy, radiotherapy, and targeted therapy, immunotherapies such as immune checkpoint blockade (ICB) can serve as promising treatment options for GEA patients. However, the current response rate to ICB in GEA patients is low, with objective response rates of less than 20% in all-comers [26]. Therefore, it is critical to identify biomarkers that can effectively predict the clinical response to ICB in a subset of GEA patients. 

Currently, reliable predictive ICB biomarkers for GEA outside of genomic microsatellite instability (MSI) and Epstein–Barr virus (EBV) detection remain very limited; such potential predictive biomarkers can be identified based on the systemic immunological features that can be measured by using peripheral blood immune cells from GEA patients. 

In our study, we analyzed peripheral blood mononuclear cell (PBMC) samples from metastatic GEA patients receiving anti-PD-1 (Pembrolizumab) along with radiation therapy. Three immunology flow cytometry panels (Adaptive, Checkpoint, Innate) were generated from each sample (see Section 4 Materials and Methods: Flow Cytometry for detailed panel descriptions) at baseline before the therapy (day 1) and after therapy (day 21). As a result, immunological changes in PBMC samples could be correlated to clinical response to immunotherapy in these patients. 

The results of this study are summarized in the three sections below. The first section details the application of BN analysis to the three immune flow cytometry panel datasets in all patients at day 1. We further scrutinize the resulting BNs to identify biomarkers (and combinations thereof) predictive for immunotherapy response. In the second section, we perform more granular analyses, separating the data into four different immune cell types and contrasting the resulting networks between responders and nonresponders, at day 1 and day 21. In the third section, we discuss the BN validation using statistical criteria, and compare BN modeling with other conventional multivariate analysis methods. 

A somewhat unexpected result, described in the third section, was the clear dichotomy between the two groups of analysis methods—(i) more conventional (in the immuno-oncology context) FlowJo, regression and correlation approaches vs. (ii) BNs, multivariate classifiers and distribution distance metrics. In one striking example, the latter methods uniformly pinpointed a strong predictive biomarker that conventional methods failed to detect. Therefore, one of the conclusions of this study is that the investigators should augment conventional flow cytometry data analysis methodology with these more sophisticated analysis tools.

## 2. Results

### 2.1. Basic Computational Pipeline, and Baseline Immune FACS Panels Analyses

Our basic computational pipeline consists of: (1) discretizing FACS measurements (compensated fluorescence intensity values, obtained directly from FlowJo FACS software [27] via exported FCS files) into 2–8 equal-frequency bins; (2) performing full BN analysis (i.e., reconstructing the complete BN) for one FACS panel, clinical response status variable, and other available variables using our BN modeling software (see Section 4 Materials and Methods: Bayesian networks modeling for details); (3) isolating a Markov blanket/neighborhood (MN) of the response status variable; (4) analyzing the interplay of (usually no more than 4–8) immune system-related markers within the MN; (5) generating a compact set of rules for predicting response status from the markers’ values; and (6) associating these rules with immune cell population subtypes. Afterwards, the results obtained at different immunotherapy timepoints can be compared and contrasted (which are described in the next section, “contrast” analyses). 

Three types of immune FACS panels were analyzed, Checkpoint, Innate and Adaptive, with 14 immune markers in each (see Section 4 Materials and Methods: Flow cytometry for details). We will first illustrate the pipeline on the example of Checkpoint FACS panel in GEA patients. The data contained 14 relevant PBMC immune marker variables measured (at day 1, before therapy, and day 21, after therapy) in 13 patients undergoing immunotherapy. Four patients exhibited significant (<−25% radius) tumor shrinkage and were labeled responders, with remaining nine labeled nonresponders. All 13 patients were pooled together into a single dataset, and the complete dataset was used for three-bin (“low”, “medium” and “high”) equal-frequency discretization of 14 marker variables.

**Figure 1:**
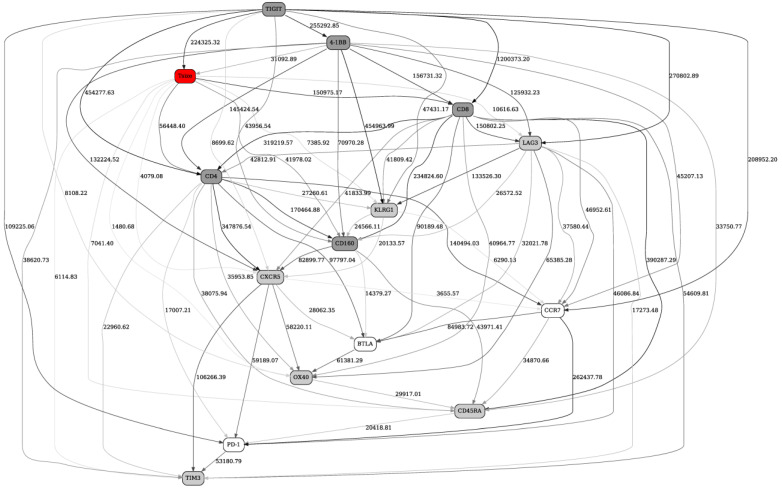
A full Bayesian network (BN) constructed from fluorescence-activated cell sorting (FACS) data (Checkpoint panel) from 13 patients preimmunotherapy treatment (day 1). The number next to the edge and edge thickness indicate dependency strength; arrows/edge directionalities distinguish parent/offspring relationships during the BN reconstruction, and do not necessarily imply causation flow (see Section 4 Materials and Methods: Bayesian networks modeling for details). “Tsize” node, highlighted in red, is the response status variable (4 responders, 9 nonresponders). Remaining nodes are immune marker variables. Response status node is directly strongly linked with five markers (TIGIT, CD4, CD8, CD160, 4-1BB, designated by dark grey nodes in the network) and, less strongly, with six other markers (TIM3, CD45RA, OX40, CXCR5, KLRG1, LAG3, light grey nodes in the network). See text for further details.

Figure 1 depicts the full reconstructed Checkpoint BN at day 1 (pretreatment). The response status variable is designated “Tsize”, for “absolute tumor size (radius) change”, and is shown as the red node in the graph. It is clear from the network that response status is directly strongly linked with five markers (TIGIT, CD4, CD8, CD160, 4-1BB, designated by dark grey nodes in the network) and, less strongly, with six other markers (TIM3, CD45RA, OX40, CXCR5, KLRG1, LAG3, light grey nodes in the network). Because the edges connecting Tsize to the latter six are, on average, significantly weaker than the edges between the latter six and the former five, we will only consider the former five markers as potential predictors of response to therapy. For example, although there is a direct edge between Tsize and CXCR5, it is very weak compared to the Tsize–CD4 and CD4–CXCR5 edges, and so we conclude that the Tsize–CXCR5 edge is likely an artifact of the very strong CD4–CXCR5 dependence. 

Recall that the five variables directly influencing response status (TIGIT, CD4, CD8, CD160, 4-1BB) were discretized into three bins, “low”, “medium”, and “high”. Therefore, there are 3^5 = 243 possible configurations for these five variables. This is much better than the 3^14 ~= 4.8 million configurations in the full dataset before BN-derived dimensionality reduction, but still not feasible for the configuration-by-configuration manual scrutiny. However, we can rank these 243 configurations in order of decreasing frequency in the dataset (which indirectly map to the immune cell population types, from most to least frequent), and select a small number of most frequent configurations (using prespecified, or frequency distribution shape-driven, cutoffs). Then, we can re-sort them in order of decreasing probability of clinical response (tumor shrinkage) given the configuration. We illustrate this approach in Table 1 by showing the 15 most frequent configurations in order of decreasing probability of response—meaning that responsive patients are associated with higher than randomly expected frequency of top-ranking configurations; these configurations might roughly correspond to particular immune cell population types. (The actual number of configurations, 15, was suggested by the shape of the configuration frequency function, with a noticeable dropoff (~0.02 to ~0.017) between the 15th and 16th configurations; the next noticeable dropoff is between the 35th and 36th configurations, and we have chosen the 15 configurations cutoff for presentation compactness).

As summarized in Table 1, the analysis results are now compact enough to allow “manual” scrutiny. Note that the above process is straightforward and largely objective (in that it does not rely on, for example, subjective manual gating decisions)—the only two adjustable parameters are (i) the number of the potentially predictive variables to be selected from the MN of the response status variable, and (ii) the number of the most frequent marker configurations. In this example, the data itself suggested 5 and 15, respectively.

The above strategy (sorting first by frequency, then by response probability) favors the signals associated with more frequent marker combinations. Alternatively, one can sort by response probability only. Marker combinations associated with very high (or very low) response probabilities are likely to be less frequent. Such a strategy is very similar to rare cells subsets discovery algorithms. In general, it is up to the investigator how the conditional probability tables are to be re-sorted once the list of potentially predictive variables is reduced to these inside the MN of response variable. The advantages of the “frequency-then-probability” sorting, as exemplified above, are two-fold: first, higher statistical robustness of discovered signals; second, a (typically) compact set of “rules” describing these signals.

By considering simultaneously the MN of the response variable (Figure 1), and the list of the most frequent marker configurations (Table 1), it is possible to elucidate a set of broad predictive patterns with respect to the clinical response variable. One such pattern, clearly discernible in the marker configuration list (Table 1), is a demarcation line between the top nine and the bottom six configurations. The former correspond to the higher response probability and show, on average, high marker concentrations (more “2”s across the board). The latter correspond to the low response probability and show lower marker concentrations (more “0”s across the board). In addition, Figure 1 suggests TIGIT (and, to a lesser extent, CD8) as the strongest individual predictor(s) of the clinical response. Indeed, when discretized into two bins, high TIGIT values correspond to 0.28572 response probability, while low TIGIT values—0.051155 response probability. 

**Figure 2:**
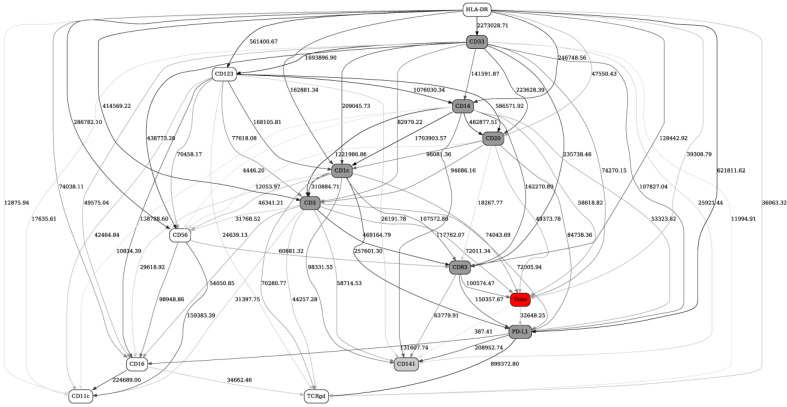
A full BN constructed from FACS data (innate panel) from 13 patients preimmunotherapy treatment (day 1). See Figure 1 legend for further details.

We will now consider the Innate and Adaptive panels. First, Figure 2 depicts the BN generated from the same 13 patients using Innate FACS panel. Out of 14 relevant immune markers, response status (red “Tsize” node in the graph) is directly strongly linked with eight markers (HLA-DR, PD-L1, CD3, CD20, CD83, CD1c, CD14, CD33, dark grey nodes in the network) and, less strongly, with one other marker (CD141, light grey node in the network).

An 8-marker configuration frequency table is shown in Table 2. The actual number of configurations shown (20) was suggested by the shape of the configuration frequency function, which had the first noticeable dropoff at 20. 

Similar to Table 1 (Checkpoint FACS panel), the most striking feature of the marker configuration list (Table 2) is a clear demarcation line between the top 13 and the bottom 7 configurations. The former correspond to the higher response probability and show, on average, high marker concentrations (more “2”s across the board). The latter correspond to the low response probability and show lower marker concentrations (more “0”s across the board). However, in contrast to the Checkpoint network results, no particular markers (out of 8) stand out as the strongest individual predictors. In conclusion, on average, high values of HLA-DR, PD-L1, CD3, CD20, CD83, CD1c, CD14 and CD33 predict favorable response to immunotherapy. 

**Figure 3:**
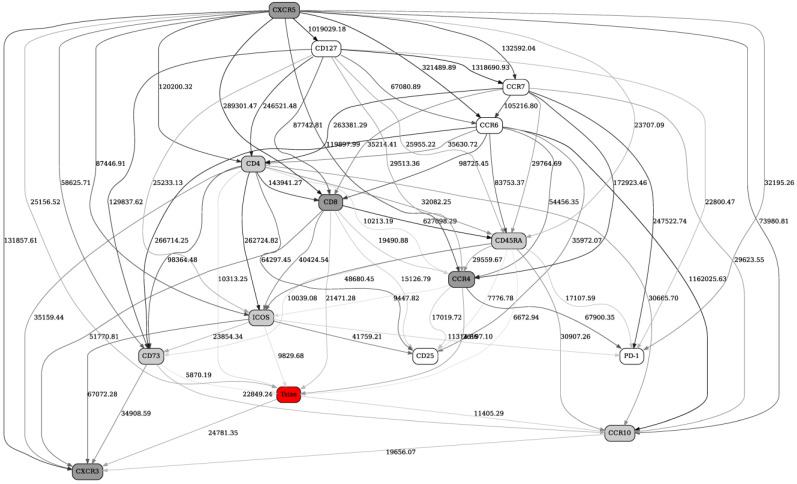
A full BN constructed from FACS data (Adaptive panel) from 13 patients preimmunotherapy treatment (day 1). See Figure 1 legend for further details.

Finally, Figure 3 depicts the BN generated from the same 13 patients using Adaptive FACS panel. Out of 14 immune markers, response status (red “Tsize” node in the graph) is directly strongly linked with four markers (CXCR3, CCR4, CD8, CXCR5, dark grey nodes in the network) and, less strongly, with five other markers (CCR10, ICOS, CD73, CD4, CD45RA, light grey nodes in the network).

A 4-marker configuration frequency table is shown in Table 3. The actual number of configurations shown (10) was suggested by the shape of the configuration frequency function, which had the first noticeable dropoff at 10. Similar to the Table 1 and Table 2, the top rows (higher probability of response) show, on average, high marker concentrations (more “2”s across the board), and the bottom rows (low probability of response) show lower marker concentrations (more “0”s across the board). Notably, the fifth row presents an almost perfect “borderline” case—medium concentrations for all markers (all “1”s), and an intermediate probability of response (0.08891941). No particular markers (out of 4) stand out as the strongest individual predictors. In conclusion, on average, high values of CXCR3, CCR4, CD8 and CXCR5 predict a favorable response to immunotherapy.

### 2.2. Separation into Major Immune Cell Types, and “Contrast” (before/after Immunotherapy, Responders/Nonresponders) Analyses in Checkpoint and Adaptive Networks

In the above analyses, we have used all markers available on the corresponding FACS panels, with all immune cell types and subtypes pooled together. To achieve a higher analysis granularity, we have further separated each FACS dataset into four subsets, according to the four major immune cell types: (1) Naïve CD4+ T cells, (2) Naïve CD8+ T cells, (3) Non-naïve CD4+ T cells, (4) Non-naïve CD8+ T cells. These were stratified using CD4, CD8, CCR7 and CD45RA markers, according to the following membership rules: (1) 1011, (2) 0111, (3) 1010 + 1001 + 1000, (4) 0110 + 0101 + 0100, where “1” means high, and “0”—low marker concentration of CD4, CD8, CCR7 and CD45RA, in that order. The cutoff points between “high” and “low” values of compensated immunofluorescence intensity were defined separately for the Checkpoint and Adaptive networks (due to different calibrations) and were as follows: Checkpoint: CD4 = 1500, CD8 = 750, CCR7 = 150, CD45RA = 400; Adaptive: CD4 = 1000, CD8 = 1000, CCR7 = 150, CD45RA = 1000. (We excluded the Innate datasets from these analyses as we did not necessarily expect too many biologically meaningful changes in the Innate networks in the context of a checkpoint blockade trial; however, we plan to analyze the Innate datasets in our future studies).

**Figure 4:**
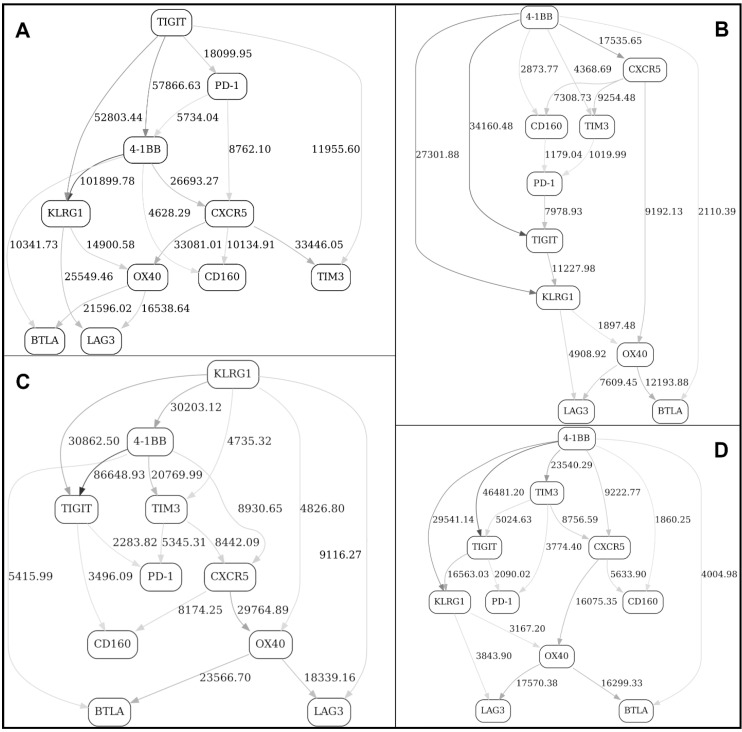
BNs constructed from FACS data (Checkpoint panel, Non-naïve CD4+ T cells subdataset) from 13 patients. (**A**) day 1, nonresponders only, (**B**) day 1, responders, (**C**) day 21, nonresponders, (**D**) day 21, responders. See Figure 1 legend for further details.

Subsequently, we have generated BNs for 32 subdatasets: (responders/nonresponders) x (before/after immunotherapy) x (Checkpoint/Adaptive) x (Naïve CD4+ T cells/Naïve CD8+ T cells/Non-naïve CD4+ T cells/Non-naïve CD8+ T cells). Markers CD4, CD8, CCR7 and CD45RA were naturally excluded from these networks. We used 8-bin discretization throughout to increase specificity (decrease edge density in the networks). The comparisons of the resulting networks allowed us to evaluate changes in immune networks in responders and nonresponders before (day 1) and after (day 21) immunotherapy, stratified by a major immune cell type. In order to quantify and regularize these comparisons, we have introduced “indicator/contrast variables”, as illustrated in the following example (Checkpoint network, Non-naïve CD4+ T cells subdataset): 

Figure 4 depicts four BNs obtained using 8-bin equal-frequency discretization—(A) day 1, nonresponders only, (B) day 1, responders, (C) day 21, nonresponders, (D) day 21, responders.

**Figure 5:**
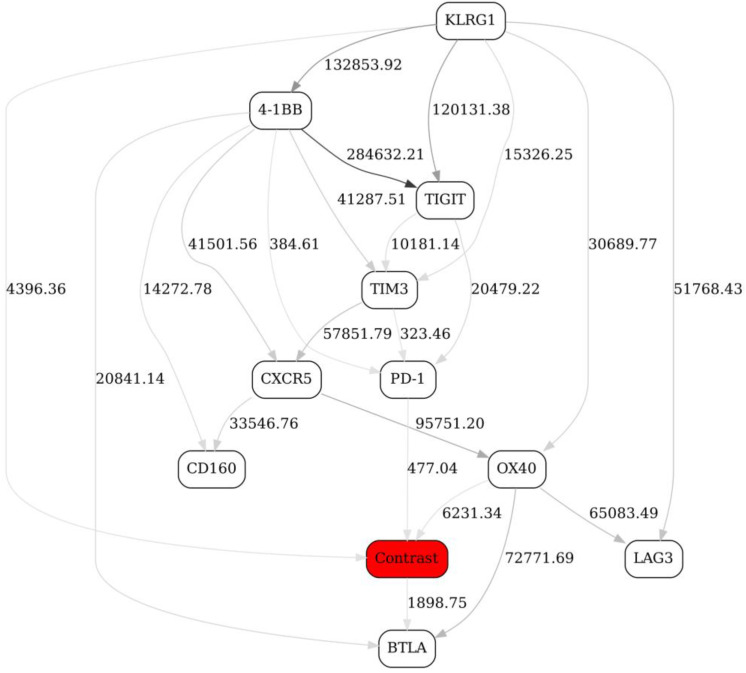
A BN constructed from FACS data (Checkpoint panel, Non-naïve CD4+ T cells subdataset) from 13 patients. The “Contrast” node is the indicator variable with four states, (day 1/nonresponse, day 1/response, day 21/nonresponse and day 21/response). See Figure 1 legend for further details.

At this stage, it is difficult to efficiently compare/contrast these networks in order to tease out the most significant changes, short of manually enumerating the topological differences. Therefore, we have introduced a “Contrast” variable with four distinct states (day 1/nonresponse, day 1/response, day 21/nonresponse and day 21/response) and included it in the pooled, or “supergraph” BN (Figure 5). In Figure 5, four markers (KLRG1, BTLA, OX40 and, to a lesser extent, PD-1) were observed in the MN of the “Contrast” variable (the red node in the graph), suggesting that it is the behavior of these four markers (reflected in the corresponding MNs in the original four stratified networks, Figure 4A–D) that differentiates the responder/nonresponder/day 1/day 21 states.

**Figure 6:**
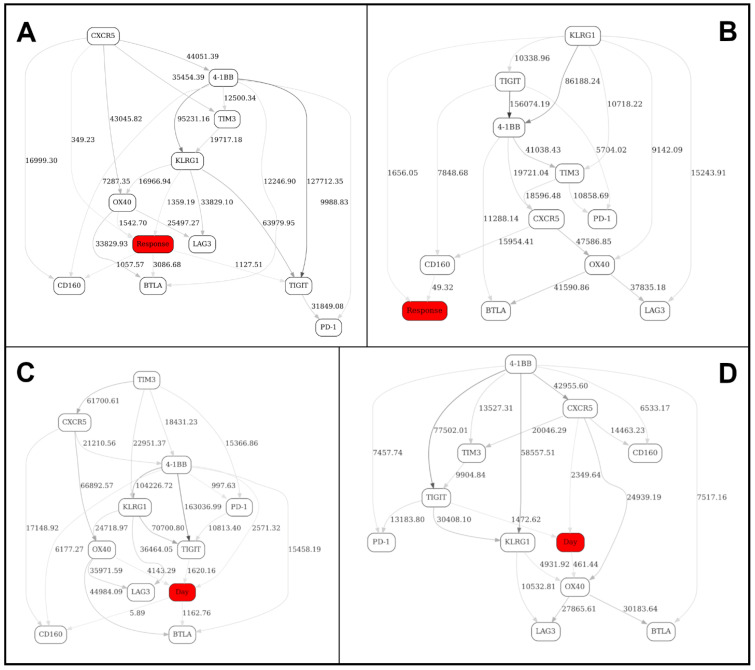
Contrast BNs constructed from FACS data (Checkpoint panel, Non-naïve CD4+ T cells subdataset) from 13 patients. (**A**) at day 1, responders vs. nonresponders, (**B**) at day 21, responders vs. nonresponders, (**C**) nonresponders, at day 1 vs. day 21, and (**D**) responders, at day 1 vs. day 21. “Day” node is the indicator variable with two states (day 1, day 21). “Response” node is the indicator variable with two states (response, nonresponse). See Figure 1 legend for further details.

To look even deeper, we have subsequently introduced two separate contrast variables (“Response” and “Day”, the red nodes in the graph), and constructed four contrast networks shown in Figure 6: (A) at day 1, responders vs. nonresponders; (B) at day 21, responders vs. nonresponders; (C) nonresponders, at day 1 vs. day 21; and (D) responders, at day 1 vs. day 21. 

We were especially interested in Figure 6B, which compares/contrasts responders and nonresponders at day 21 (post-treatment). It appears that there is just one marker with a strong network edge in the MN of the “response” variable, KLRG1. By looking at KLRG1’s MNs in the stratified networks (Figure 4C,D), we conclude that the major difference between the Checkpoint network in responders and nonresponders at day 21 (after immunotherapy treatment) is a relatively less prominent dependency (correlation) between KLRG1 and TIGIT, and an absence of dependency between KLRG1 and TIM3 in responders (compared to nonresponders). This is an example of an interaction, and change thereof, that would have been exceedingly difficult to pinpoint without the BN comparison analysis along the above lines. In other words, our analytical strategy objectively identified the single most important topological difference between the immune networks reflecting two different states (responders vs. nonresponders after immunotherapy). (We need not, of course, concentrate on only the one top difference.)

The results of remaining seven subdataset analyses (that is, Adaptive panel x Non-naïve CD4+ T cells subdataset, and both Adaptive and Checkpoint panels x Naïve CD4+ T cells, Naïve CD8+ T cells and Non-naïve CD8+ T cells subdatasets) are shown in Supplementary File (together with the itemized figure captions/legends, Figures S1–S63). To illustrate another case of a “contrast” network analysis, consider, for example, Figure S52, which contrasts Adaptive Non-naïve CD8+ T cells networks at day 1 between responders and nonresponders. The strongest network edge in the MN of the “response” variable points to ICOS, and after looking at the ICOS node’s MNs in the corresponding stratified networks (Figure S46-day 1, responders, and Figure S47-day 1, nonresponders) we conclude that there are interactions between ICOS and other markers (notably, CD127) that are present in responders but not in nonresponders. Again, just like in a previous example, we were able to objectively single out one strongest interaction (in this case, between ICOS and CD127) that distinguishes between the two different network states.

The above analyses illustrate the process of identifying markers and also higher-order marker interactions that distinguish immune networks in responders and nonresponders before and after immunotherapy treatment. The more theoretical question remains—does the “contrast variable” approach comprehensively capture the topology changes between the networks being compared? In a BN, a presence of an edge is nothing more than a presence of a node in the parent set of another node. Any time an edge is introduced, we are in fact looking at the corresponding change in the joint distribution of the two nodes in question: therefore, the “contrast” node is necessarily dependent on the joint distribution of the two nodes that gain or lose an edge. Consequently, the appearance/disappearance of an edge A->B is reflected in that the contrast C gets “pulled in” as A&B -> C. This is because, in order for contrast C to be independent, we must have P(C | A&B) = P(C). However, P(A&B | C) P(C) = P(A&B) P(C | A&B), and so P(C | A&B) = P(A&B | C) P(C)/P(A&B). Thus, the only way C can be independent of A&B is if P(A&B | C) = P(A&B), which we know is not true because it is given that P(A&B | C=0) ≠ P(A&B | C=1). Therefore, the variations that are not “detected” by the contrast variable must be either noise or structural variants from the same BN equivalence class. Moreover, the appearance/disappearance of an edge is not the only inter-BN difference that will be detected via contrast variables. For example, the same edge could represent a direct or an inverse relationship in two graphs, so one would not necessarily know the difference by just looking at the edges, but it would be a distribution difference that would be captured by a contrast variable all the same. In fact, all the differences associated with going from one graph to another are necessarily nothing but the results of conditioning on the contrast variable in the supergraph—for example, a nonresponse graph G0 (Figure 4C) is simply a supergraph G (Figure 6B) conditioned with contrast C (“Response”) = 0. Therefore, we can expect that all differences will necessarily be encapsulated by the contrast variable connectedness.

### 2.3. Validation of the BNs Using Statistical Criteria, and Comparison of the BN Results with Other Multivariate Analysis Methods

**Figure 7:**
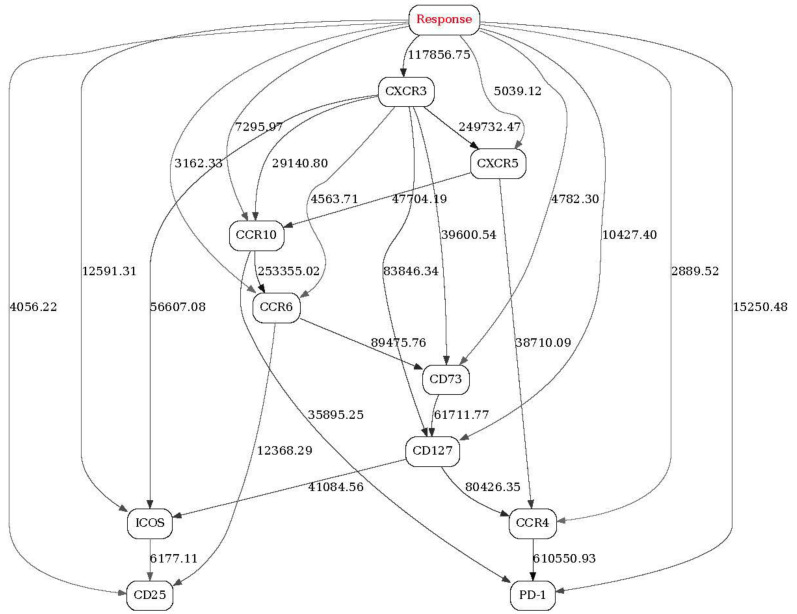
Contrast BN constructed from FACS data (Adaptive panel, Naïve CD4+ T cells) from 13 patients preimmunotherapy treatment (day 1). “Response” (red node in the graph) is the indicator variable with two states (response, nonresponse). See Figure 1 legend for further details.

While the BNs constructed in this study (Figure 1, Figure 2, Figure 3, Figure 4, Figure 5 and Figure 6, Figures S1–S63) are informative in a sense that (i) they suggest the markers most likely to influence the response status and immune network changes and (ii) they can be compared/contrasted among themselves, it is difficult to directly assign the measure of statistical confidence to a particular edge strength in the network. Additionally, it would be instructive to compare the BN analysis with other multivariate analysis methods—namely, multivariate classifiers, and variable selection (importance ranking) methods. In this section, we will illustrate BN validation and comparison with alternative multivariate analysis methods using one particular BN example—responders vs. nonresponders at day 1, Adaptive panel, naïve CD4+ T cells (Figure 7, same as Figure S43). Our interest in this specific subset (naïve CD4+ T cells) was heightened by the observations that, according to the BN analysis, CXCR3 appeared to be a very strong response predictor in naïve CD4+ T cells (Figure 7), and also by the recent literature suggesting an important role of CXCR3 in response to immunotherapy [28,29]. (All standard machine learning and statistical analyses in this section were carried out using the Scikit-learn machine learning toolkit [30], with default parameters unless otherwise noted).

**Figure 8:**
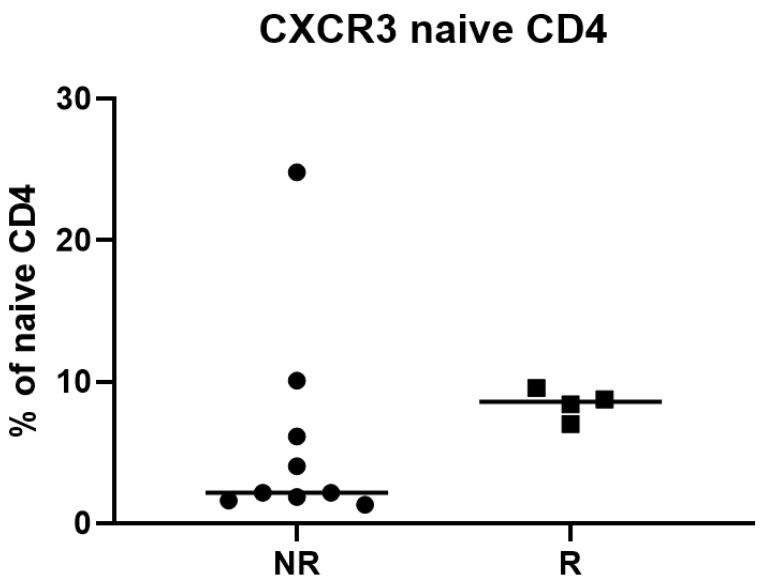
“Traditional” flow cytometry assessment of CXCR3 expression. Naïve CD45RA+ CD4+ CD3+ T cells were manually gated using FlowJo flow cytometry analysis software. The fraction (%) of naïve CD4+ T cells manually judged to express CXCR3 across patient responders (R) and nonresponders (NR) are shown. Unpaired two-tailed t-test resulted in *p*-value of 0.5496, indicating no significant difference between responders and nonresponders.

In order to quantify, in a statistically objective fashion, the association strength between CXCR3 (as well as the other nine markers—see Figure 7) and Response status, we used univariate logistic regression, point-biserial correlation, and distribution distance metrics. The application of the univariate logistic regression and point-biserial correlation (Table 4) did not show significant differences between CXCR3 and other markers in their predictive ability with respect to Response status. Similarly, the results from the FlowJo manual gating analysis [27] showed no indication that CXCR3 was differentially expressed in the naïve CD4+ T cells between responders and nonresponders at day 1 (Figure 8).

**Figure 9:**
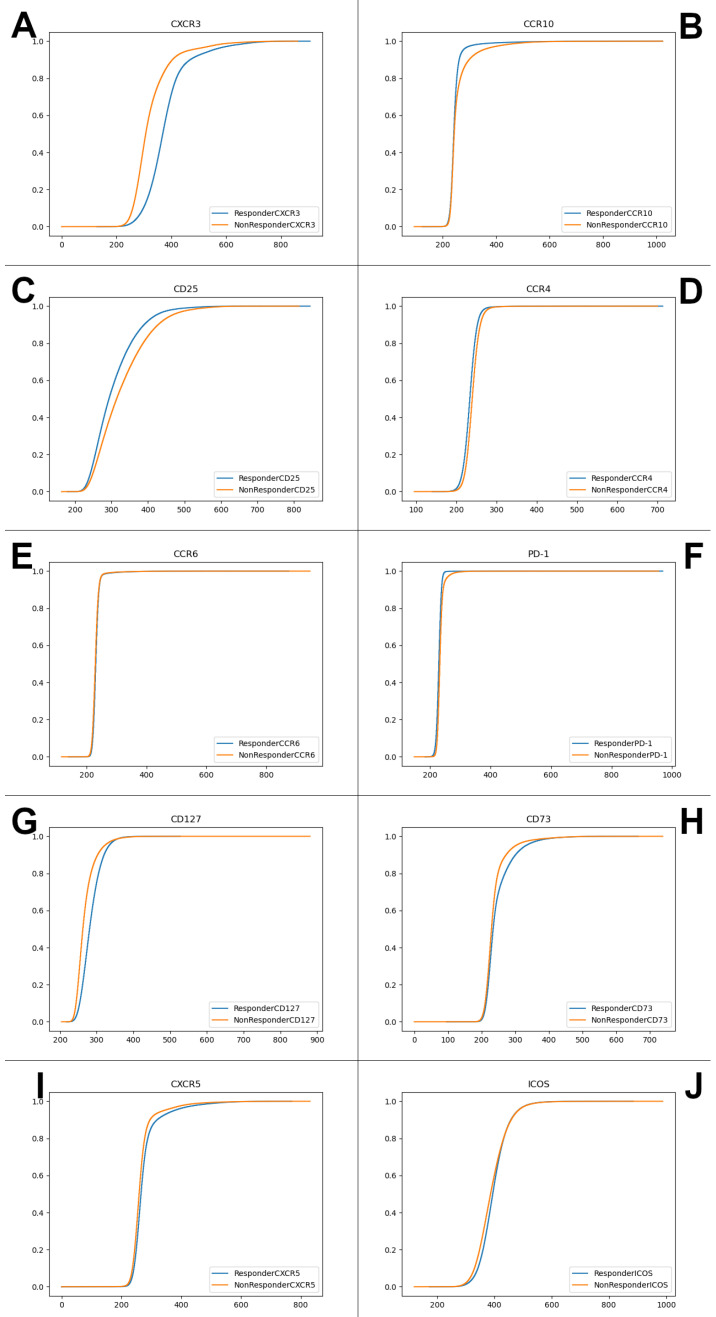
Effective cumulative distribution functions (ECDFs) for CXCR3 and other nine markers’ compensated fluorescent intensities, compared in responders and nonresponders. (Adaptive panel, Naïve CD4+ T cells, day 1). (**A**) CXCR3; (**B**) CCR10; (**C**) CD25; (**D**) CCR4; (**E**) CCR6; (**F**) PD-1; (G) CD127; (**H**) CD73; (**I**) CXCR5; (**J**) ICOS.

**Figure 10:**
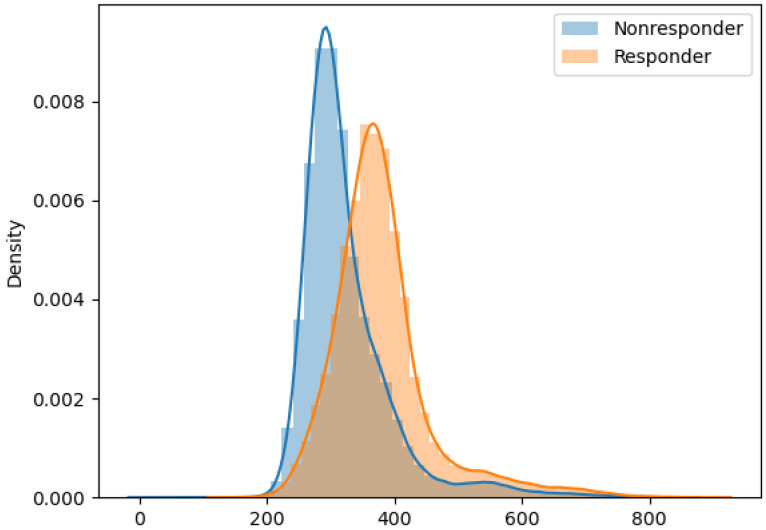
Probability distribution functions (PDFs) for CXCR3 compensated fluorescence intensities, compared in responders and nonresponders. (Adaptive panel, Naïve CD4+ T cells, day 1).

Subsequently, we used Earth Mover’s Distance (EMD) [31] and the Energy Distance (ED) [32] distribution distance metrics to compare differences in effective cumulative distribution functions (ECDFs) of the CXCR3 and the other nine markers’ compensated fluorescence intensity values between responders and nonresponders. Previously, EMD has been shown to be objective and unbiased in comparing biomarker expression levels in cell populations [33]. As can be seen in Figure 9 and Table 5, CXCR3 stands out as by far the strongest discriminator between responders and nonresponders. (To provide a sense of scale, EMD is roughly equal to the area between the two ECDFs—therefore, a difference between, for example, EMD of 55.660 and 22.011, as shown in Table 5, is highly significant; similarly, ED is a linear function of the Cramer Distance, with the same scale sensitivity). Figure 10 further illustrates the significant difference between the probability distribution functions (PDFs) of CXCR3 fluorescence intensity values in responders and nonresponders, in contrast with the “traditional” FlowJo result output (Figure 8).

**Figure 11:**
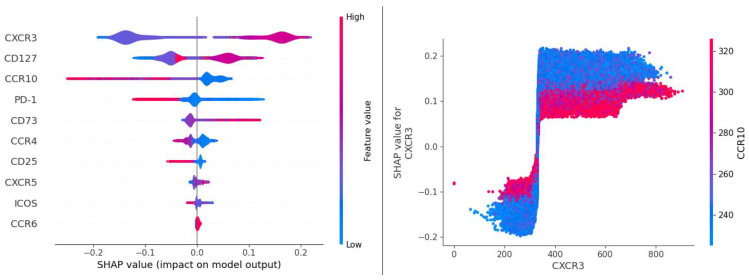
SHAP (Shapley Additive exPlanations) output of the RF model (Adaptive panel, Naïve CD4 cells, responders vs. nonresponders at day 1). The left panel shows a set of “beeswarm” plots reflecting individual variable importance (variables are ranked in descending order) and the impact on the model output (probability of response) depending on the value of the feature (marker). For instance, high values of CXCR3 (red color) act as a positive predictor (right side of the central axis) of response. The right panel shows an example of the interplay between two features, here CXCR3 and CCR10 (low values of CCR10 increase the predictive power of CXCR3). CCR10 was found to be the strongest modulator of CXCR3.

Clearly, there is a conflict. A notable discrepancy between the BN and EMD/ED/distributional results (strong CXCR3 – Response association) on the one hand, and the FlowJo results (no noticeable CXCR3 – Response association) on the other hand can be attributed to the fact that the former methods capture (via multibin discretization with BNs, and full-distribution assessment with EMD/ED) the information that manual gating and simpler statistical analyses simply do not “see”. To further investigate this, we applied select multivariate classifiers to the same data that was used to build the BN in Figure 7. First, we tested various generalized linear models implementing different kinds of regularization and solvers. CXCR3 and, to a lesser extent, CCR10 appeared to be the strongest signals (Table 6). We followed up with the Random Forests (RF) ensemble classifier augmented by the Shapley value-based explainer (TreeSHAP) [34], to look at both individual feature importance and pairwise feature importance. The results (Figure 11) suggested that CXCR3 was indeed the most significant single predictor for the response. Additionally, its significance showed partial dependence on the value of CCR10, the same interplay that was observed in the BN (Figure 7).

To summarize, BN, multivariate logistic regression and RF suggested that the CXCR3 was the strongest predictor for response. This was supported by the distribution distance metrics. On the contrary, the “traditional” FlowJo data analysis workflow did not detect this signal. Finally, the BN and RF analyses also provided an added value of capturing pairwise and higher-level interactions, although the BN modeling was arguably superior in visualization and immediate interpretability (however, recent and ongoing progress in increasing the interpretability of decision-tree-based classifiers [34] might diminish such superiority in time). 

## 3. Discussion

In this study, we developed a novel secondary data analysis strategy aimed at FACS data in the immuno-oncology context. It is based on BN modeling, maximum entropy discretization, multistage marker configuration frequency sorting, and pooling subdataset BNs while introducing “contrast” variables. The end output of our computational pipeline consists of the following components: (a) a compact subset of potentially predictive (with respect to a clinical outcome or some other phenotype) markers, typically in the single digit (no more than 4–8 markers) range; (b) a graphical causal model of the interactions between these predictors and other variables, together with the corresponding relative interaction strengths; (c) a compact list of the most frequent marker combinations, together with the corresponding outcome/phenotype probabilities; and (d) a shortlist of markers whose behavior (as reflected in their corresponding MNs in the stratified subnetworks) is significantly different between the varying immune network states (responders/nonresponders, before/after immunotherapy). While component (b) has been implemented in the flow cytometry context before (RAF signaling pathway, [20,21,22,23,24]), the other parts of our pipeline are novel. Taken together with the statistical validation and intermethod comparison detailed in the preceding section, they make the BN methodology much more appealing and practical in its application to the flow cytometry data.

One advantage of this analytical strategy is that it is straightforward, objective and generalizable to other similar datasets of varying data structure and dimensionality. At the same time, it allows for a certain amount of flexibility in the analysis process. First, different discretization approaches can be tried depending on the investigators’ understanding of what “high” and “low” might mean for a particular marker concentration. Second, more (or fewer) potential predictors can be selected from the outcome/phenotype variable’s MN, depending on the BN’s local sparseness and the general pattern of conditional independencies in the network. (This, however, is a complicated issue, and we plan to investigate it further as part of our ongoing BN methodological research.) Third, more (or fewer) potential predictor configurations (and corresponding cell types) can be scrutinized at later analysis stages, depending, for example, on the size and coverage/purpose of the concrete FACS panel, or on whether investigators are interested in rare cell types that are very strongly associated with particular outcomes/phenotypes. 

There are two additional interrelated methodological caveats that are intrinsic to the BN modeling in general: dependency strength validation, and internetwork comparisons. Throughout this study, we use edge strengths to estimate statistical support for the networks and their features. Edge strength is conceptually similar to the marginal likelihood ratio test, in that it is proportional to the ratio of the BN model scoring function value when the edge is present in the BN to the BN model scoring function value when the edge is absent in the BN. Edge strengths do not directly translate into *p*-values; therefore, once a particular dependency/association is suggested by the BN, and is of interest to the investigators, one should follow up with “traditional” statistical tools. This said, edge strengths are directly comparable to each other within a single BN. However, it is not immediately clear whether they are directly comparable to each other between the BNs, even if the corresponding datasets are similar in their dimensionality (and corresponding samples were generated in the same batch, to minimize batch variation). There are reasons to believe that edge strengths are linear functions of the dataset size (e.g., number of cells in one sample in the FACS context), but this aspect requires further investigation. For now, we would not necessarily advise comparing edge strengths between the networks (e.g., between Figure 1, Figure 2 and Figure 3 in this study) unless the BN-generating datasets are very similar in their size and structure. Instead, when possible, the data should be pooled, and indicator/contrast variables (distinguishing the networks to be compared) introduced (e.g., Figure 5 and Figure 6 in this study).

One somewhat unexpected result of our study was a clear divergence between two groups of analytical approaches—FlowJo and simpler analysis methods vs. BNs and multivariate classifiers. Using one example dataset (Adaptive panel, Naïve CD4+ T cells, responders vs. nonresponders at day 1) we have demonstrated that there exists a strong predictive signal, CXCR3, that is reliably “picked up” by the latter, but not by the former. Since many FACS analyses in current immunology research and recent literature rely on the FlowJo data analysis pipeline, we are concerned that strong signals (such as CXCR3 in our example) might be missed. This could be attributed to the intrinsic limitations of the manual gating process [4,9], including such interrelated factors as (i) subjectivity of the manual gating procedure, (ii) discretization in two bins, (iii) loss of the higher-order information, and (iv) linearity assumptions throughout. Therefore, we suggest that in the future investigators should augment the “standard” FlowJo (or similar manual gating) data analysis pipeline with the direct analysis of the compensated fluorescence intensity data, preferably by a variety of methods, and preferably including at least some nonlinear ones. In that, our conclusions dovetail with other recent recommendations in the field [33].

One of the obvious limitations of this study was the low number of patients (14). However, the primary purpose of the study was to propose and develop a novel analytical framework, and to see if it compares favorably with the existing tools. Indeed, our analysis pipeline has successfully identified a number of potentially strong predictive biomarkers (and their combinations), including one biomarker (CXCR3) that was clearly missed by the more conventional methodology. 

It should also be emphasized that the principal advantage of the BN + MN approach lies in the inherent interpretability of the analysis results. Consequently, the BN + MN approach can be more constructive than the commonly used dimensionality reduction/clustering/visualization techniques, in that (i) it presents the structures (full networks and MNs) that are directly mechanistically interpretable, in contrast to the more vaguely defined similarity “clusters” and “trees”, and (ii) it allows researchers to immediately identify, rank and prioritize the important features within these structures. This is not to diminish the value of such dimensionality reduction methods; rather, the BN + MN approach presents an alternative, and oftentimes more natural and straightforward, path from the “pretty pictures” to the actual candidate markers and their interactions. In general, we agree with the recent literature [35] in that while data visualization and clustering algorithms are helpful for the broad exploration of the flow cytometry data, it is the novel supervised machine learning techniques that hold the most promise for a seamless automatization of the analysis of large flow cytometry datasets. This becomes even more relevant in light of the ongoing advent of increasingly higher-resolution (e.g., 40-color) flow cytometry panels [36]. By its very design, our “BN => MN funnel” approach is easily scalable to hundreds of parameters. Similarly, our approach is seamlessly applicable to other high parameter cytometry methods (mass cytometry, spectral cytometry) and high throughput analyses in the immuno-oncology space in general—such as, for example, CITE-seq, as the dimensionality (typically in the 20–100 range for current applications) is easily handled by our BN software [37].

In conclusion, since only some cancer patients tend to be responsive to immunotherapy such as ICB, the development of reliable predictive biomarkers for treatment response is urgently needed to select patients. While recent findings have shown some promise in tumor tissue-based immunological biomarkers, peripheral blood biomarkers are more accessible and less invasive. In this study, peripheral blood FACS data, combined with our analytical strategy, have been successfully used to suggest such biomarkers, and gain insight into their interactions and dynamics before and after treatment. Presently, we plan to validate and follow up the immune markers (and combinations/interactions thereof) associated with clinical responses that were identified by us in this study, such as CXCR3 (and CXCR3–CCR10 interaction), starting with the manual gating of flow cytometry data and confirmation of marker staining relative to experimental isotype or fluorescence-minus-one controls. In the future, we intend to generalize and apply our analysis tools to other cancer types, treatments, and patient cohorts.

## 4. Materials and Methods

### 4.1. Flow Cytometry

Peripheral blood mononuclear cells (PBMCs) from patients consented to an IRB-approved protocol were isolated from heparinized blood by Ficoll-Paque density centrifugation and cryopreserved in FSB with 10% DMSO. Cryopreserved PBMCs were thawed and stained with antibodies for the following flow cytometry panels:

Checkpoint panel: CD4, CD8, CD45RA, KLGR1, CCR7, CXCR5, 4-1BB, BTLA4, LAG3, OX40, CD160, TIGIT, PD1, TIM3;
Innate panel: CD3, CD14, CD16, CD20, CD33, CD56, CD11c, CD141, CD1c, CD123, CD83, HLA-DR, TCRgδ, PD-L1;
Adaptive panel: CD4, CD8, CCR10, CCR6, CD73, ICOS, CXCR3, CXCR5, CD45RA, CCR4, CCR7, CD25, CD127, PD1.

Flow cytometry was performed using Fortessa Flow Cytometers and flow cytometry data was analyzed using FlowJo software (BD Biosciences) [27]. Antibody dilutions were titrated appropriately for each marker to optimize positive staining relative to negative populations. Voltages were set by BD Cytometer Setup and Tracking (CS&T) Beads and software. All samples were run in a single batch to minimize batch variation.

### 4.2. Bayesian Networks Modeling

BN modeling is a systems biology method that constructs a graphical visualization of a joint multivariate probability distribution of the random variables in the dataset. While recent BN modeling software implementations are highly scalable [37], combining scalability with mixed variable types (e.g., continuous and discrete) is less straightforward; an argument can be made [37,38] that the discretization of continuous variables is a better solution, in practical terms, than imposing mixed distribution models. Therefore, in this study we used full sample equal-frequency discretization, which also allowed us to adjust the under/over-fitting balance (i.e., specificity vs. sensitivity). Typically, discretizing into a smaller number of bins (2–3) increases sensitivity (higher edge density), and discretizing into a larger number of bins (4–8) increases specificity (lower edge density). Our BN implementation [37] is based on a hybrid “sparse candidates” + “search-and-score” algorithm with random restarts until convergence (scaling up to ~ 1mln variables x 1mln datapoints on a midrange workstation). In the resulting network structures, we would typically “zoom in” on the immediate Markov neighborhood (MN) of a specific variable (such as an immune system-related marker, or a patient’s clinical response status), which corresponds to all the nodes (variables) directly linked to a specific variable in question. (MN is a simplified version of the Markov Blanket—the latter takes into account parent–offspring relationship directionality—meaning, for the practical purposes, that “two degrees of separation”, or more, are sometimes needed for fully assessing the conditional independence relationships around a specific variable/node.) The numbers next to the edges and edge “thickness” in the resulting BN figures specify the relative edge strengths (which are marginal likelihood-based). Further details on the BN modeling in general and our implementation thereof can be found in [37,39].

## Figures and Tables

**Table 1:** The 15 most frequent predictive immune marker configurations (Checkpoint FACS panel), in descending order of probability of response. First column shows predictive immune marker configurations obtained from the MN of the response status variable, five markers in total (TIGIT, CD4, CD8, CD160, 4-1BB). The values “0”, “1”, “2” in the first column indicate “low”, “medium” and “high” values after discretization. Second column shows configuration frequency; third column—probability of response.

Immune Marker Configuration (TIGIT, CD4, CD8, CD160, 4-1BB)	Configuration Frequency	Probability of Response
(0 1 2 2 2)	0.0256	0.430
(2 1 2 2 2)	0.0252	0.407
(2 2 1 2 2)	0.0328	0.371
(2 2 2 1 2)	0.0443	0.365
(1 1 2 2 2)	0.0268	0.357
(2 2 2 1 1)	0.0256	0.338
(2 2 2 2 2)	0.0346	0.265
(1 2 2 2 2)	0.0336	0.191
(0 2 2 2 2)	0.0341	0.180
(0 0 1 0 0)	0.0193	0.038
(0 0 0 1 0)	0.0307	0.008
(0 0 0 0 0)	0.0644	0.008
(0 0 0 0 1)	0.0193	0.007
(1 0 0 1 0)	0.0199	0.006
(1 0 0 0 0)	0.0338	0.006

**Table 2:** The 20 most frequent predictive immune marker configurations (Innate FACS panel), in descending order of probability of response. First column shows predictive immune marker configurations obtained from the MN of the response status variable, eight markers in total (HLA-DR, PD-L1, CD3, CD20, CD83, CD1c, CD14, CD33). The values “0”, “1”, “2” in the first column indicate “low”, “medium” and “high” values after discretization. Second column shows configuration frequency; third column—probability of response.

Immune Marker Configuration (HLA-DR, PD-L1, CD3, CD20, CD83, CD1c, CD14, CD33)	Configuration Frequency	Probability of Response
(2 1 0 1 2 1 2 2)	0.0182	0.648
(2 2 2 1 2 2 1 0)	0.0096	0.639
(2 1 1 1 2 1 2 2)	0.0308	0.604
(2 2 2 1 2 2 1 1)	0.0098	0.586
(2 1 1 2 2 1 2 2)	0.0163	0.369
(2 2 2 2 2 2 2 2)	0.0269	0.357
(1 2 2 2 1 2 1 0)	0.0081	0.249
(1 2 2 2 1 2 1 1)	0.0176	0.246
(2 1 2 1 2 1 0 2)	0.0086	0.202
(2 2 2 2 2 1 2 2)	0.0430	0.141
(2 2 2 2 2 1 1 2)	0.0090	0.138
(1 2 2 2 1 2 0 0)	0.0080	0.138
(1 2 2 2 1 2 0 1)	0.0078	0.133
(0 0 0 0 0 0 1 0)	0.0147	0.063
(0 0 0 0 0 0 1 1)	0.0074	0.037
(0 0 0 0 0 0 0 1)	0.0086	0.026
(0 0 0 0 1 0 0 0)	0.0158	0.015
(0 0 0 0 0 0 0 0)	0.0603	0.014
(0 0 0 0 0 0 2 1)	0.0085	0.013
(1 0 0 0 0 0 0 0)	0.0187	0.007

**Table 3:** The 10 most frequent predictive immune marker configurations (Adaptive FACS panel), in descending order of probability of response. First column shows predictive immune marker configurations obtained from the MN of the response status variable, four markers in total (CXCR3, CCR4, CD8, CXCR5). The values “0”, “1”, “2” in the first column indicate “low”, “medium” and “high” values after discretization. Second column shows configuration frequency; third column—probability of response.

Immune Marker Configuration (CXCR3, CCR4, CD8, CXCR5)	Configuration Frequency	Probability of Response
(2 1 2 2)	0.0352	0.406
(2 2 2 2)	0.1520	0.377
(2 2 1 2)	0.0401	0.347
(1 2 2 2)	0.0384	0.226
(1 1 1 1)	0.0404	0.089
(0 0 1 0)	0.0351	0.039
(0 1 0 0)	0.0377	0.018
(0 0 0 1)	0.0412	0.008
(0 0 0 0)	0.1240	0.008
(1 0 0 0)	0.0289	0.005

**Table 4:** Univariate logistic regression and point-biserial correlation results. (Adaptive panel, Naïve CD4+ T cells, responders vs. nonresponders at day 1). Classification accuracy was assessed using 10-fold cross-validation.

Variable	Logistic Regression Classification Accuracy(Responders vs. Nonresponders)	Point-Biserial Correlation with Response Status
CXCR3	68.34%	0.32
CCR10	67.80%	−0.12
CD73	67.50%	0.12
CCR6	67.72%	0.05
CD25	67.82%	−0.15
ICOS	67.80%	0.06
CXCR5	67.67%	0.10
PD-1	69.12%	−0.21
CD127	67.80%	0.26
CCR4	67.96%	−0.18
Response	100.00%	1.00

**Table 5:** EMD and ED measures (computed from the ECDFs shown in Figure 9) between responders and nonresponders for CXCR3 and other nine markers. (Adaptive panel, Naïve CD4+ T cells, day 1).

Feature	Earth Mover’s Distance	Energy Distance
CXCR3	55.660	5.569
CCR10	11.506	1.262
CD73	9.819	1.238
CCR6	1.741	0.435
CD25	22.011	2.028
ICOS	6.788	0.916
CXCR5	10.083	1.169
PD-1	4.999	1.066
CD127	15.686	2.607
CCR4	6.479	1.218

**Table 6:** Application of different variants of generalized linear models (Adaptive panel, Naïve CD4 cells, responders vs. nonresponders at day 1). “lbfgs” stands for limited-memory Broyden–Fletcher–Goldfarb–Shanno algorithm; “liblinear”, coordinate descent algorithm; L1, L1 regularization; L2, L2 regularization. Classification accuracy was assessed using five-fold cross-validation. Extended decimals are shown to indicate that the results were similar but not identical.

Model	Classification Accuracy	Coefficients, in order of: {(’CXCR3’, ’CCR10’, ’CD73’, ’CCR6’, ’CD25’, ’ICOS’, ’CXCR5’, ’PD-1’, ’CD127’, ’CCR4’)} - (Intercept)
L2, lbfgs logistic regression	79.8128%	{(1.3640, −1.0545, 0.3220, 0.1558, −0.2632, −0.2438, −0.6944, −0.5492, 0.4632, −0.2202)} (−1.0805)
L2, liblinear logistic regression	79.8107%	{(1.3641, −1.0547, 0.3220, 0.1558, −0.2632, −0.2438, −0.6943, −0.5492, 0.4633, −0.2202)} (−1.0806)
L1, liblinear logistic regression	79.8102%	{(1.3636, −1.0542, 0.3219, 0.1557, −0.2631, −0.2437, −0.6939, −0.5591, 0.4632, −0.2201)} (−1.0803)

## Data Availability

The principal datasets analyzed in this study have been deposited to Dryad (doi:10.5061/dryad.fxpnvx0ng). All intermediate/auxiliary datasets will be made available by the authors, without undue reservation, to any qualified researcher.

## Supplementary Materials

Supplementary materials can be found at https://www.mdpi.com/1422-0067/22/5/2316/s1. Figures S1–S63 depict BNs obtained from the remaining subdatasets in the present study (i.e., Adaptive immune panel x Non-naïve CD4 subdataset, and both Adaptive and Checkpoint panels x Naïve CD4, Naïve CD8 and Non-naïve CD8 subdatasets). Figures S1–S9—Checkpoint, Naïve CD4; Figures S10–S18—Checkpoint, Non-naïve CD8; Figures S19–S27—Checkpoint, Naïve CD8; Figures S28–S36—Adaptive, Non-naïve CD4; Figures S37–S45—Adaptive, Naïve CD4; Figures S46–S54—Adaptive, Non-naïve CD8; Figures S55–S63 – Adaptive, Naïve CD8. Within each group, the nine networks are as follows: Day 1, responders, Day 1, nonresponders, Day 21, responders, Day 21, nonresponders, Day 1 and day 21, responders, with “day” contrast variable, Day 1 and day 21, nonresponders, with “day” contrast variable, Day 1, responders and nonresponders, with “response” contrast variable, Day 21, responders and nonresponders, with “response” contrast variable, “Supergraph” (all data pooled together, with a four-state “contrast” variable).
