# Supplementary material for: Dissecting Response to Cancer Immunotherapy by Applying Bayesian Network Analysis to Flow Cytometry Data
Source: Int J Mol Sci. 2021 Feb 26;22(5):2316. doi: 10.3390/ijms22052316 (PMC7956201; doi:10.3390/ijms22052316)
Supplement: Supplementary file 1 [file ijms-22-02316-s001.pdf]

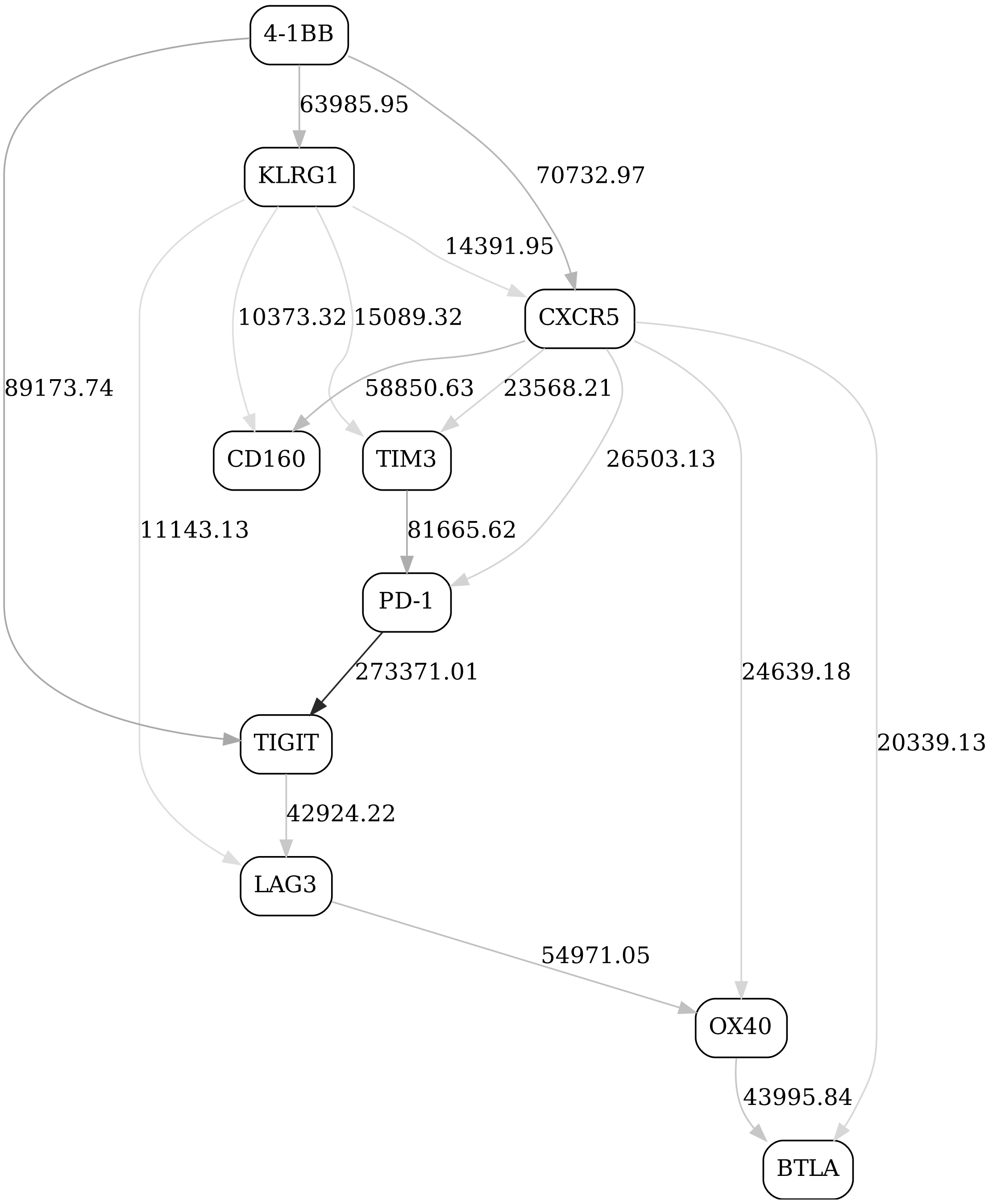

Figure 1: Checkpoint immune signaling network panel, naive CD4, day 1, responders

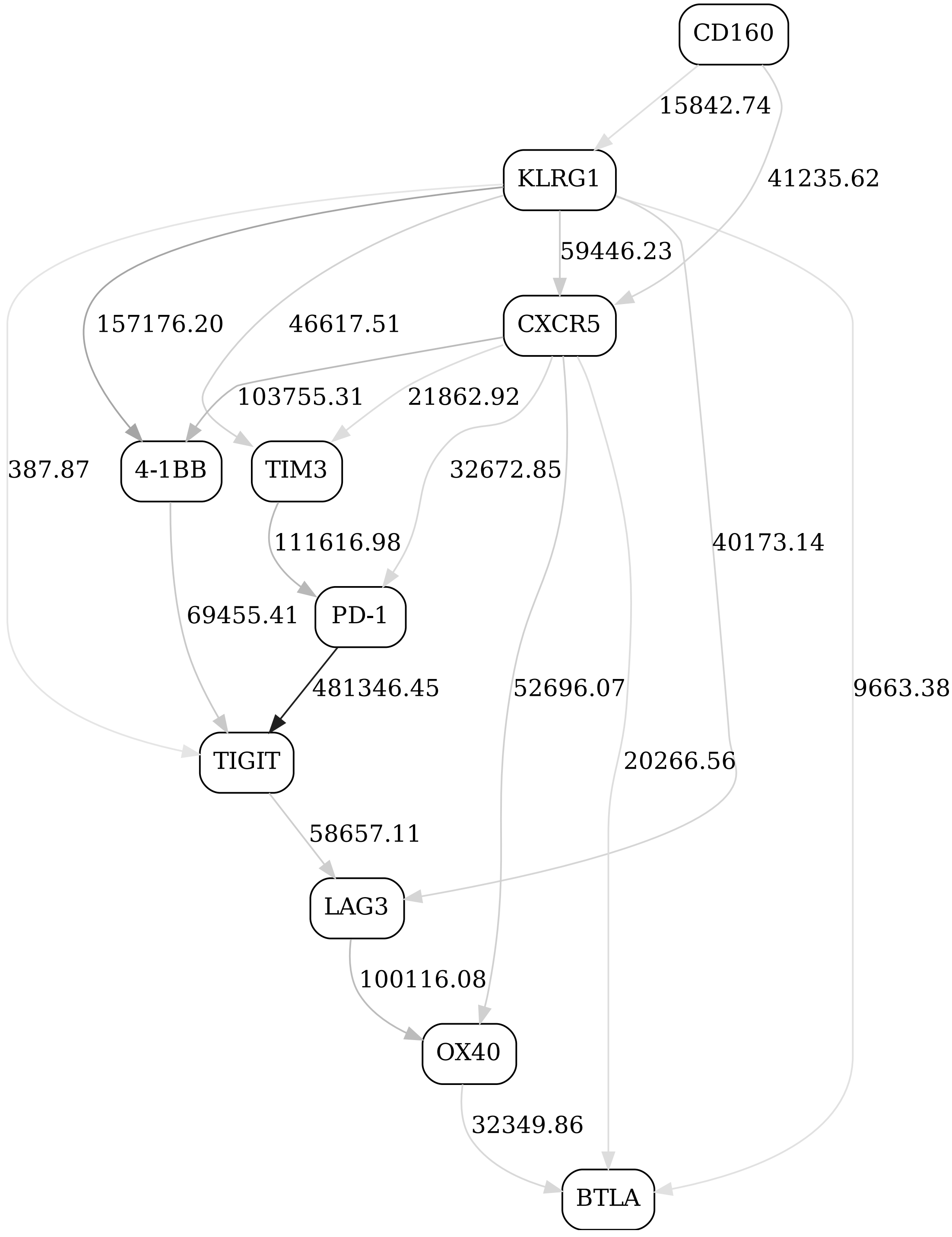

Figure 2: Checkpoint immune signaling network panel, naive CD4, day 1, non-responders

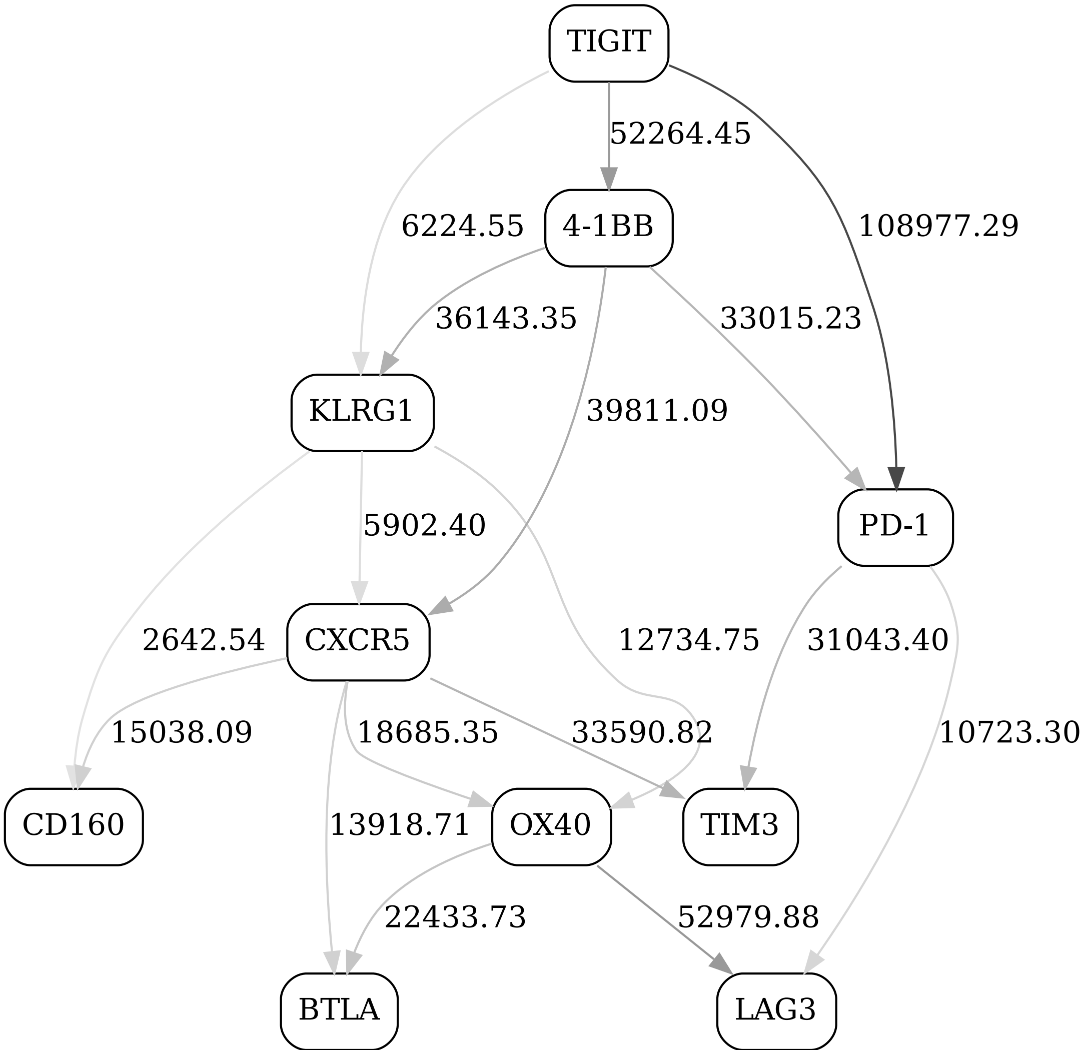

Figure 3: Checkpoint immune signaling network panel, naive CD4, day 21, responders

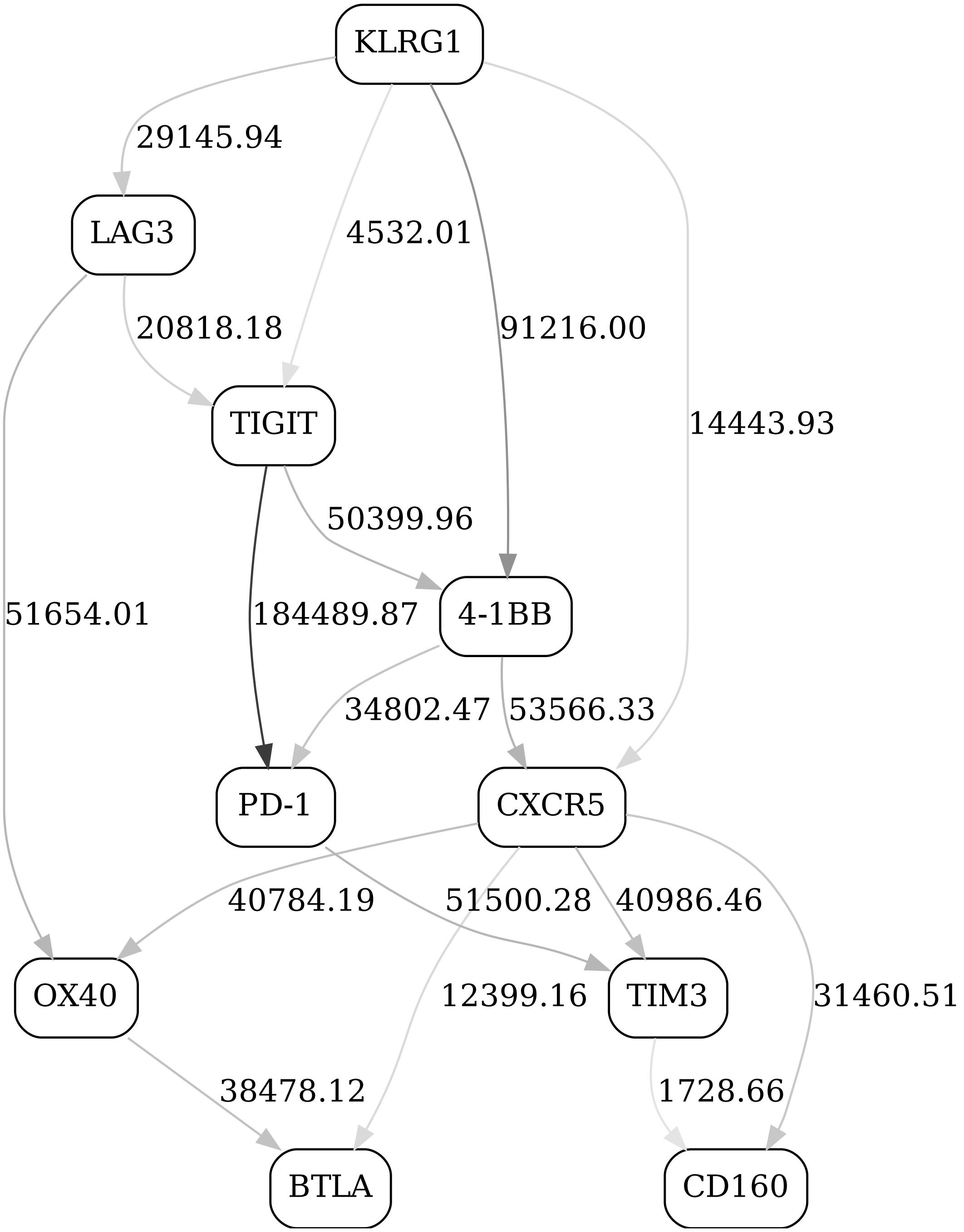

Figure 4: Checkpoint immune signaling network panel, naive CD4, day 21, non-responders

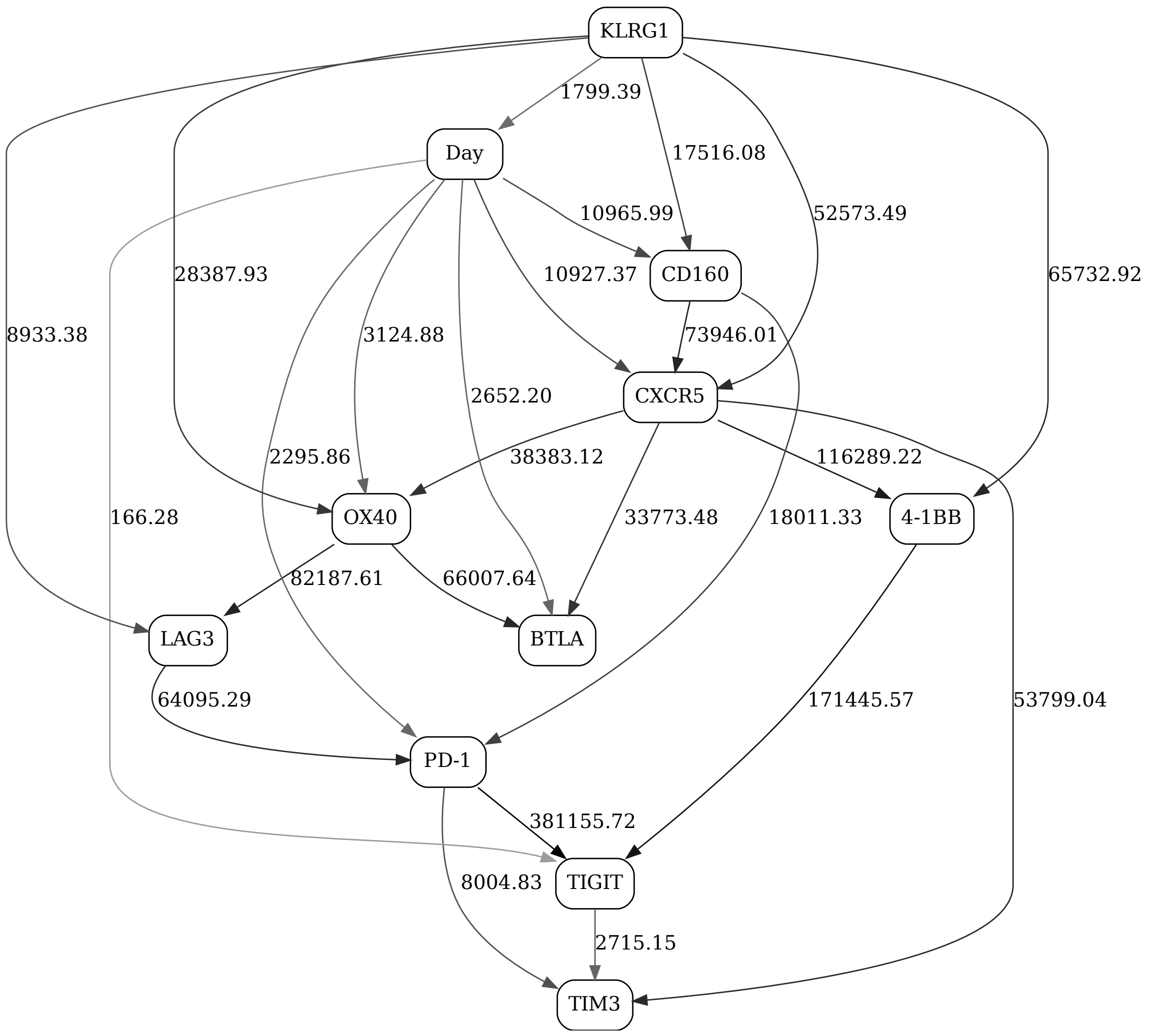

Figure 5: Checkpoint immune signaling network panel, naive CD4, day contrast (2-state "Day" variable), responders

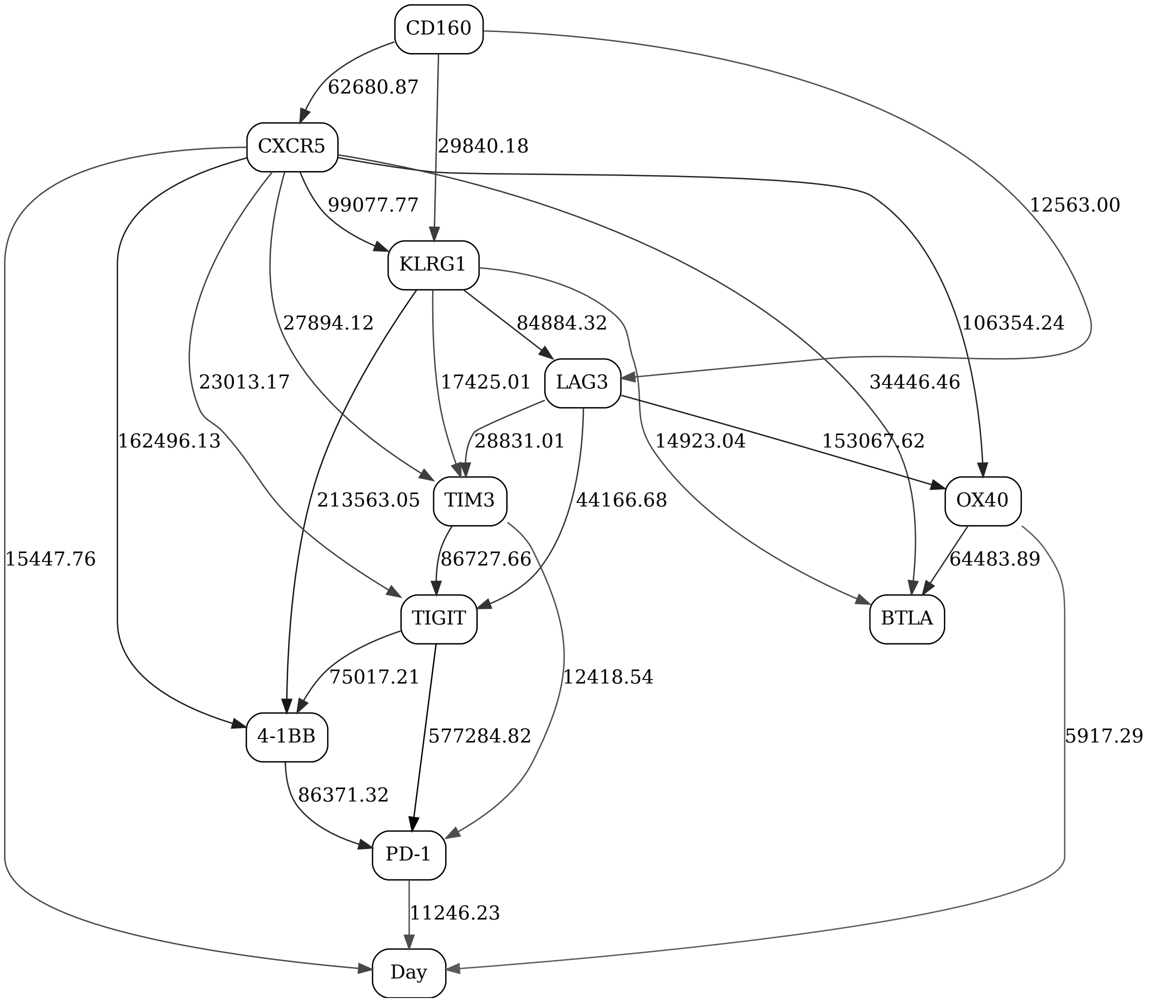

Figure 6: Checkpoint immune signaling network panel, naive CD4, day contrast (2-state "Day" variable), non-responders

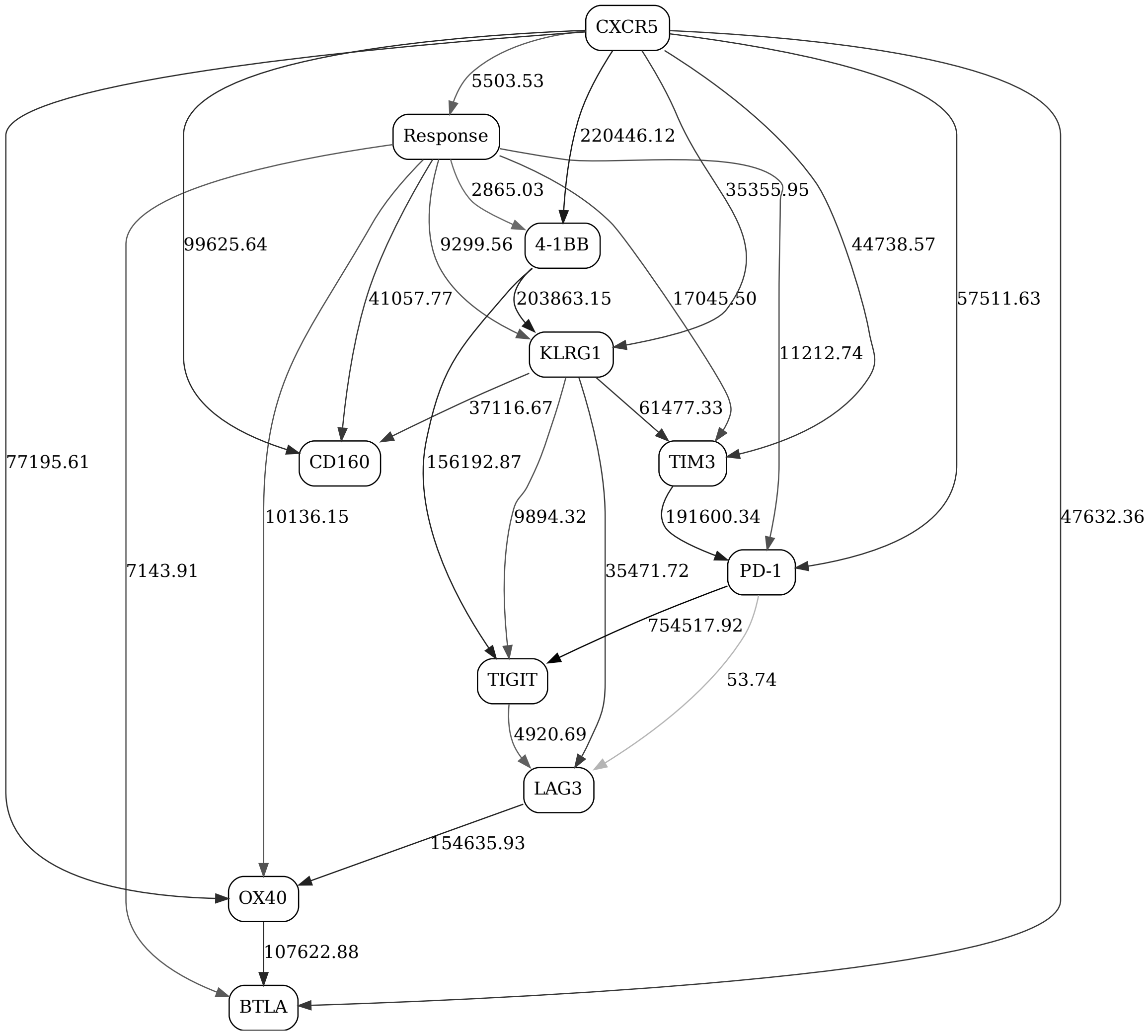

Figure 7: Checkpoint immune signaling network panel, naive CD4, day 1, response contrast (2-state "Response" variable)

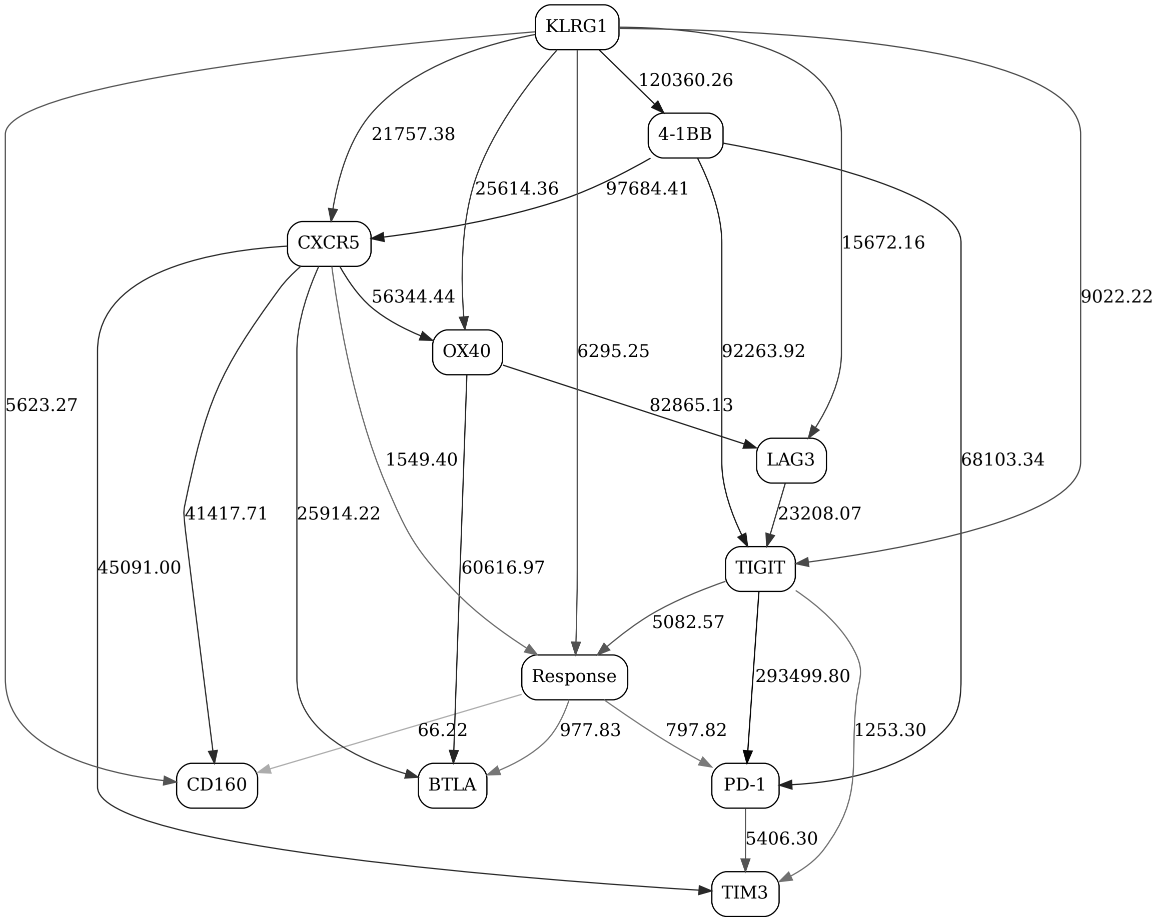

Figure 8: Checkpoint immune signaling network panel, naive CD4, day 21, response contrast (2-state "Response" variable)

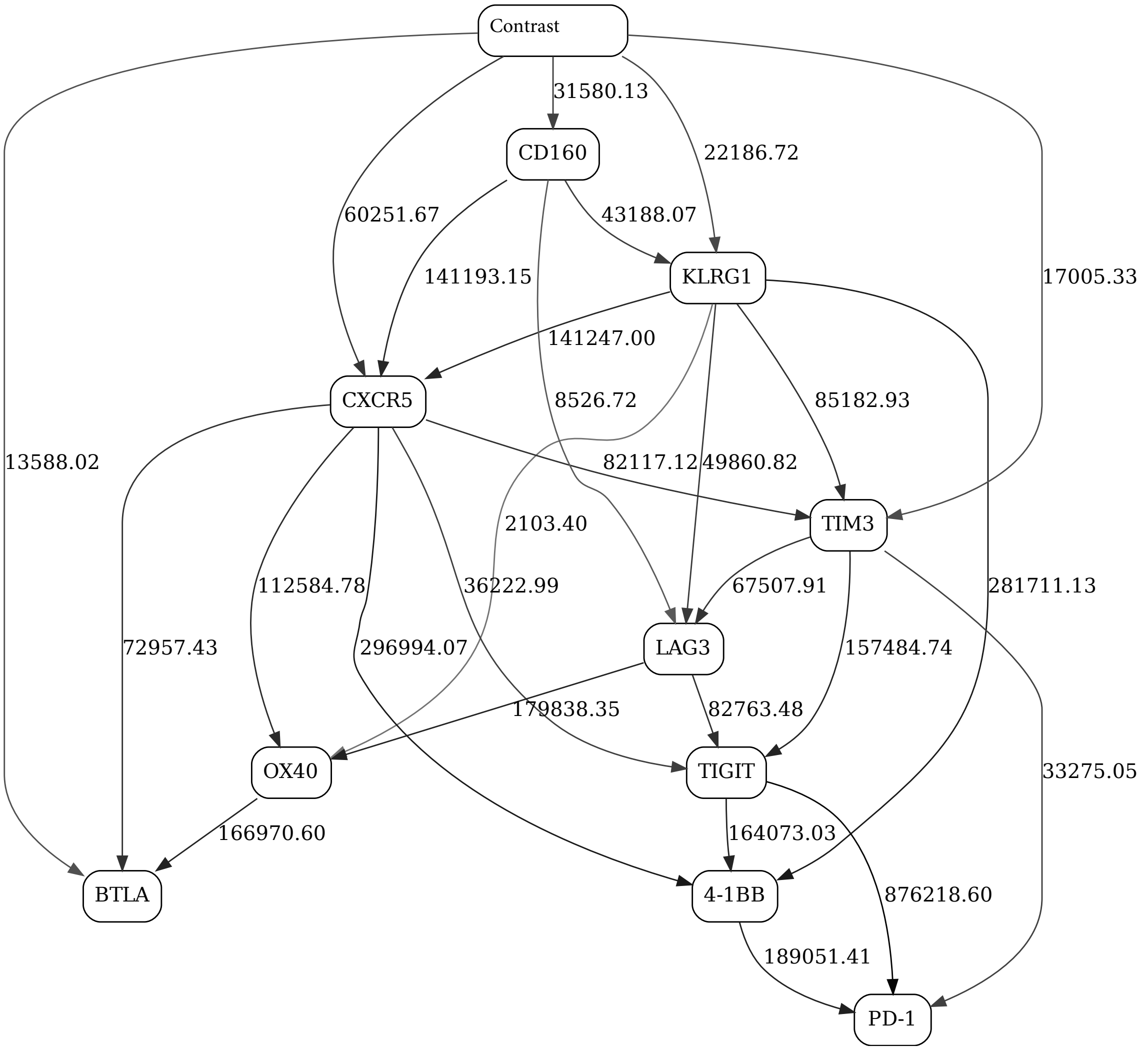

Figure 9: Checkpoint immune signaling network panel, naive CD4

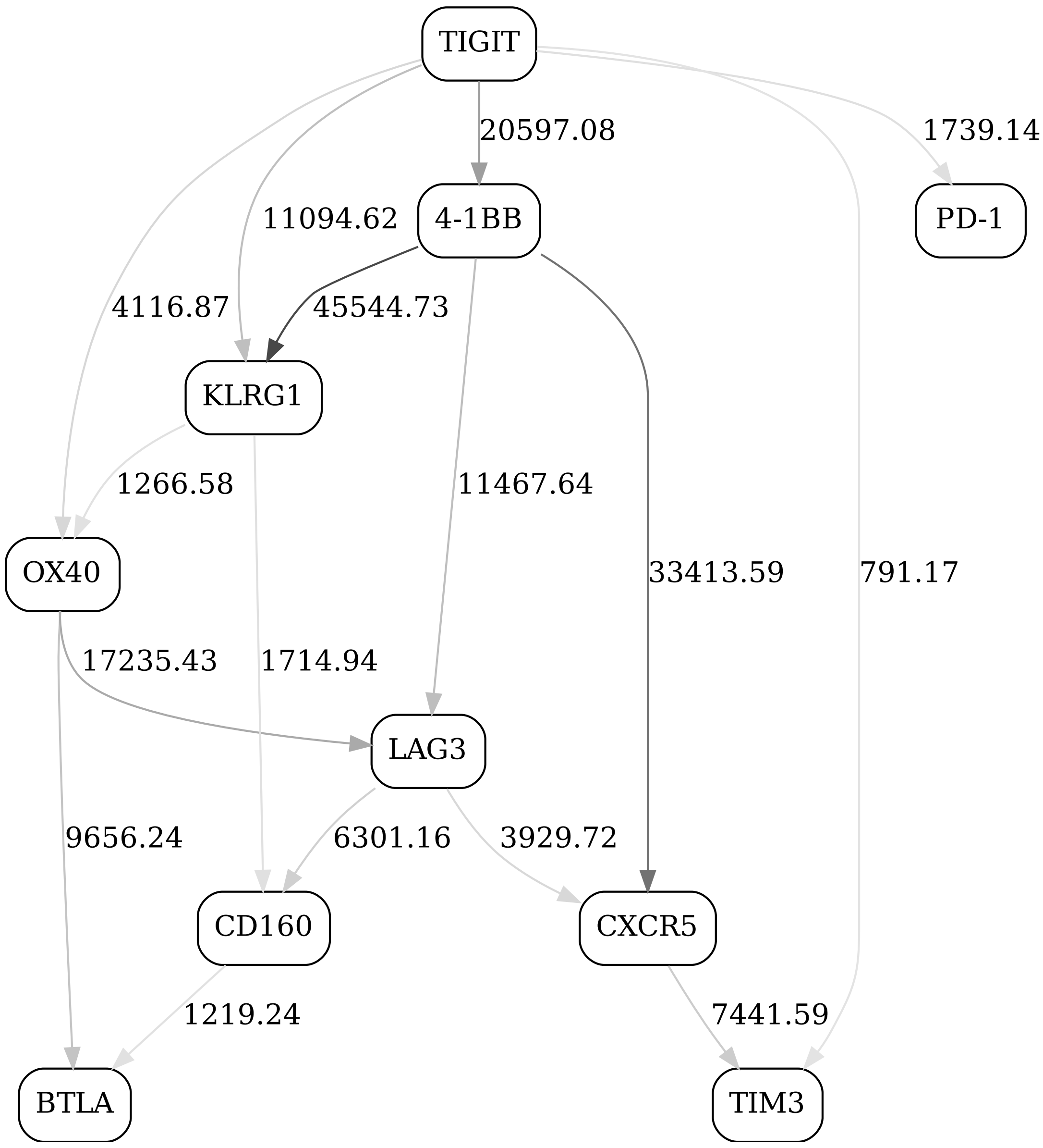

Figure 10: Checkpoint immune signaling network panel, non-naive CD8, day 1, responders

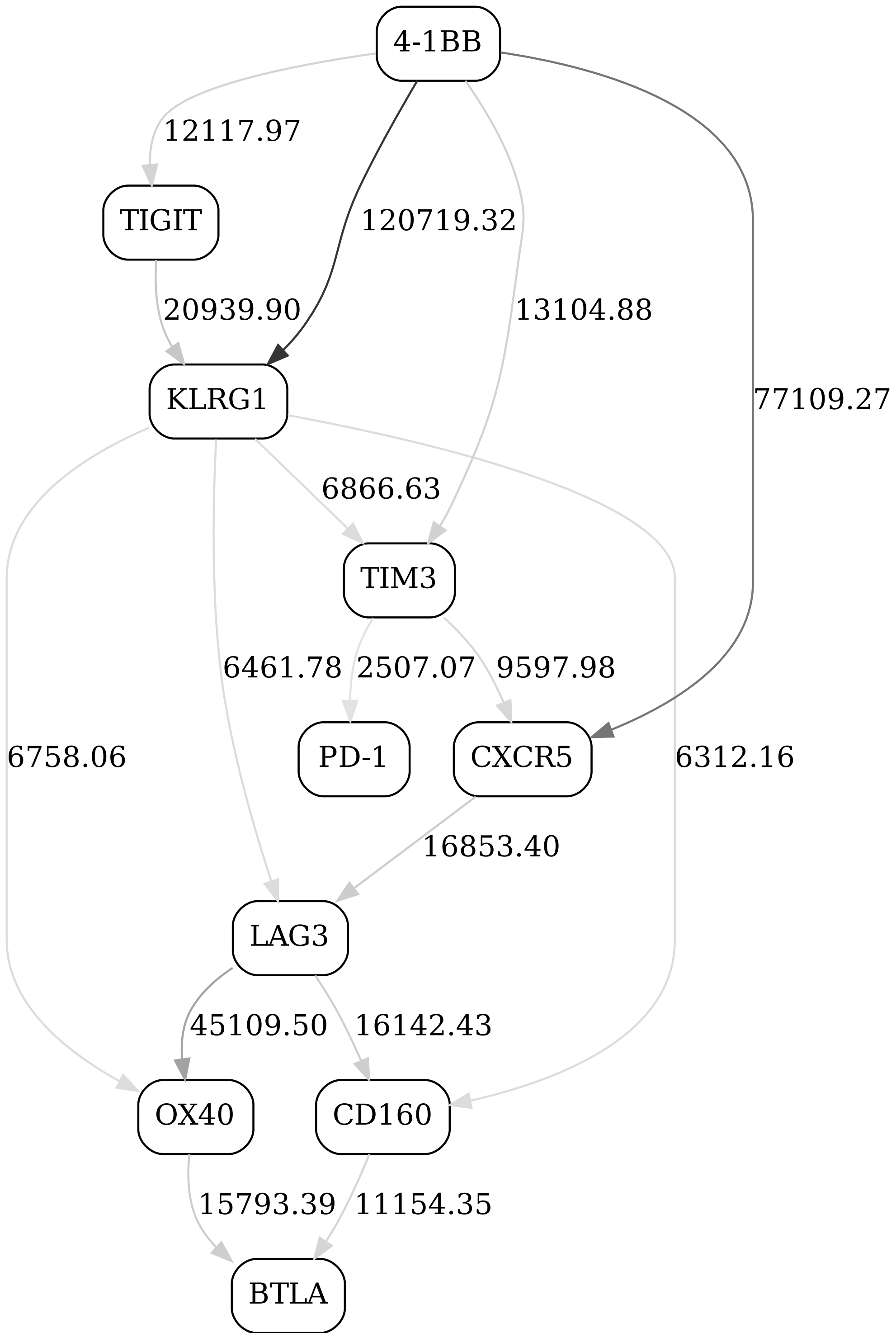

Figure 11: Checkpoint immune signaling network panel, non-naive CD8, day 1, non-responders

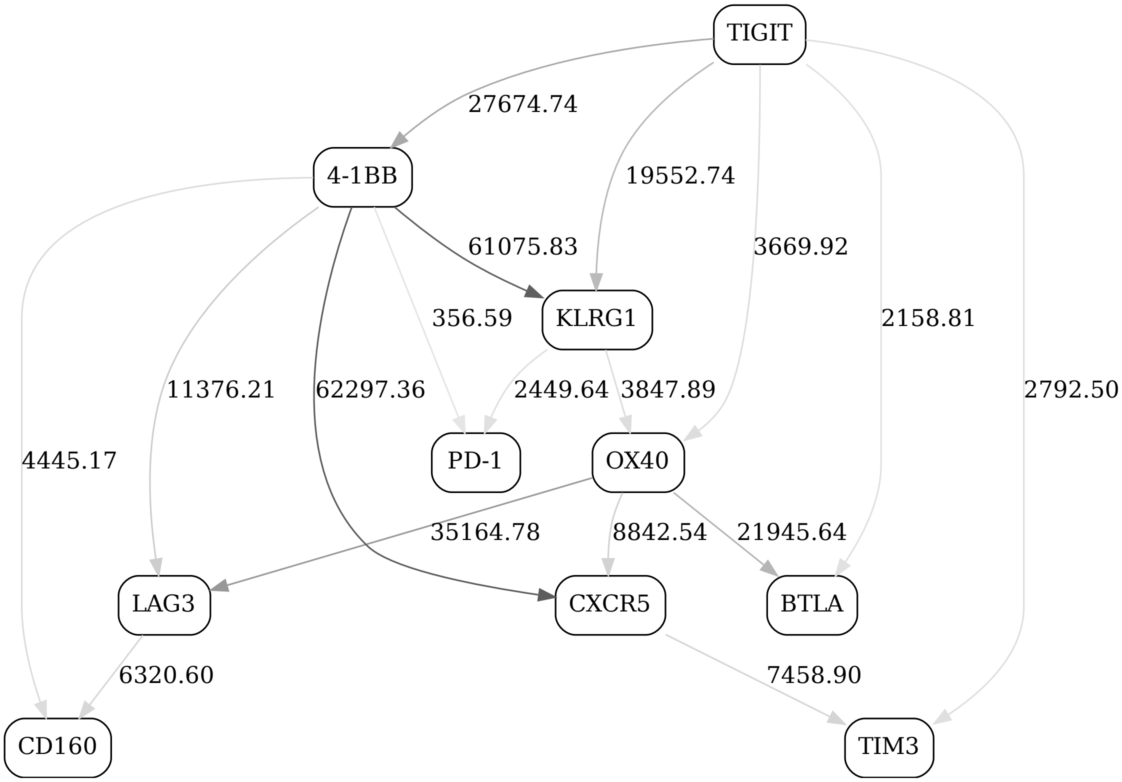

Figure 12: Checkpoint immune signaling network panel, non-naive CD8, day 21, responders

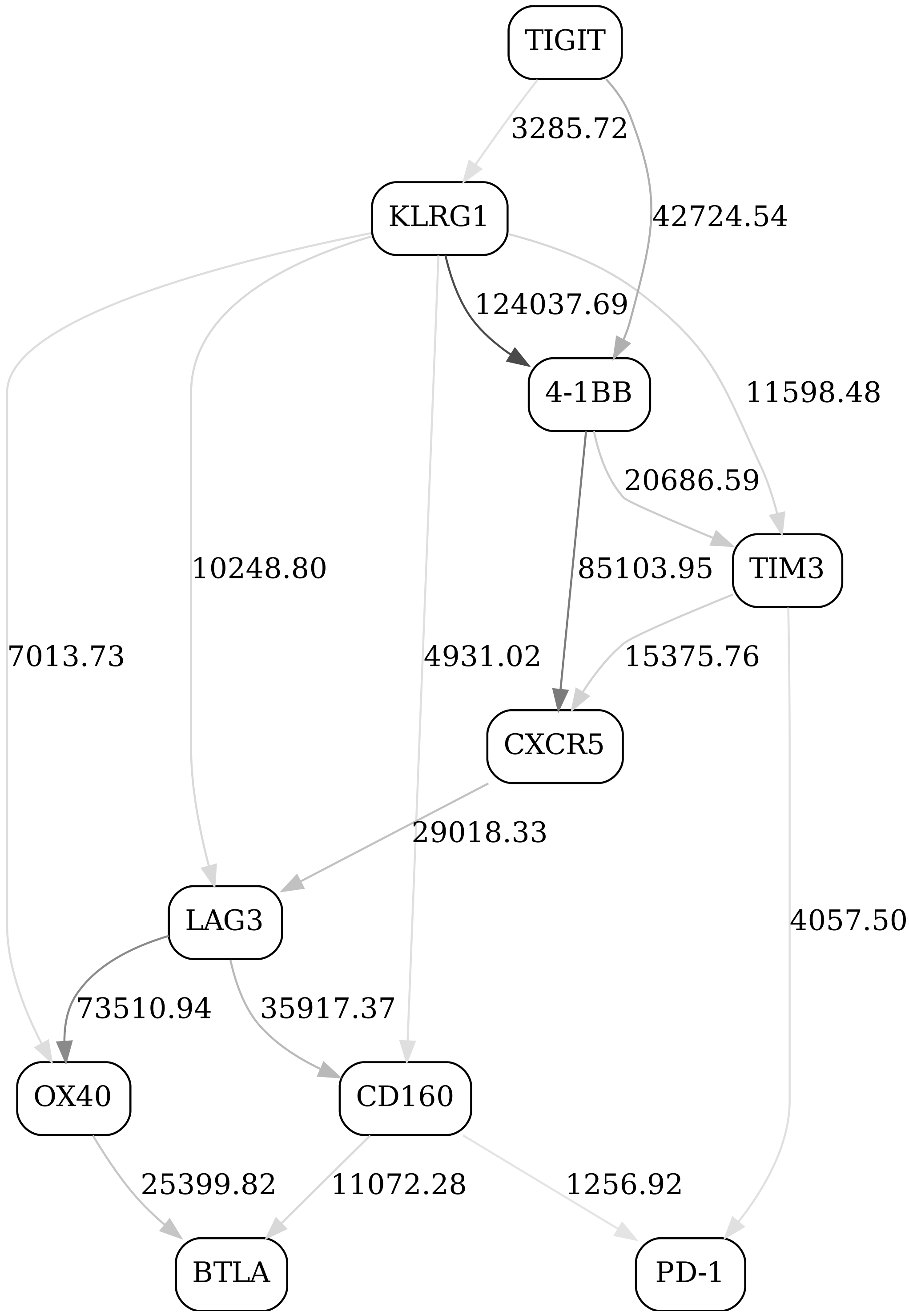

Figure 13: Checkpoint immune signaling network panel, non-naive CD8, day 21, non-responders

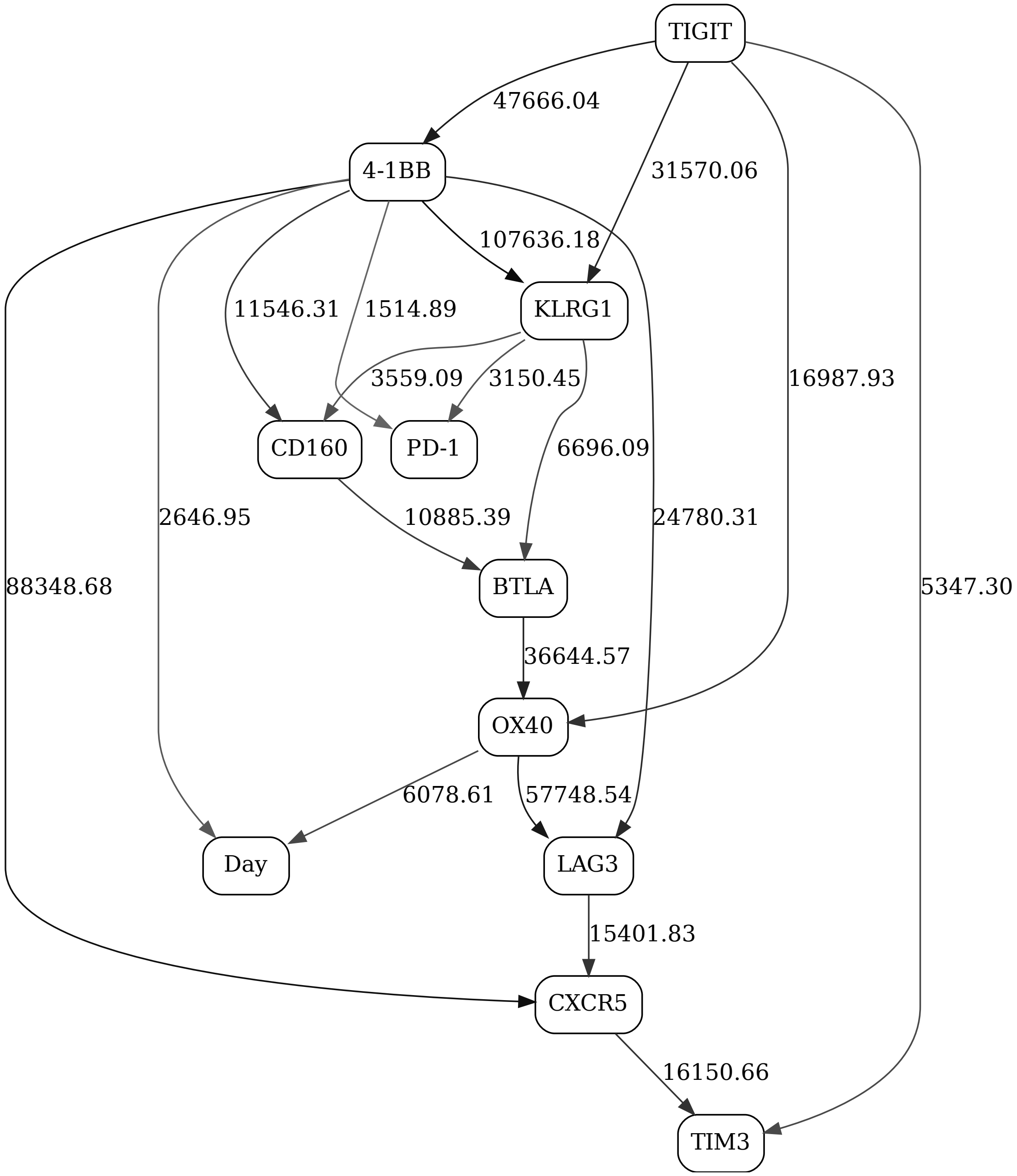

Figure 14: Checkpoint immune signaling network panel, non-naive CD8, day contrast (2-state "Day" variable), responders

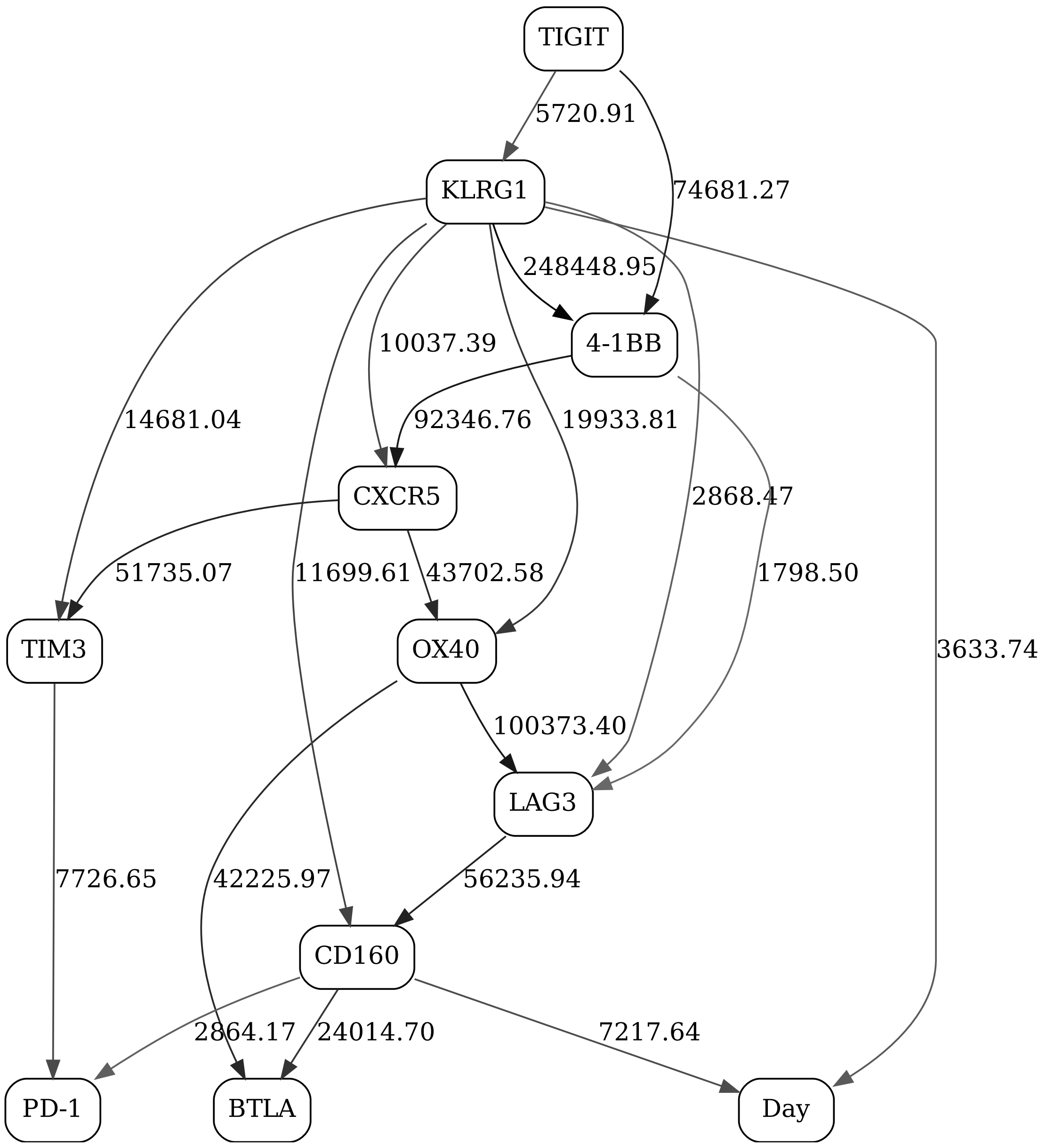

Figure 15: Checkpoint immune signaling network panel, non-naive CD8, day contrast (2-state ”Day” vari-able), non-responders

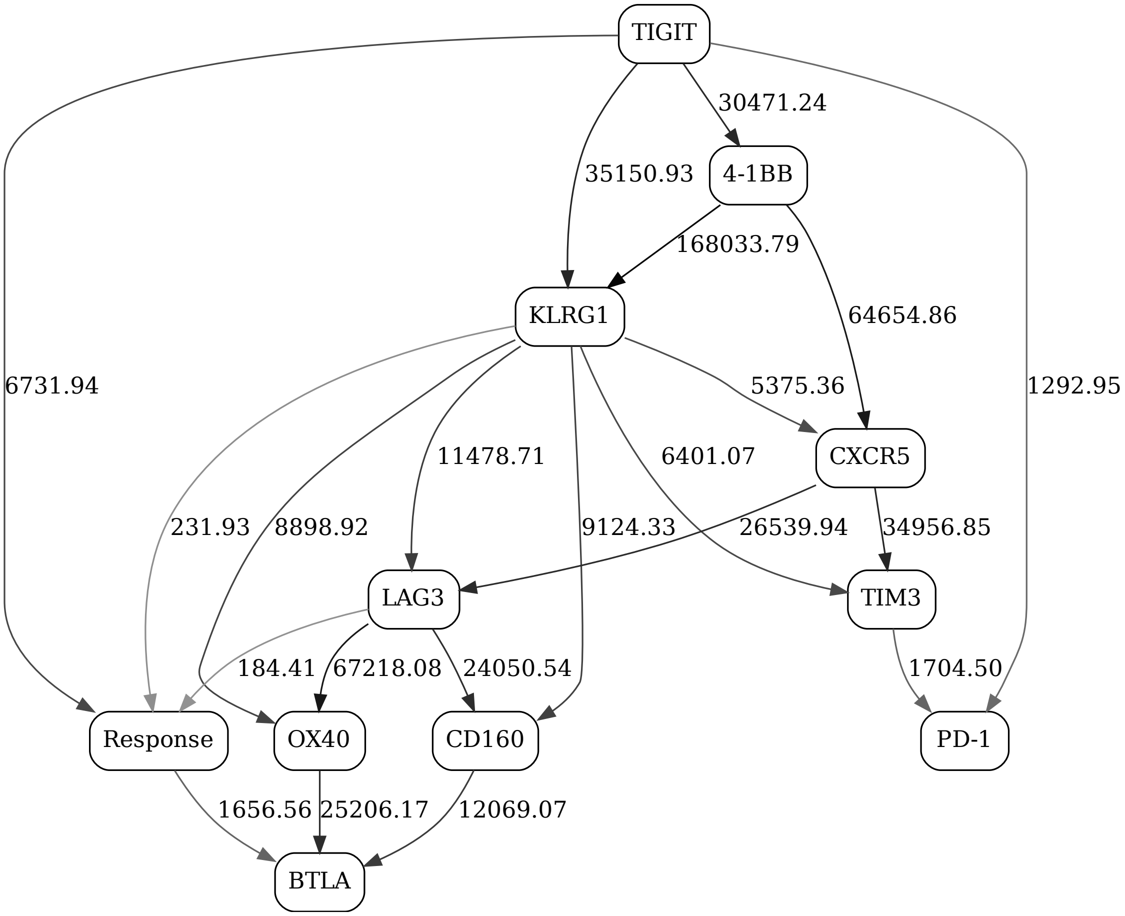

Figure 16: Checkpoint immune signaling network panel, non-naive CD8, day 1, response contrast (2-state "Response" variable)

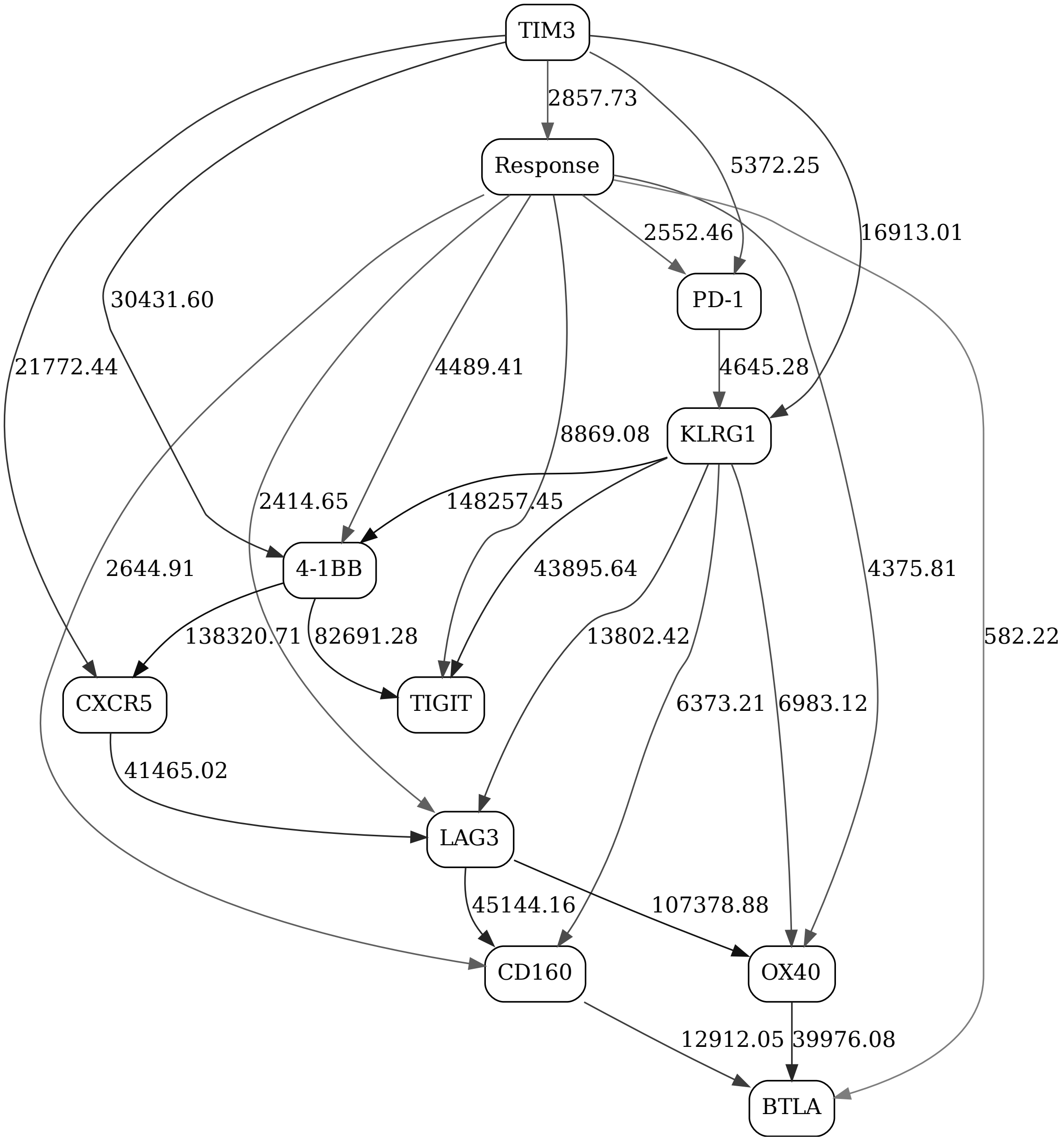

Figure 17: Checkpoint immune signaling network panel, non-naive CD8, day 21, response contrast (2-state ”Response” variable)

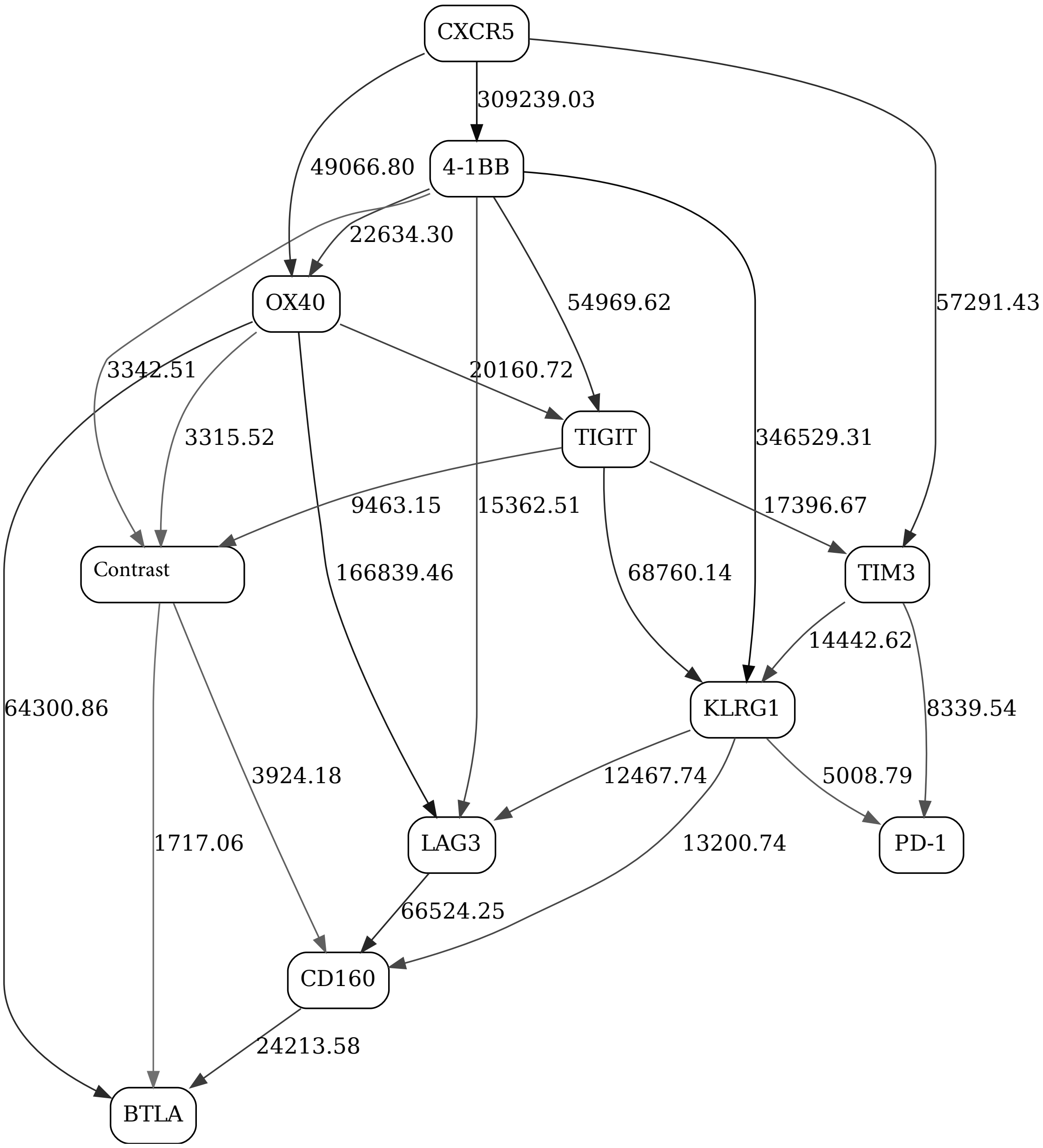

Figure 18: Checkpoint immune signaling network panel, non-naive CD8

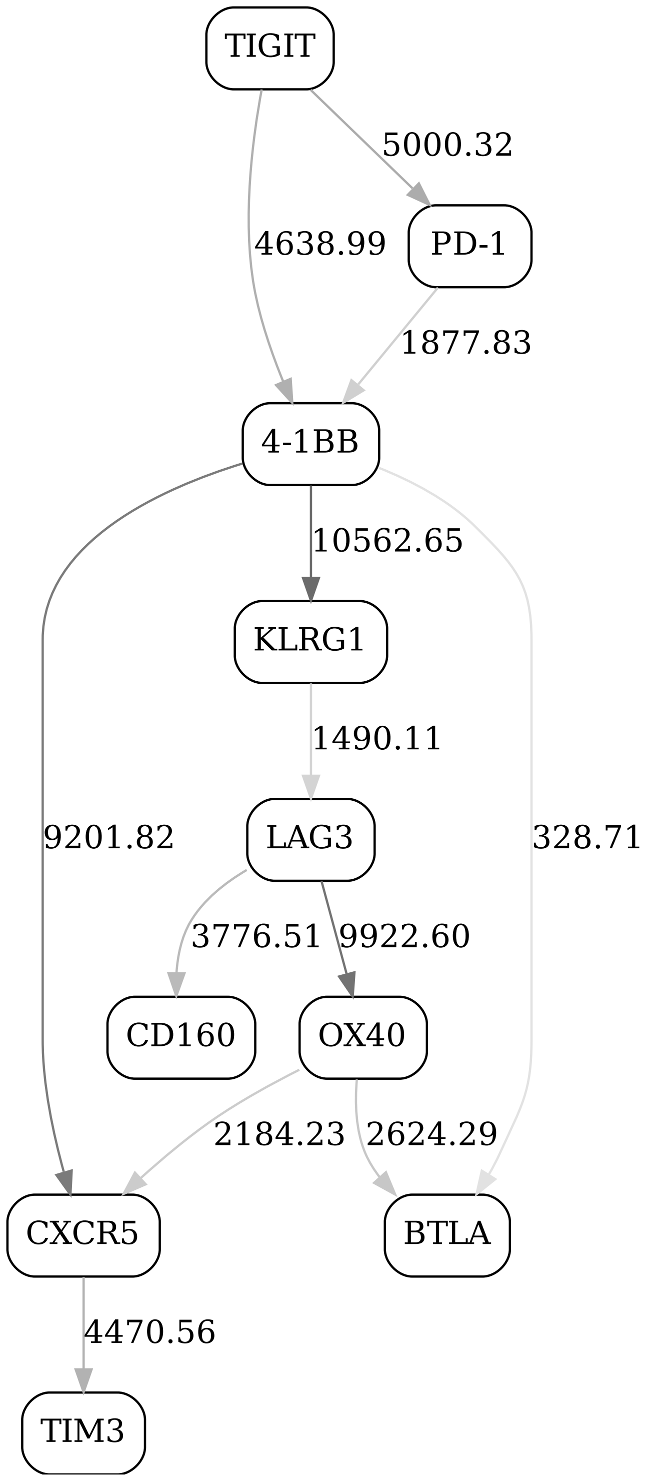

Figure 19: Checkpoint immune signaling network panel, naive CD8, day 1, responders

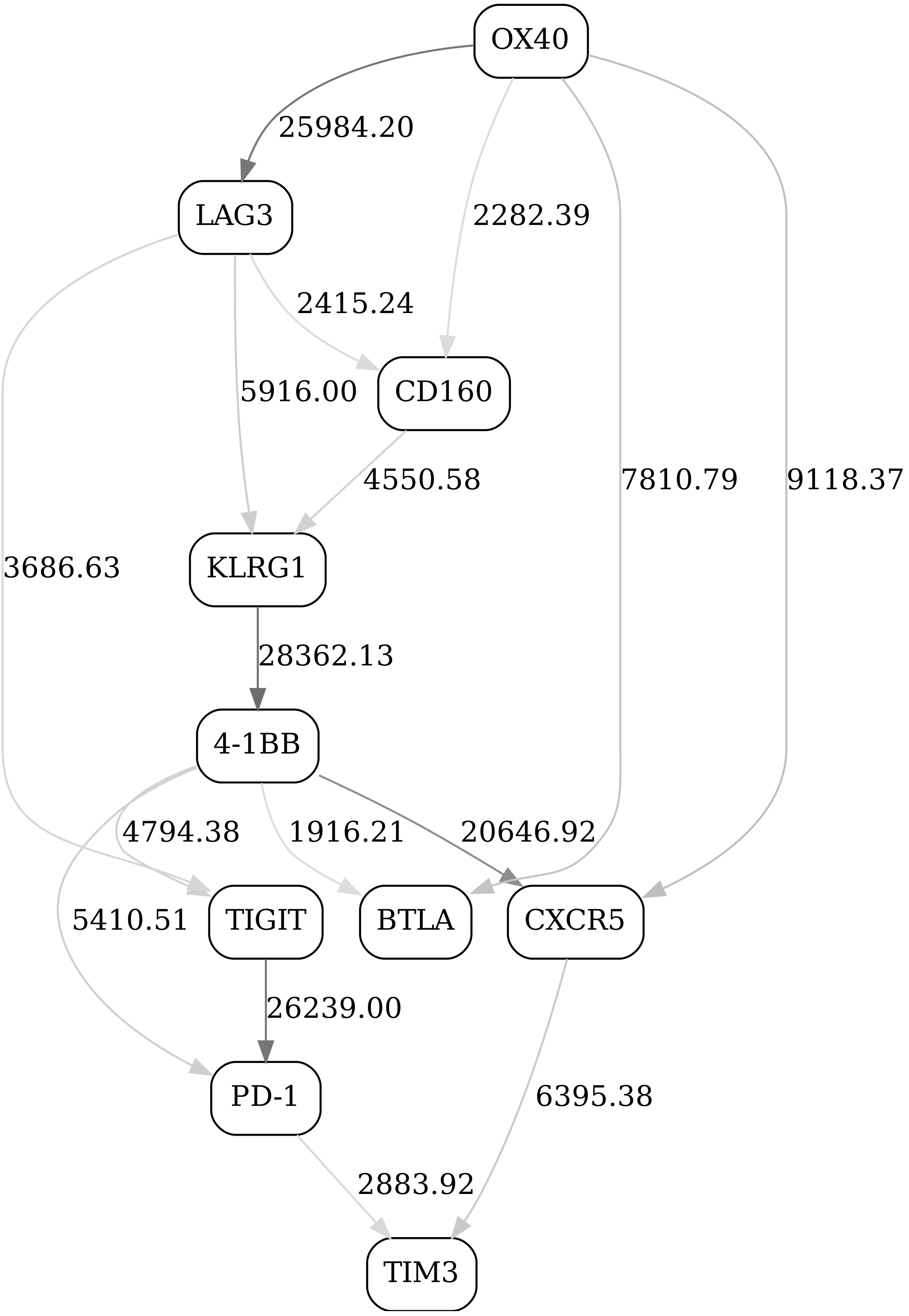

Figure 20: Checkpoint immune signaling network panel, naive CD8, day 1, non-responders

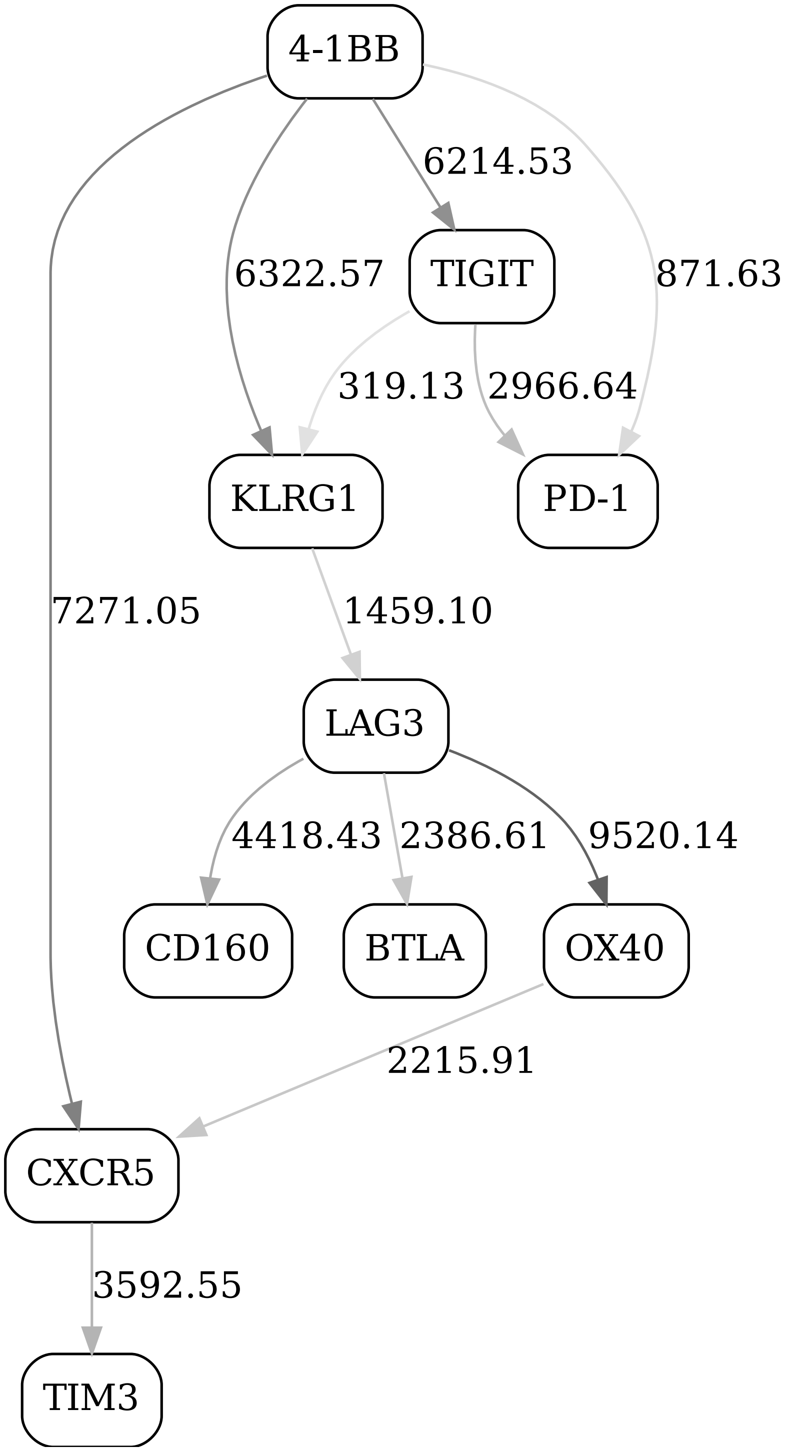

Figure 21: Checkpoint immune signaling network panel, naive CD8, day 21, responders

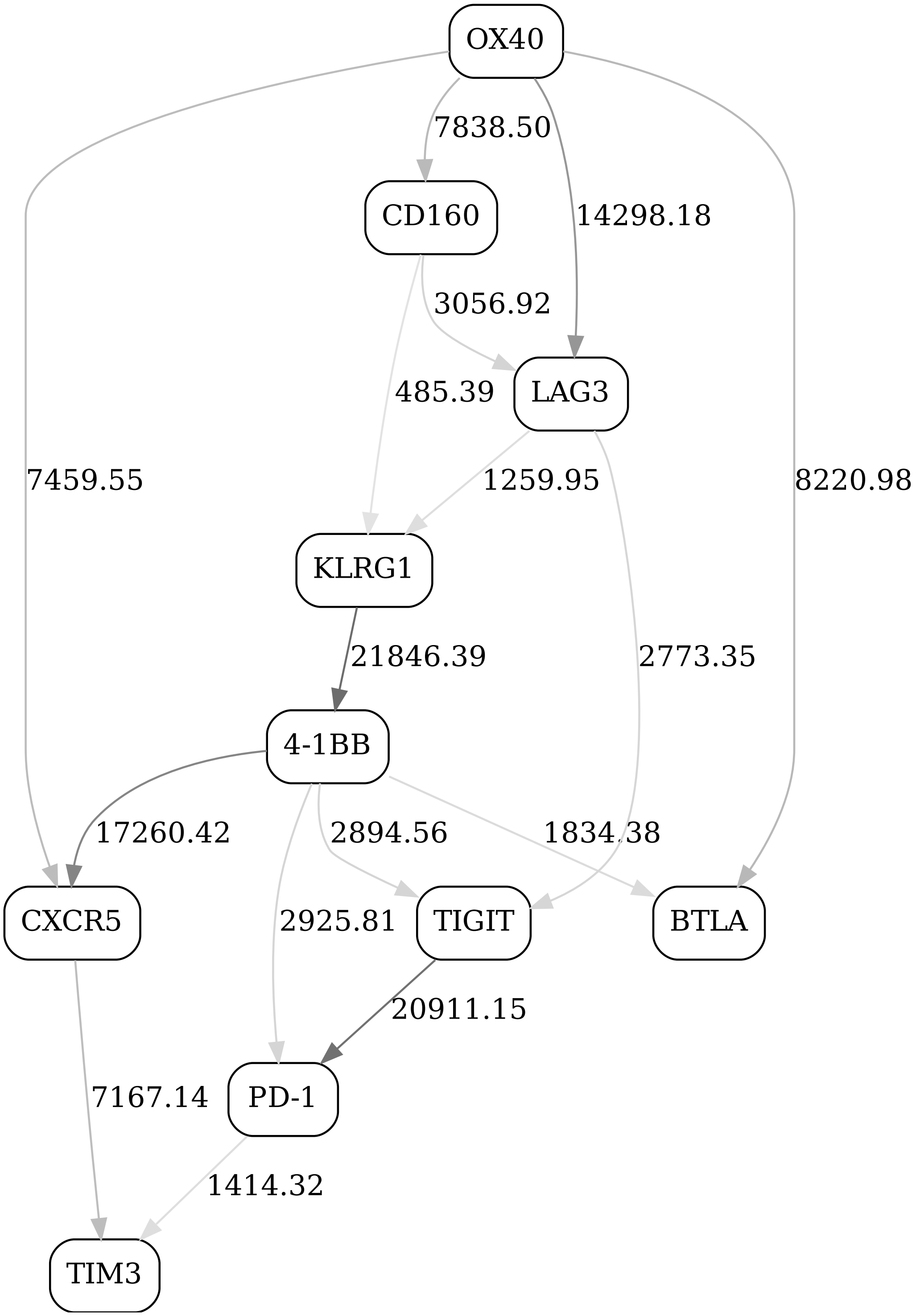

Figure 22: Checkpoint immune signaling network panel, naive CD8, day 21, non-responders

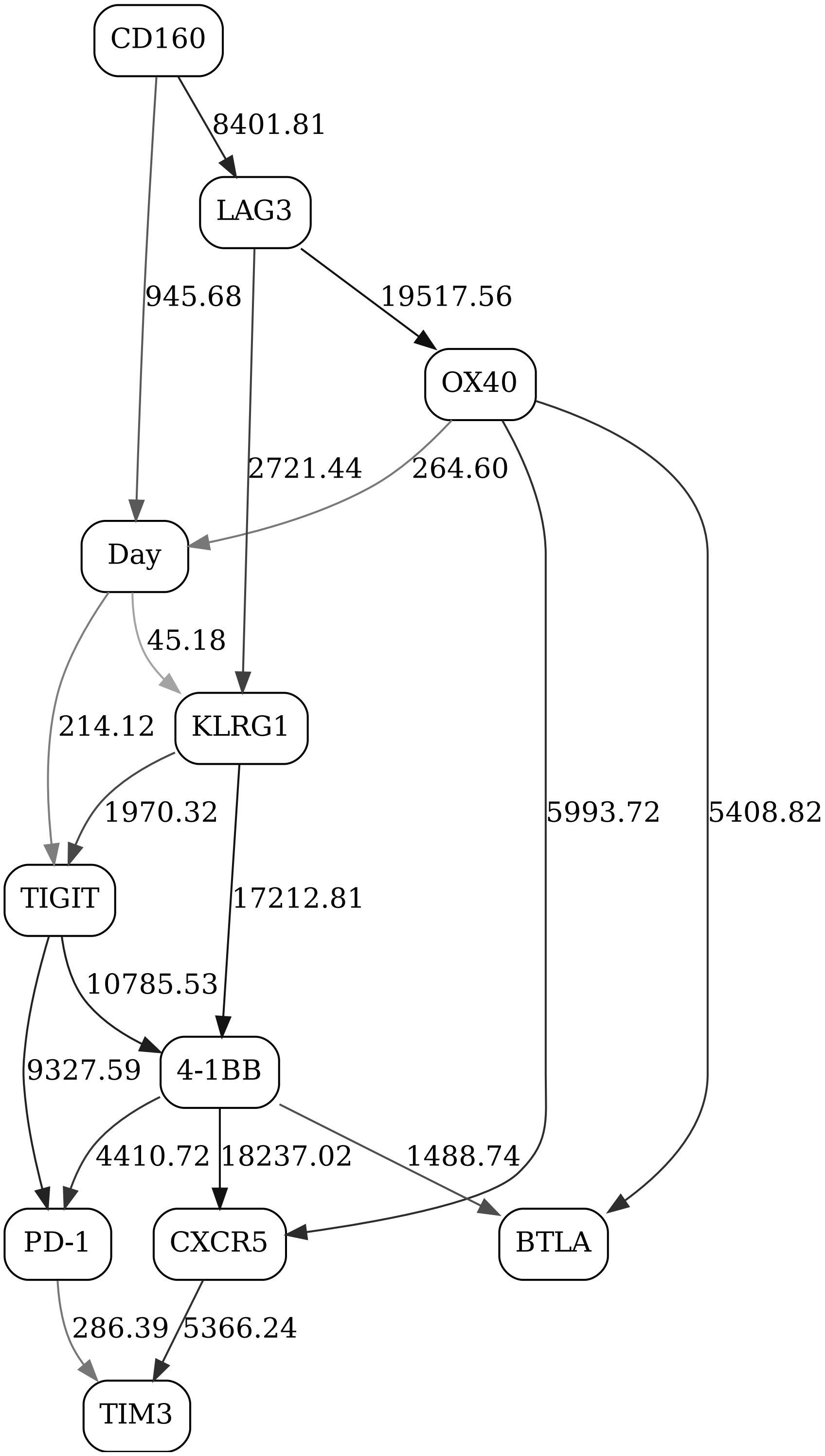

Figure 23: Checkpoint immune signaling network panel, naive CD8, day contrast (2-state "Day" variable), responders

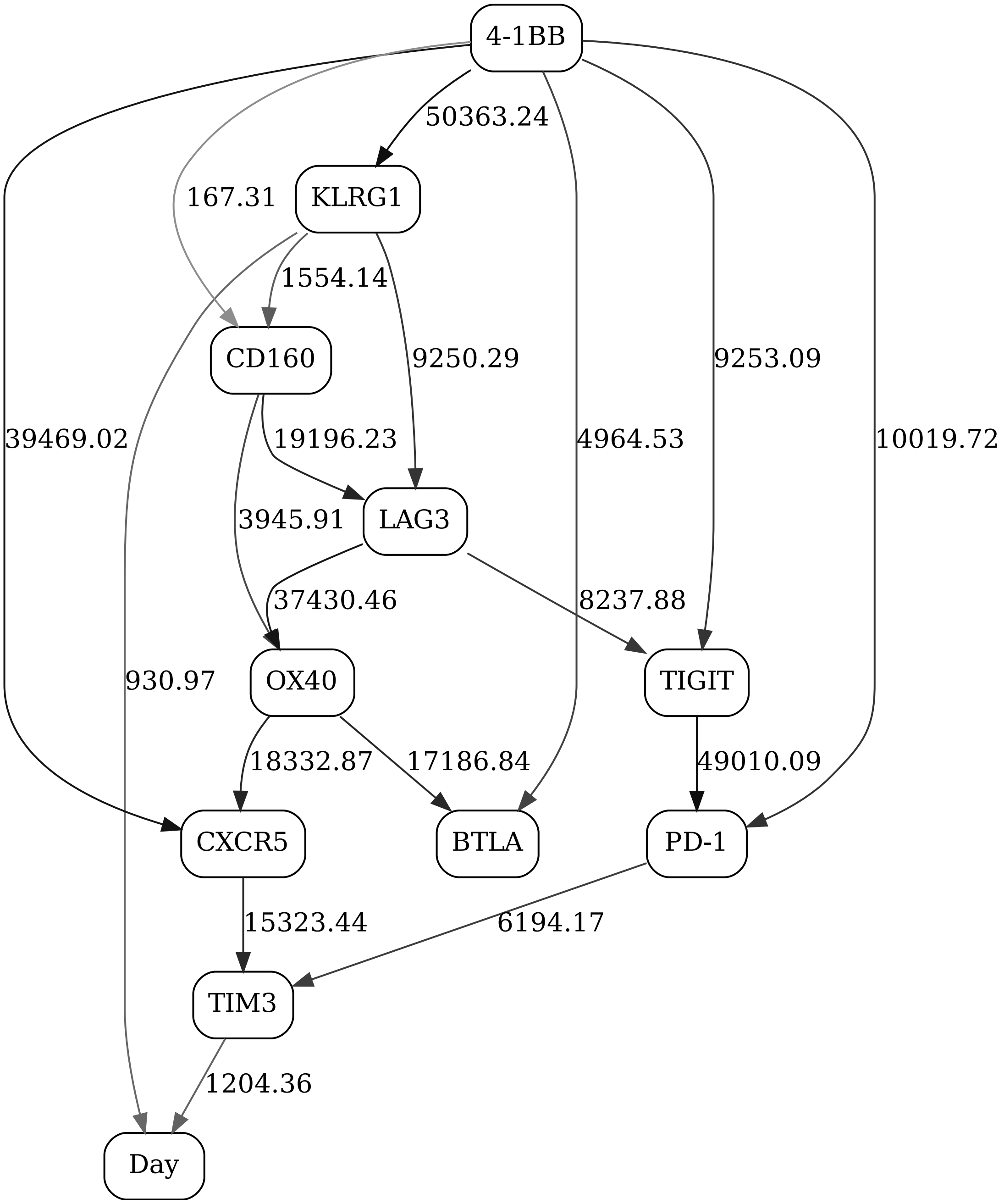

Figure 24: Checkpoint immune signaling network panel, naive CD8, day contrast (2-state "Day" variable), non-responders

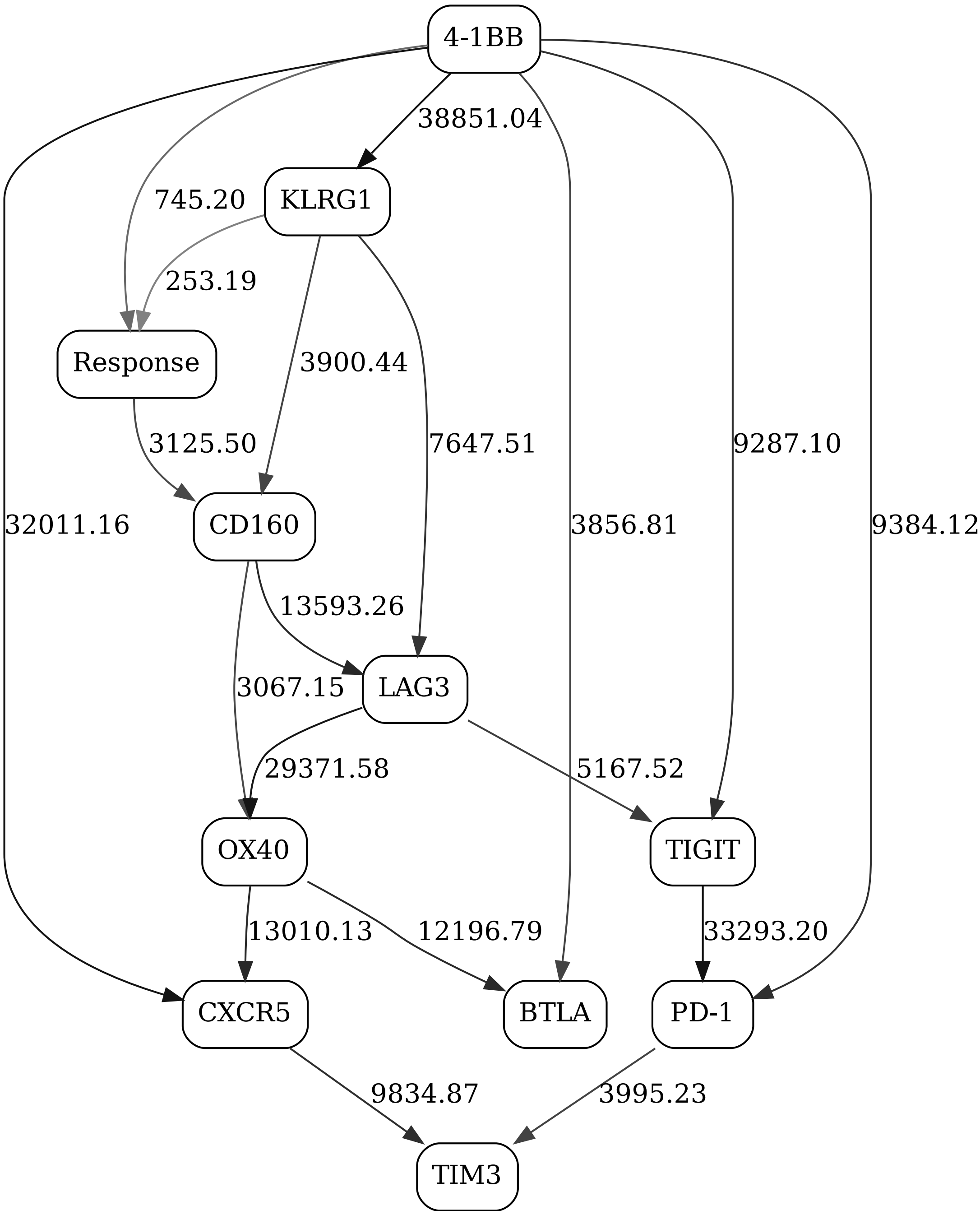

Figure 25: Checkpoint immune signaling network panel, naive CD8, day 1, response contrast (2-state "Response" variable)

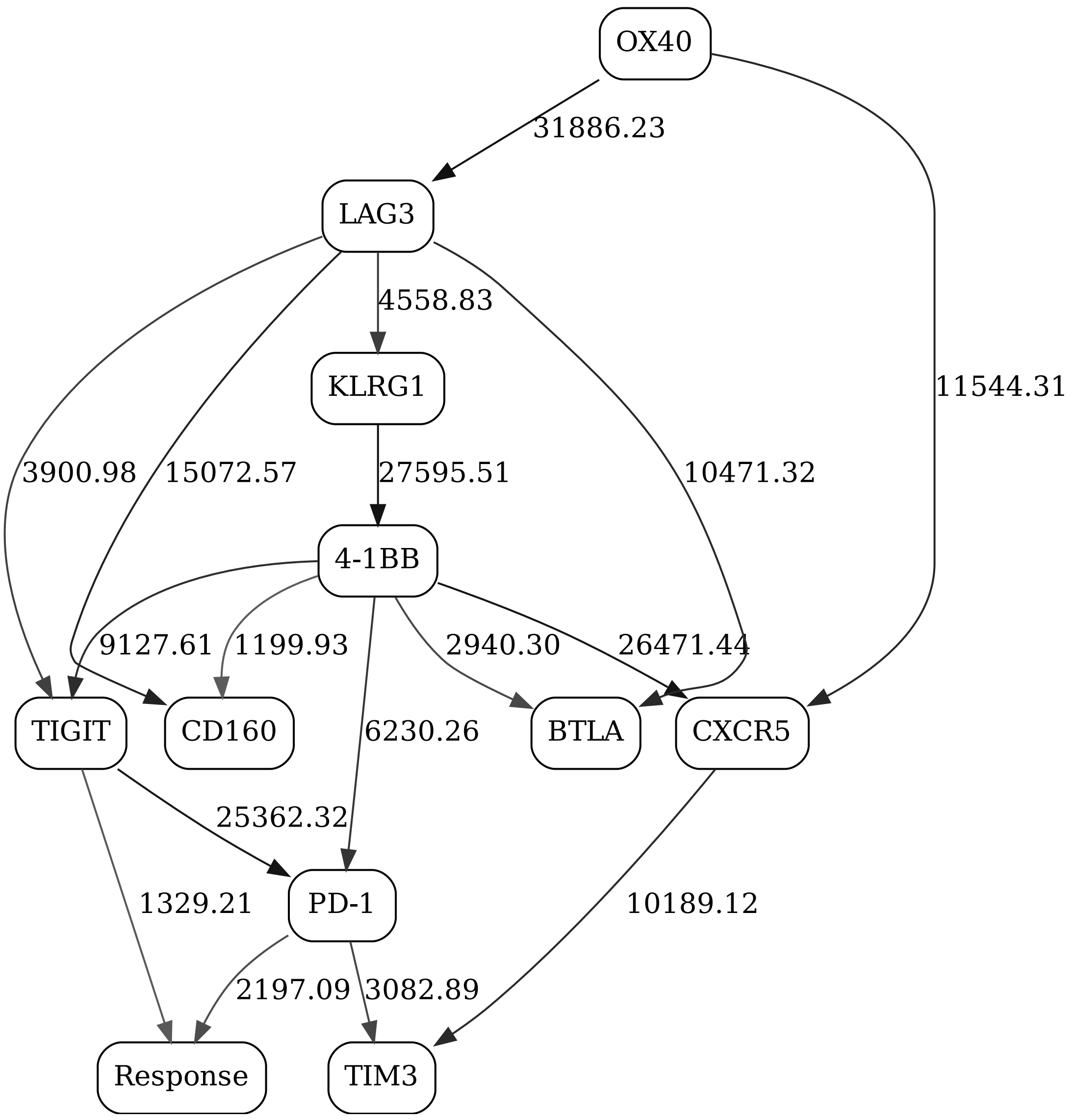

Figure 26: Checkpoint immune signaling network panel, naive CD8, day 21, response contrast (2-state "Response" variable)

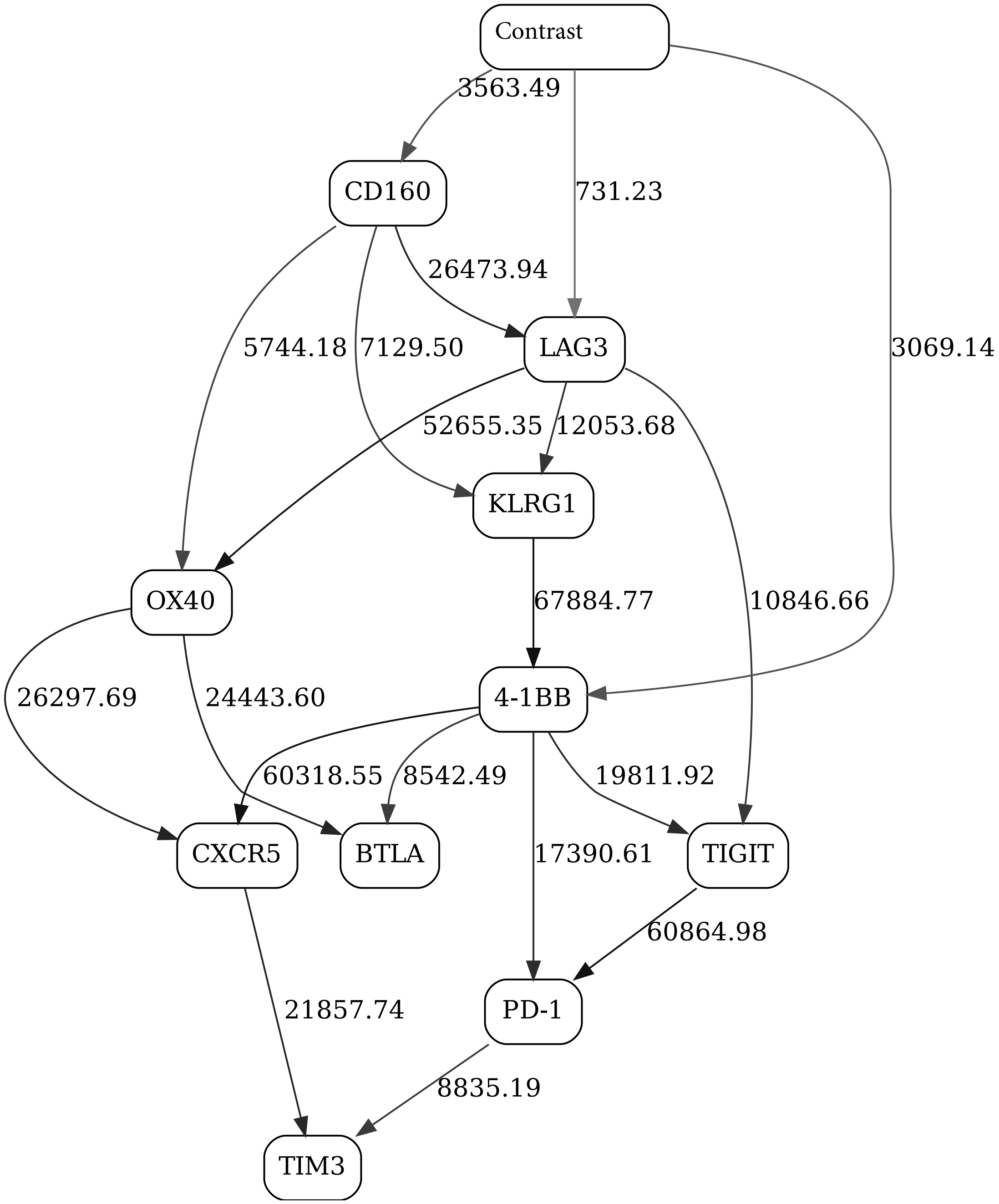

Figure 27: Checkpoint immune signaling network panel, naive CD8

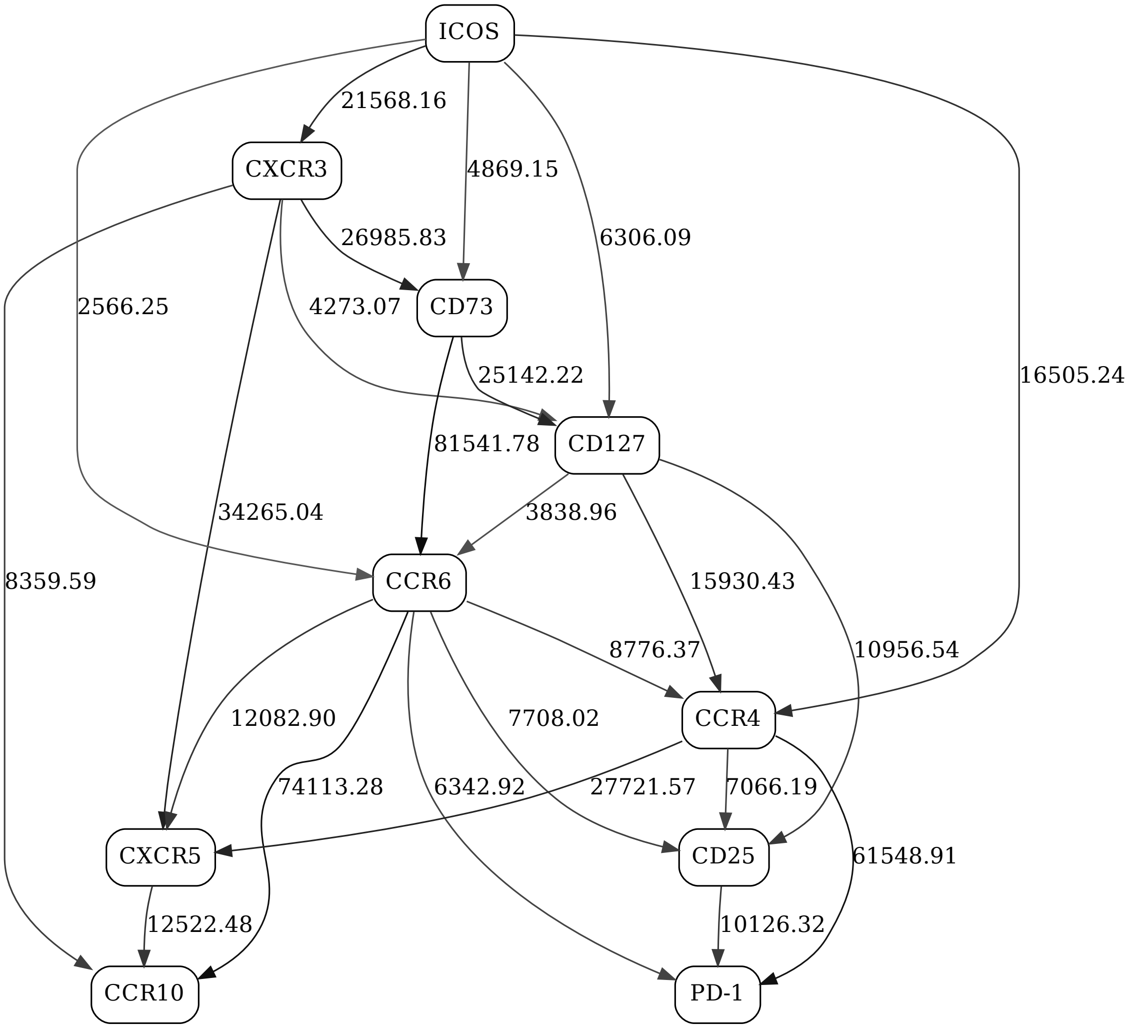

Figure 28: Adaptive immune signaling network panel, non-naive CD4, day 1, responders

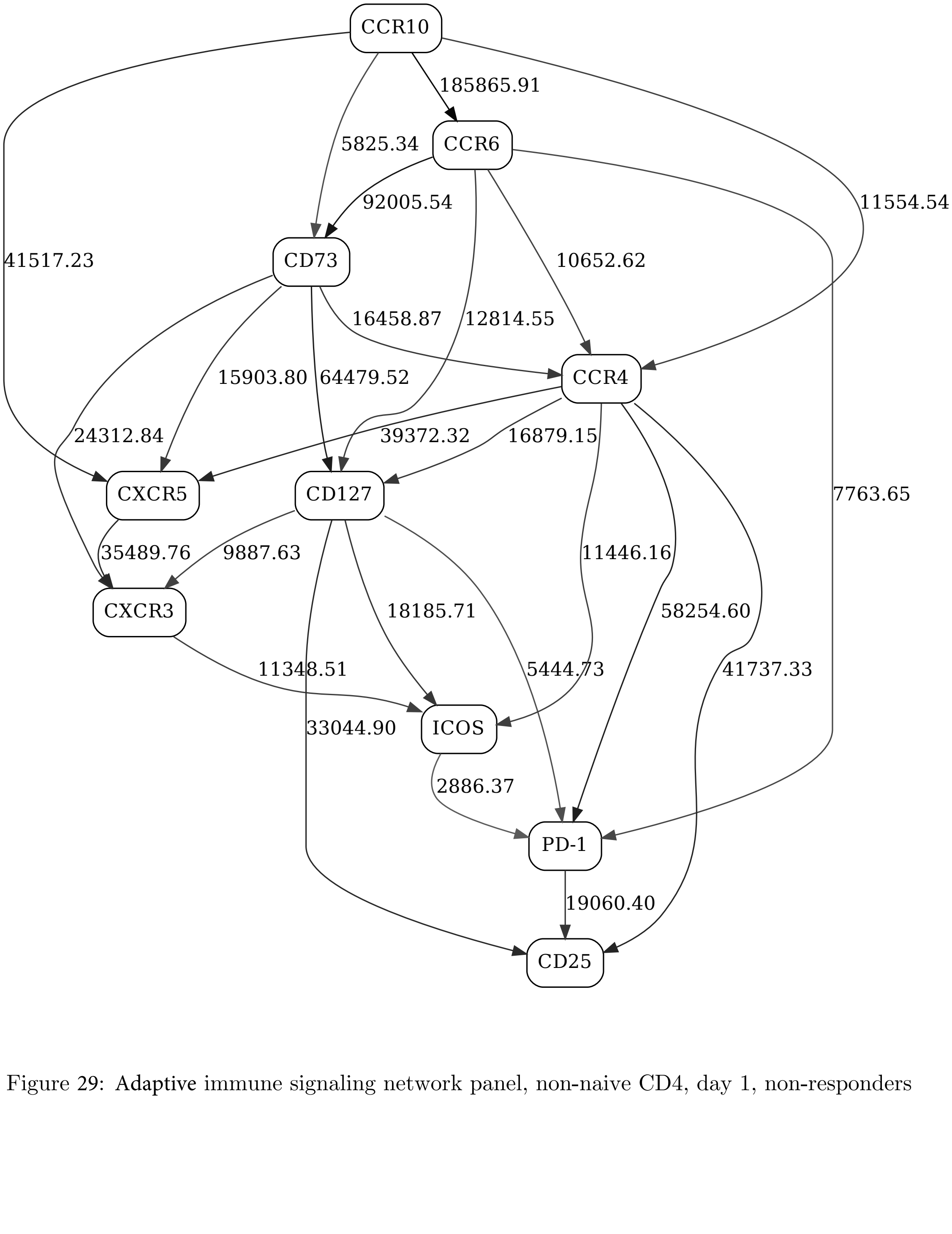

Figure 29: Adaptive immune signaling network panel, non-naive CD4, day 1, non-responders

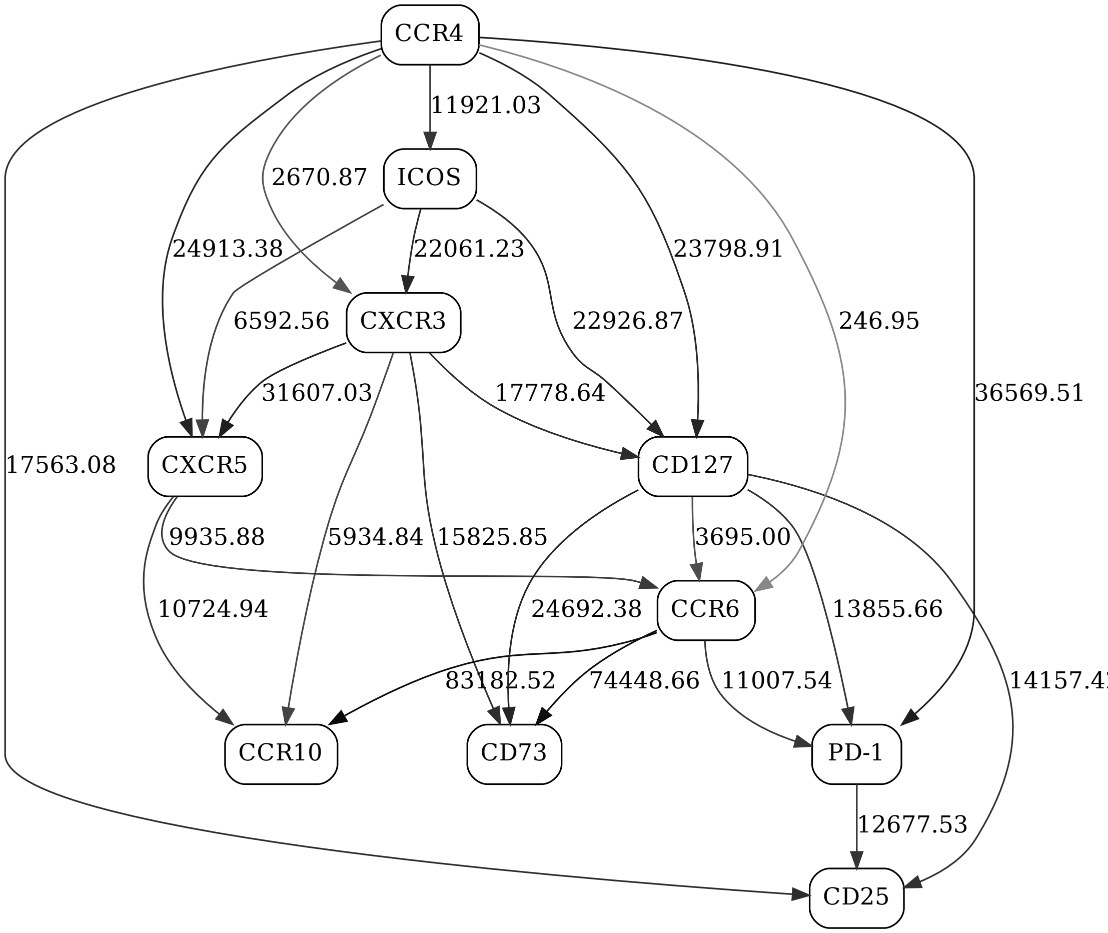

Figure 30: Adaptive immune signaling network panel, non-naive CD4, day 21, responders

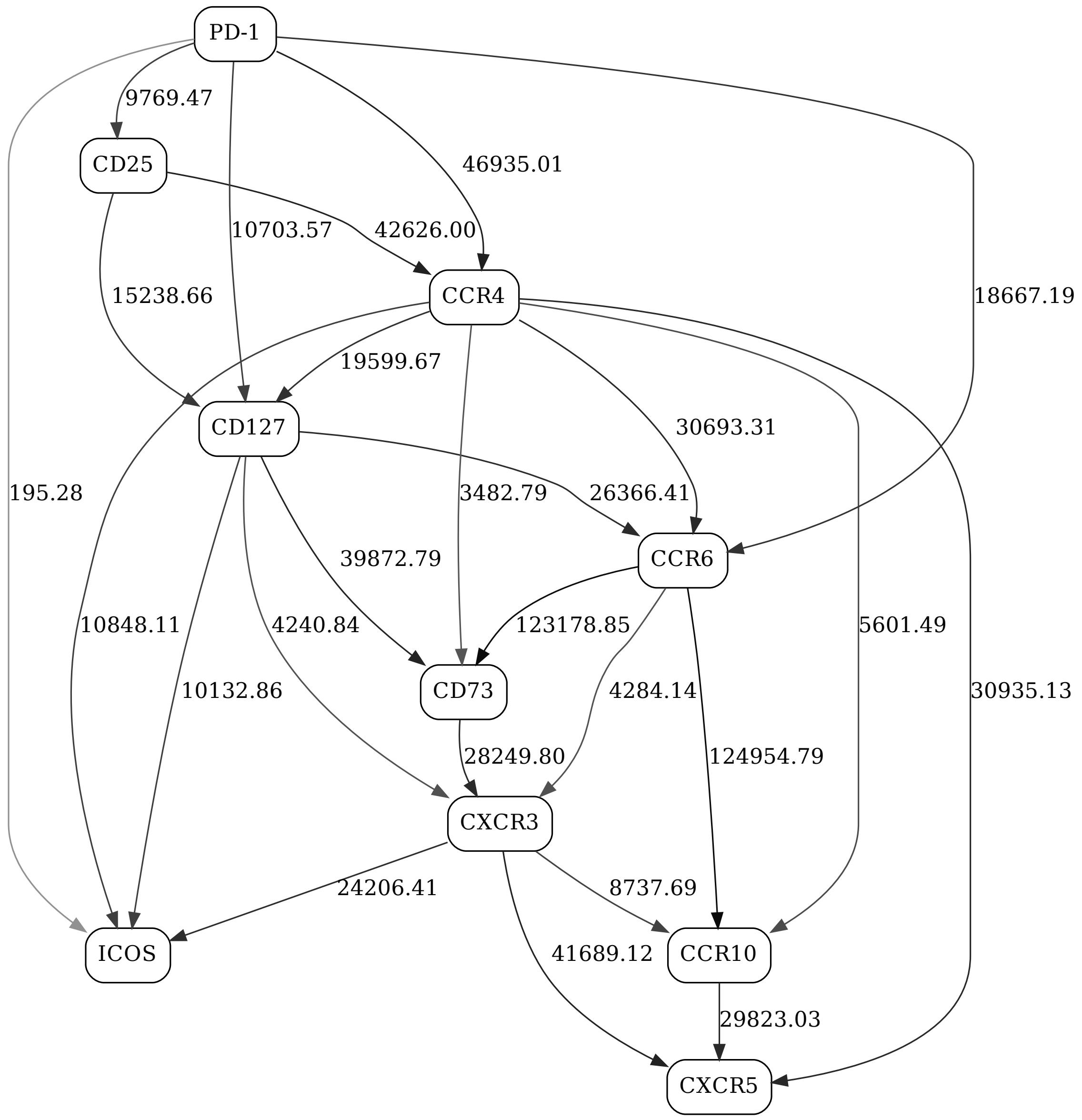

Figure 31: Adaptive immune signaling network panel, non-naive CD4, day 21, non-responders

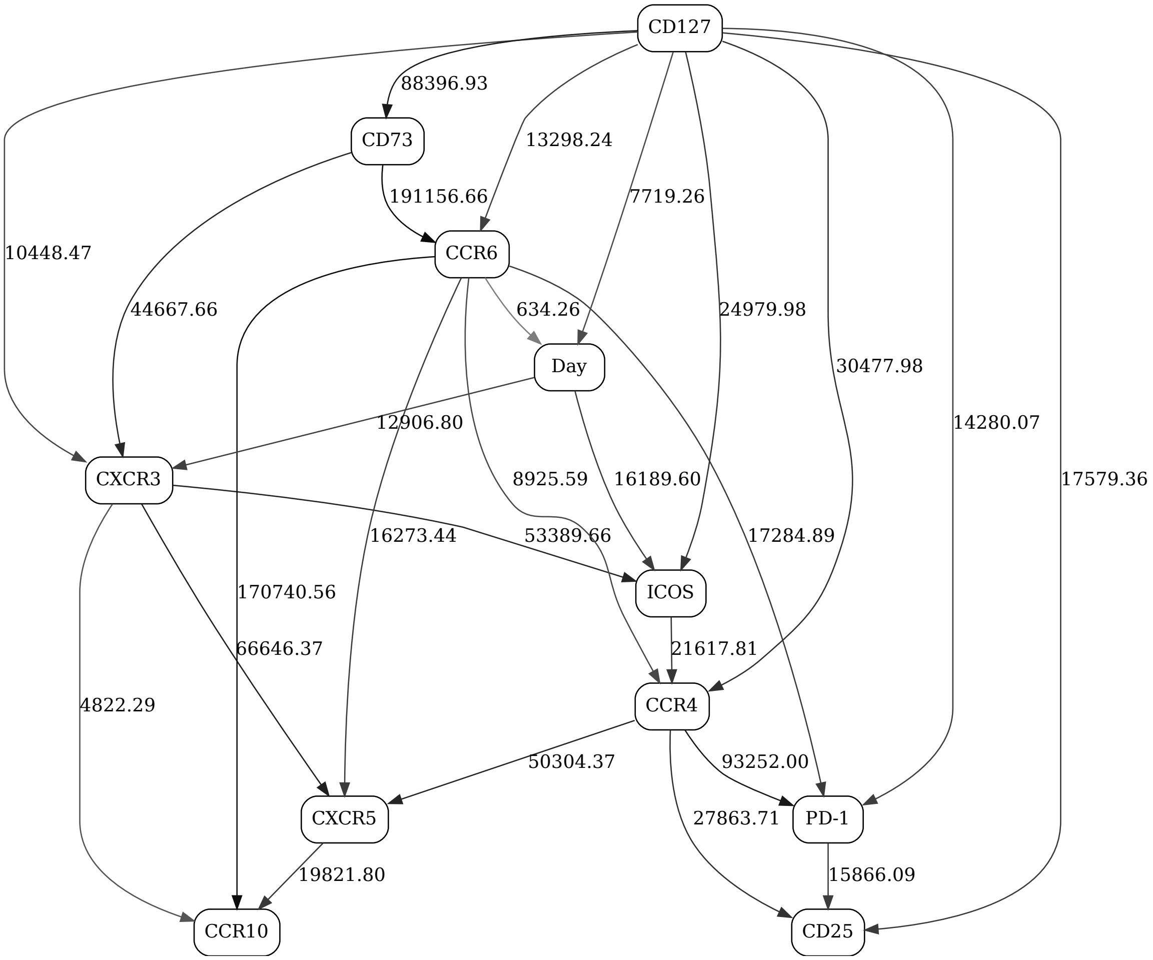

Figure 32: Adaptive immune signaling network panel, non-naive CD4, day contrast (2-state "Day" variable), responders

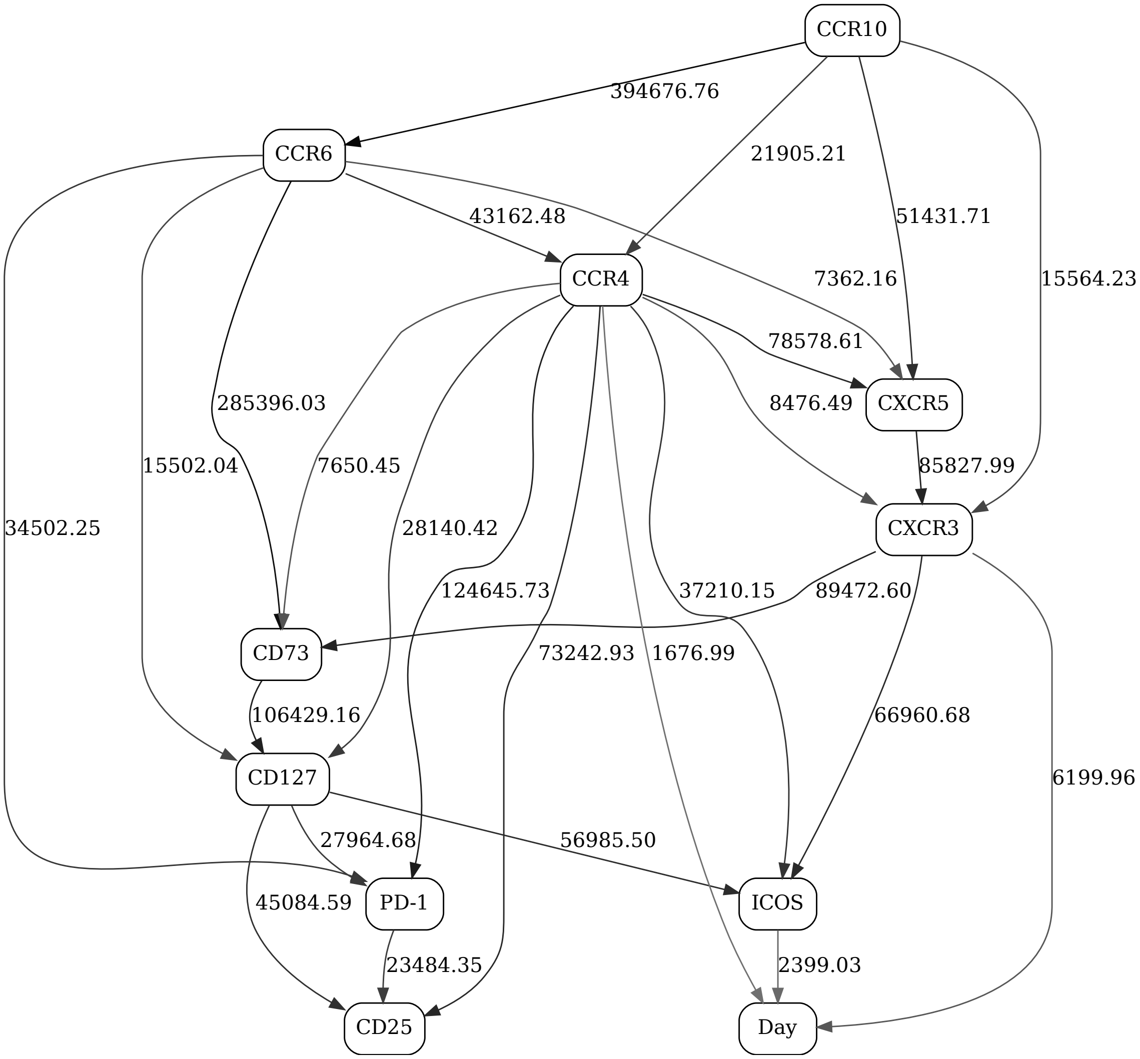

Figure 33: Adaptive immune signaling network panel, non-naive CD4, day contrast (2-state "Day" variable), non-responders

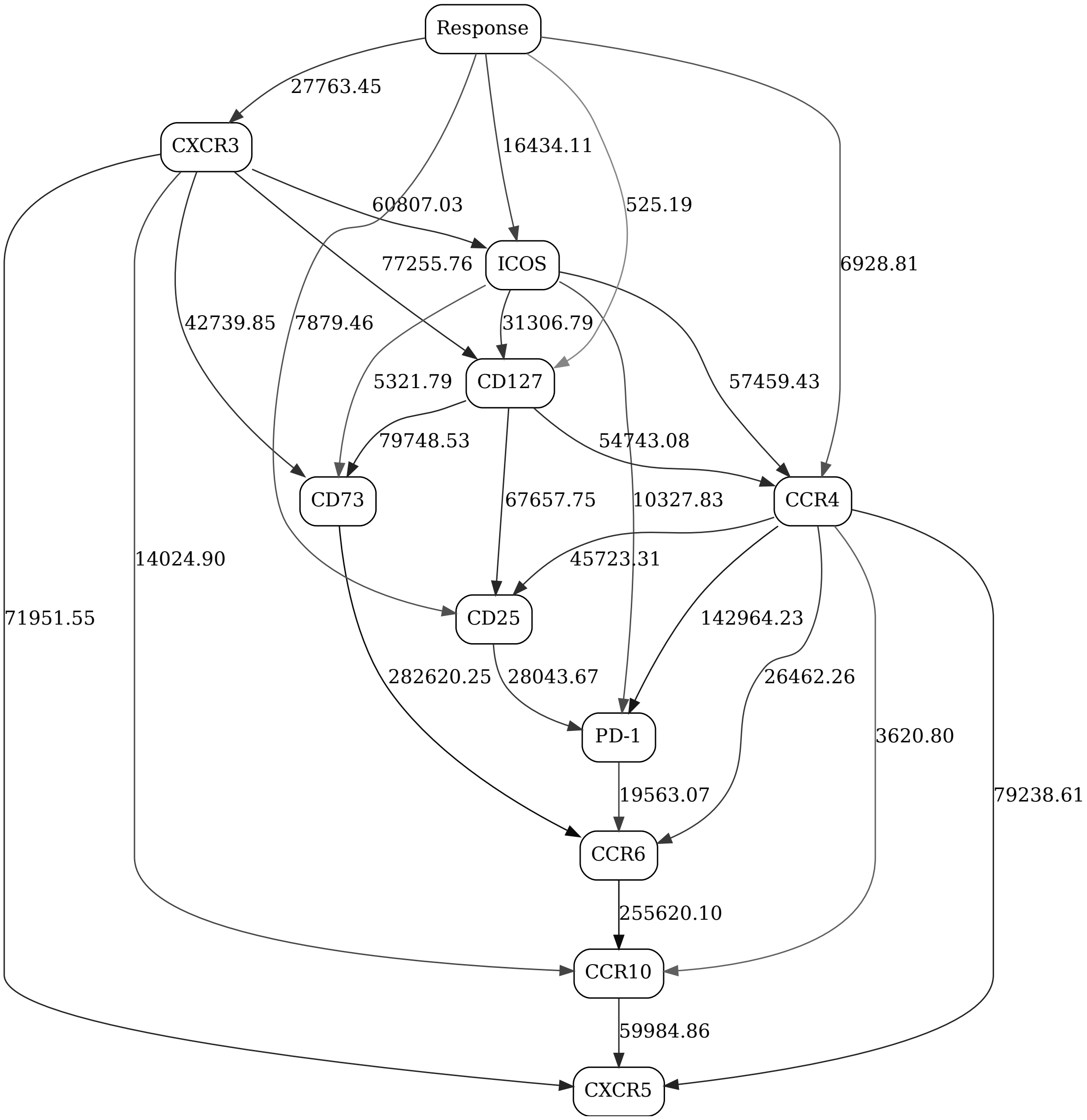

Figure 34: Adaptive immune signaling network panel, non-naive CD4, day 1, response contrast (2-state "Response" variable)

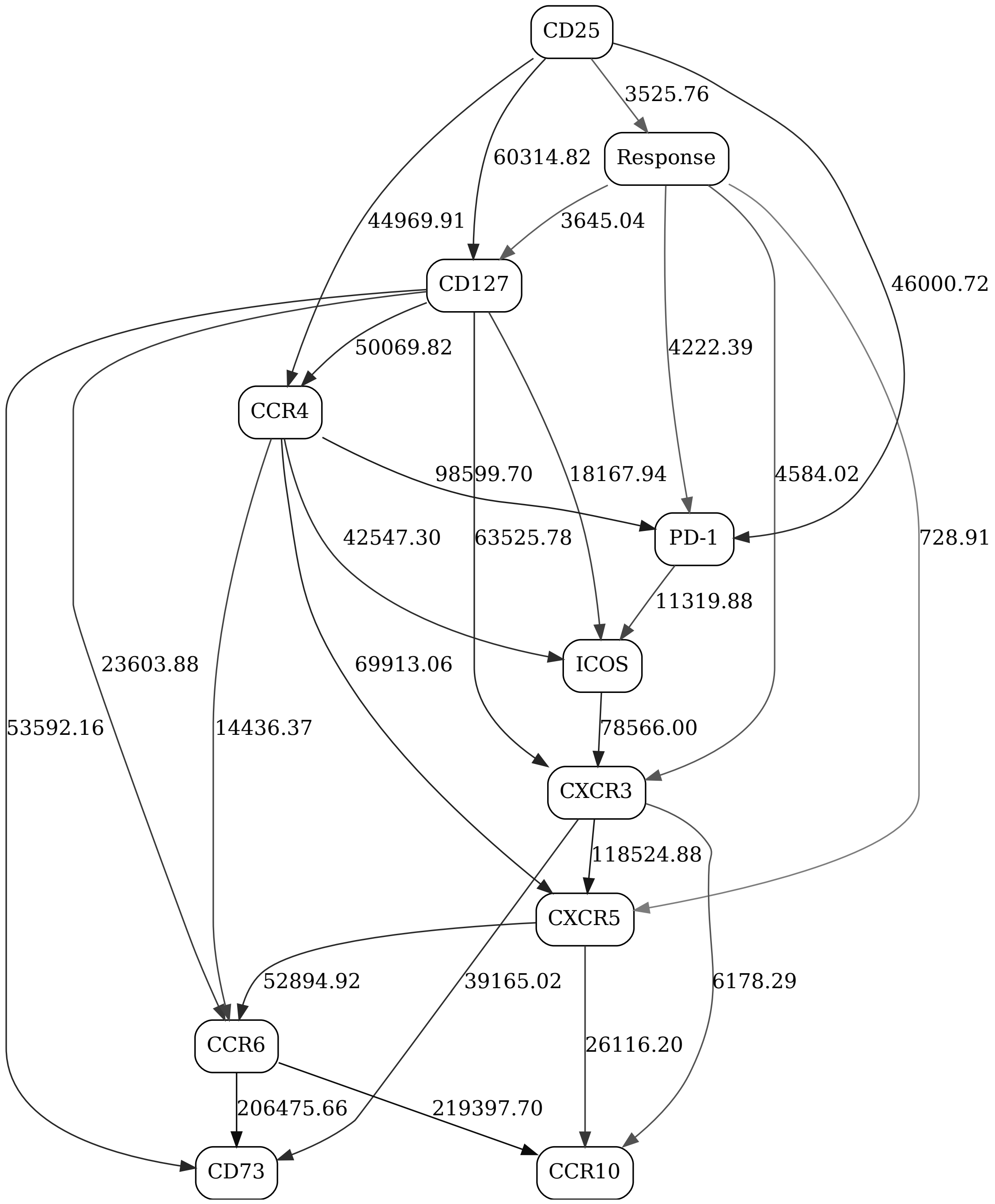

Figure 35: Adaptive immune signaling network panel, non-naive CD4, day 21, response contrast (2-state "Response" variable)

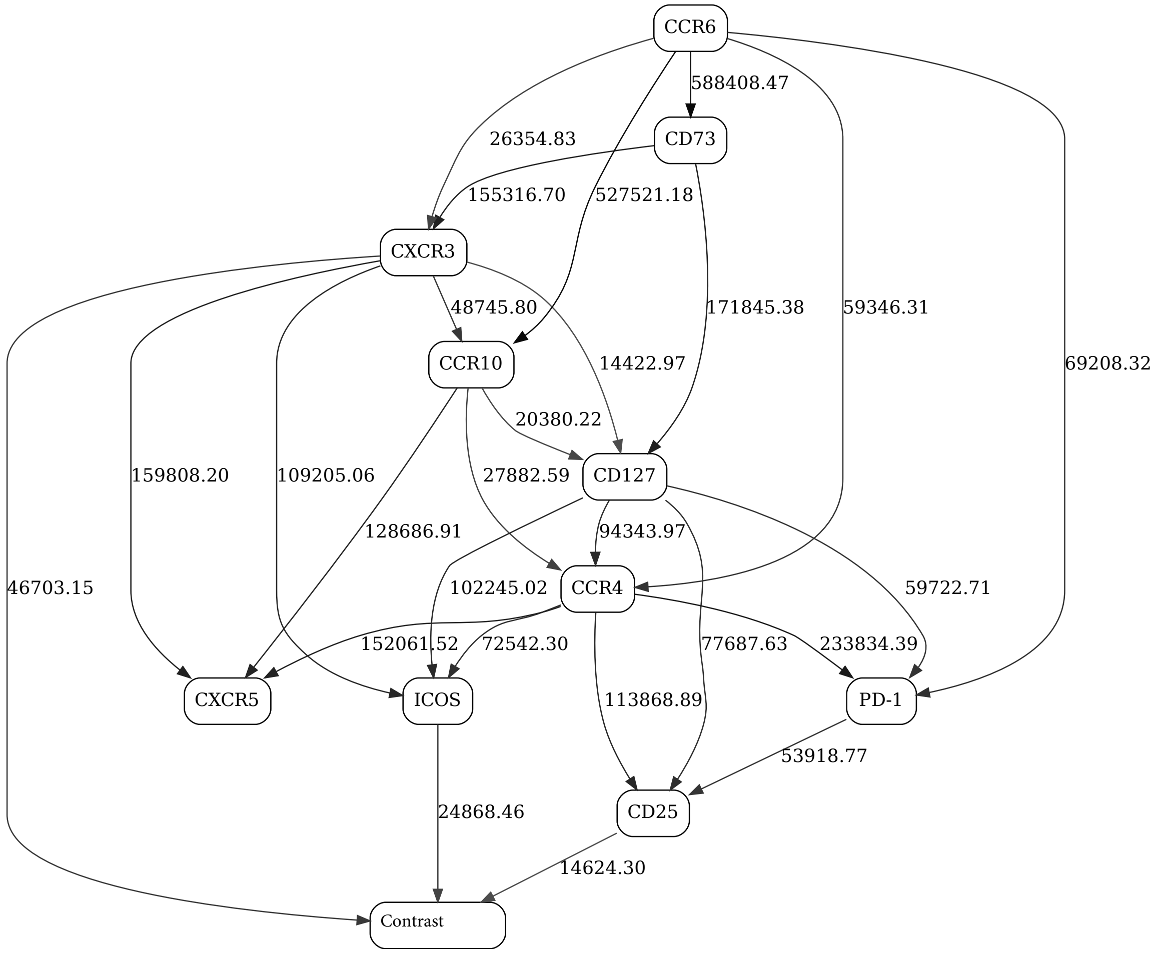

Figure 36: Adaptive immune signaling network panel, non-naive CD4

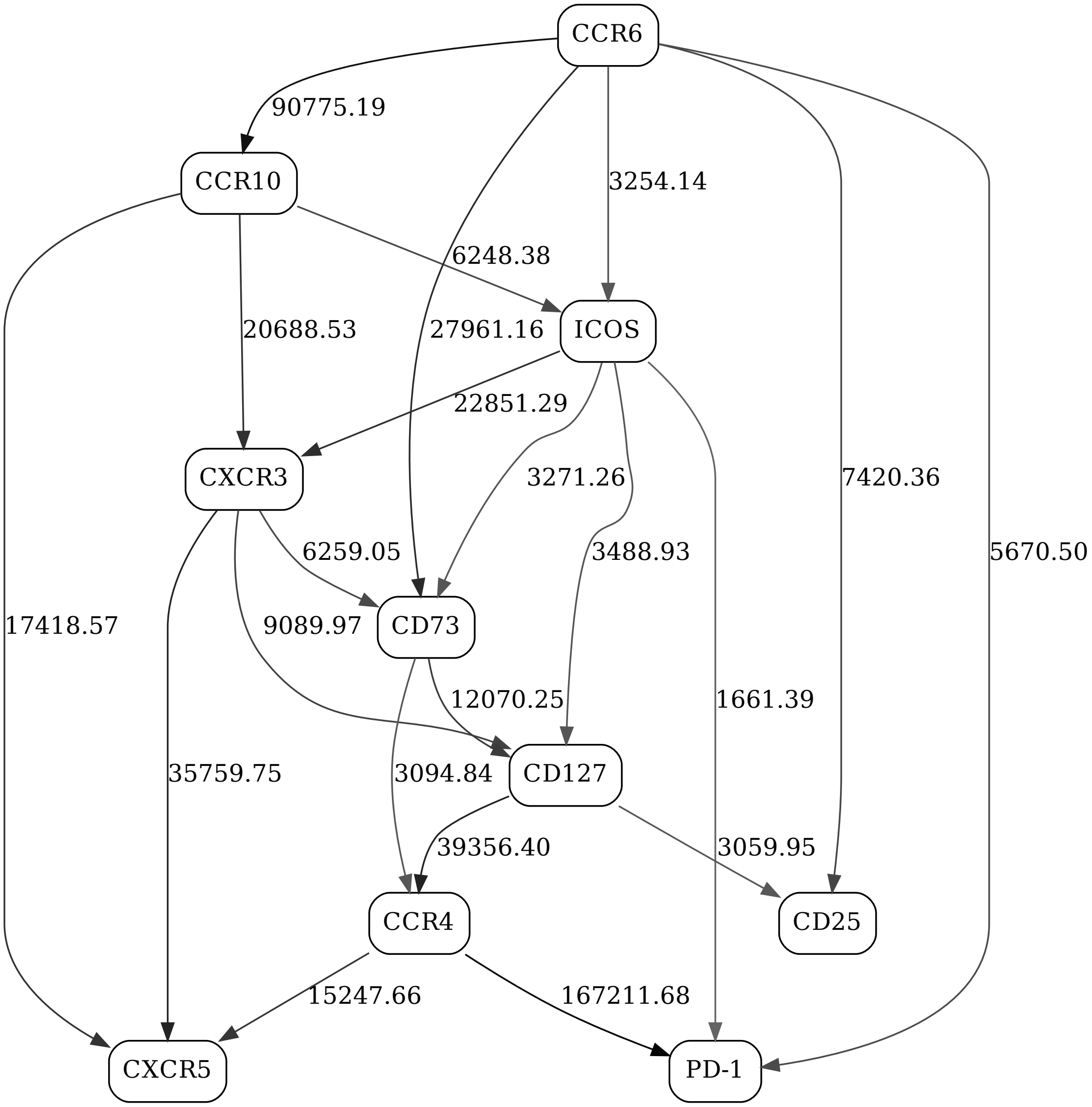

Figure 37: Adaptive immune signaling network panel, naive CD4, day 1, responders

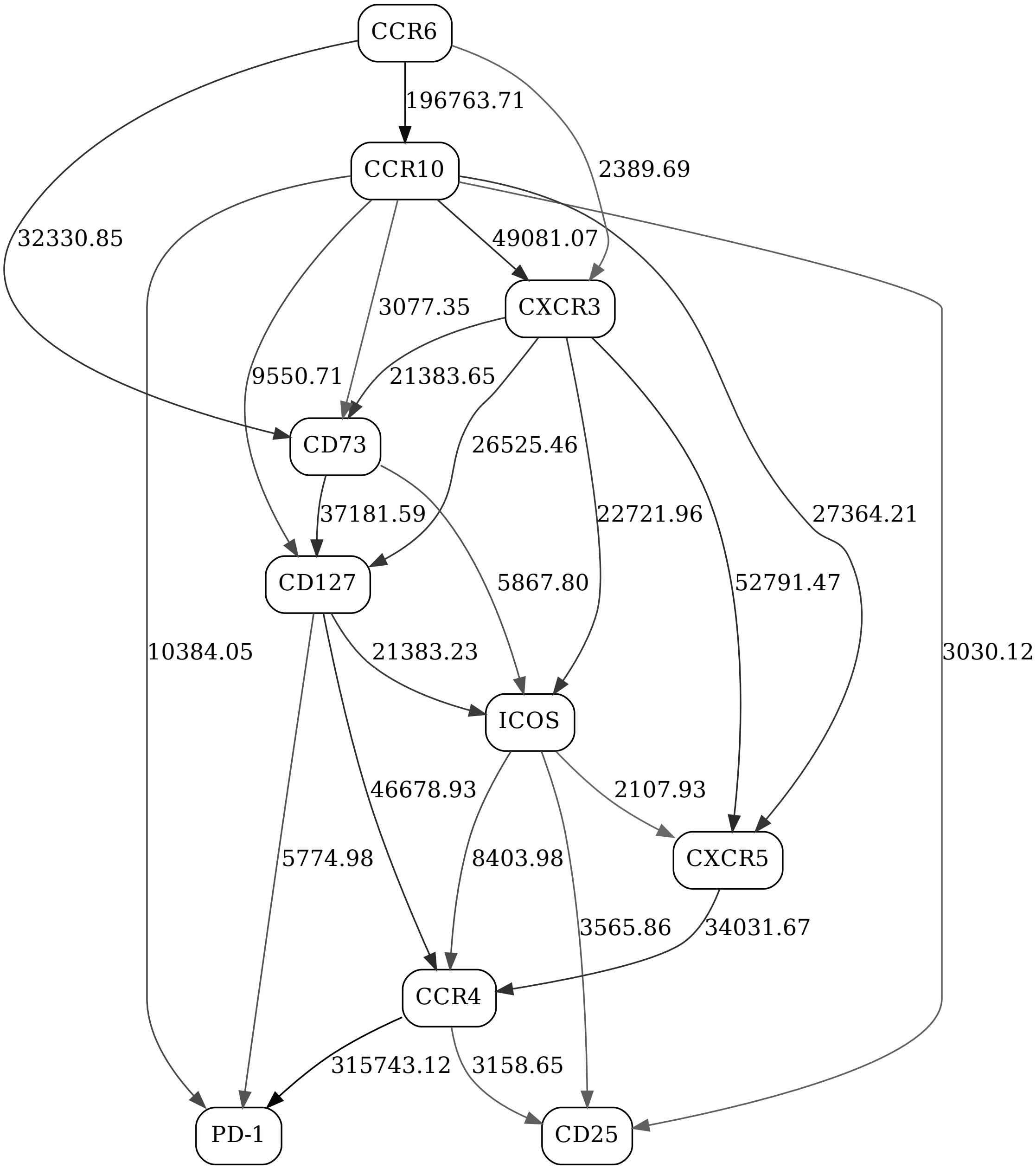

Figure 38: Adaptive immune signaling network panel, naive CD4, day 1, non-responders

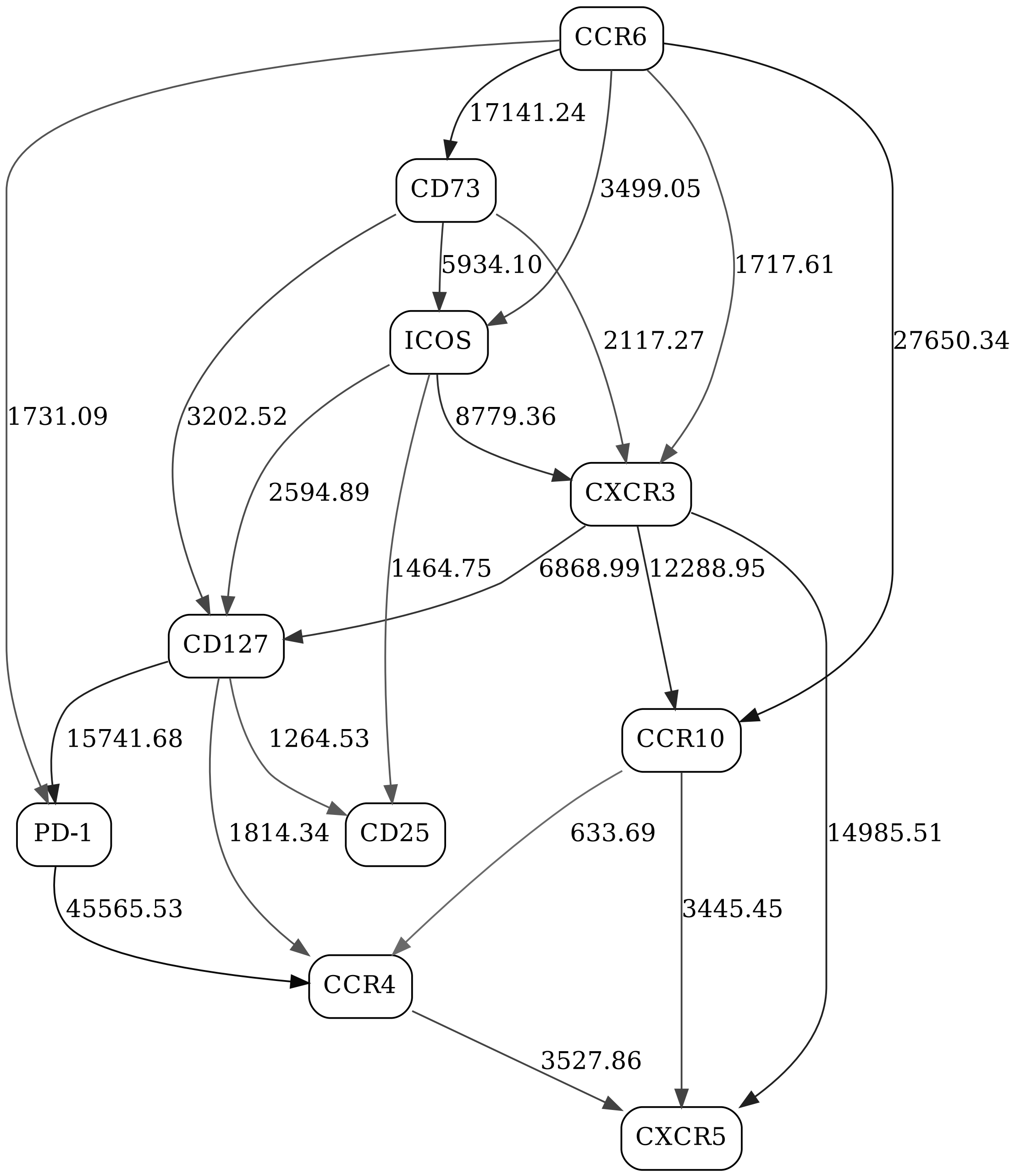

Figure 39: Adaptive immune signaling network panel, naive CD4, day 21, responders

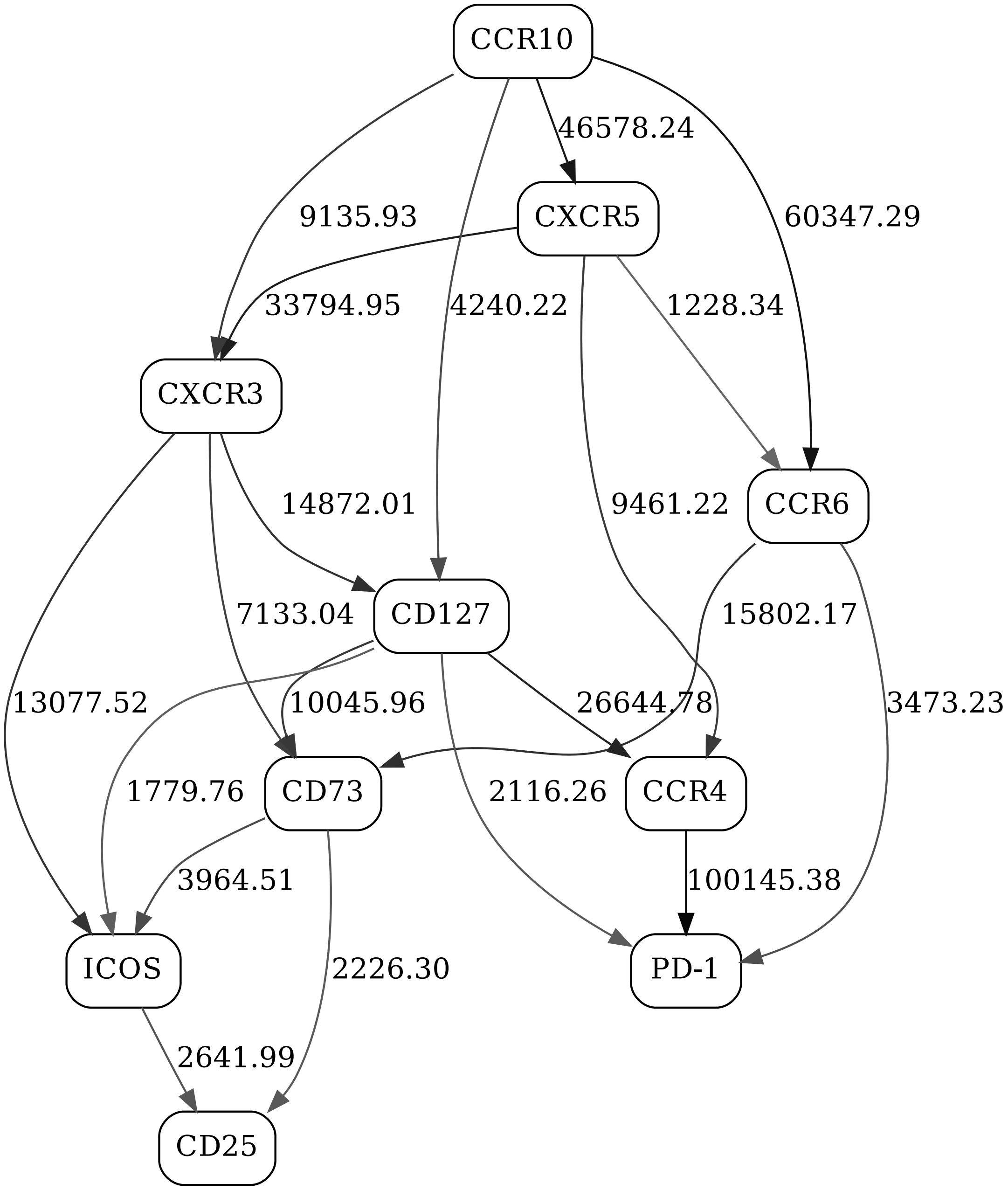

Figure 40: Adaptive immune signaling network panel, naive CD4, day 21, non-responders

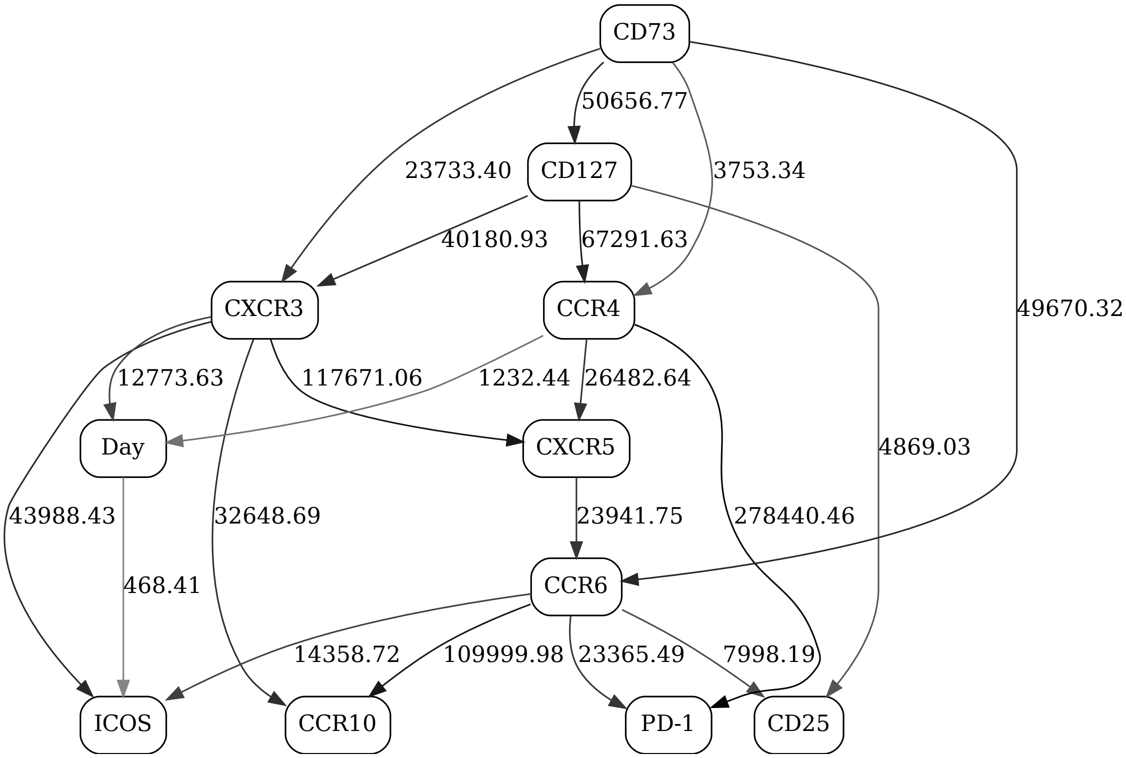

Figure 41: Adaptive immune signaling network panel, naive CD4, day contrast (2-state "Day" variable), responders

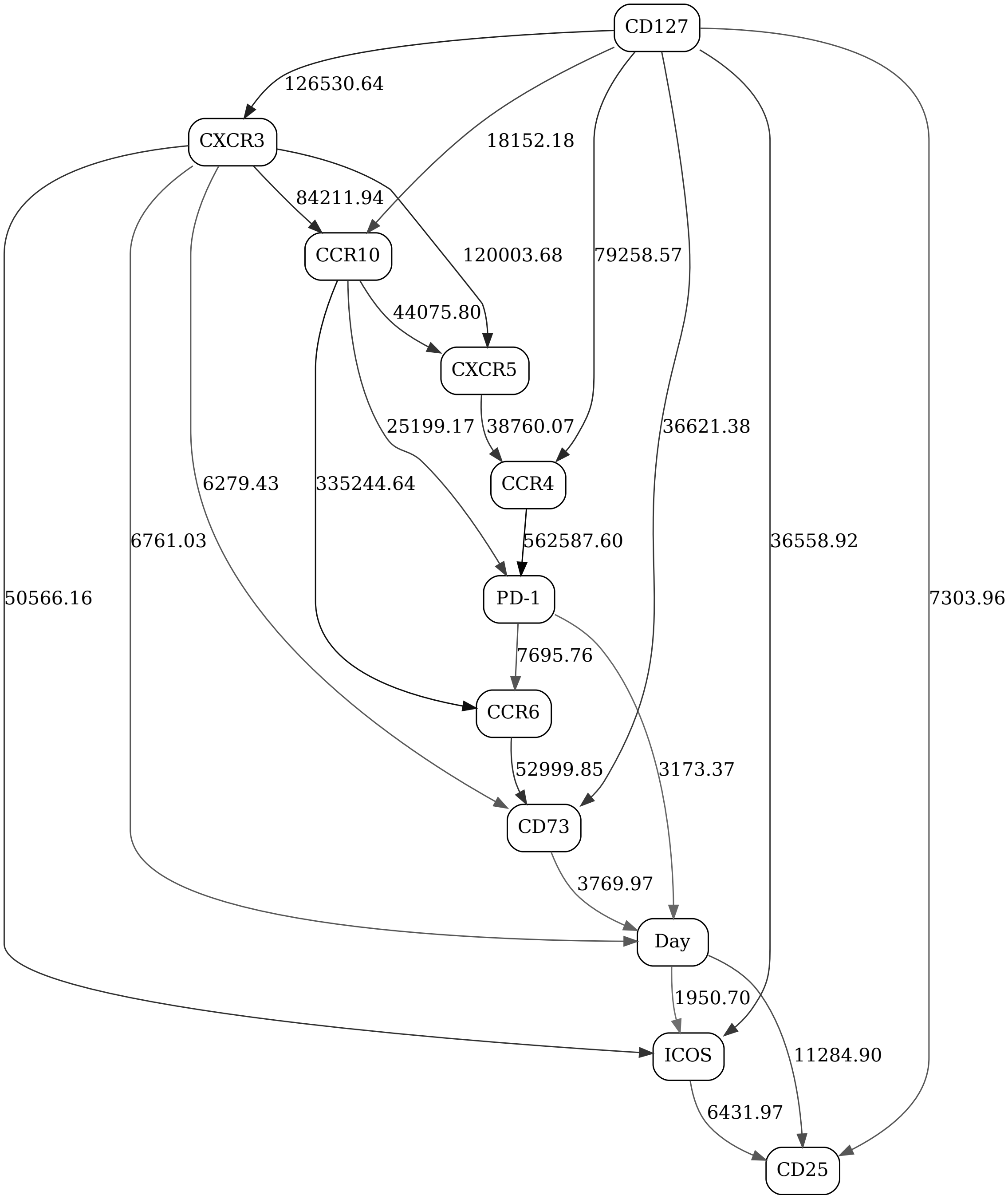

Figure 42: Adaptive immune signaling network panel, naive CD4, day contrast (2-state "Day" variable), non-responders

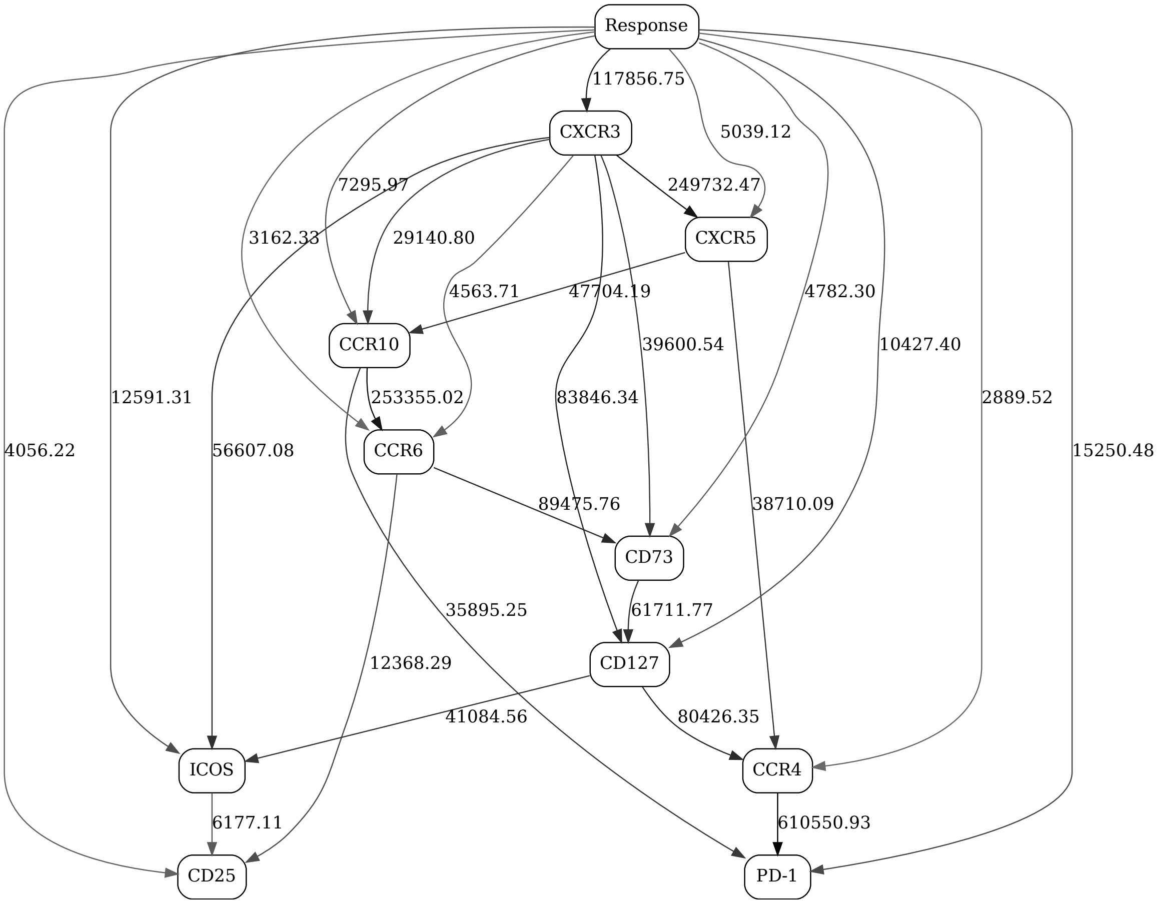

Figure 43: Adaptive immune signaling network panel, naive CD4, day 1, response contrast (2-state "Response" variable)

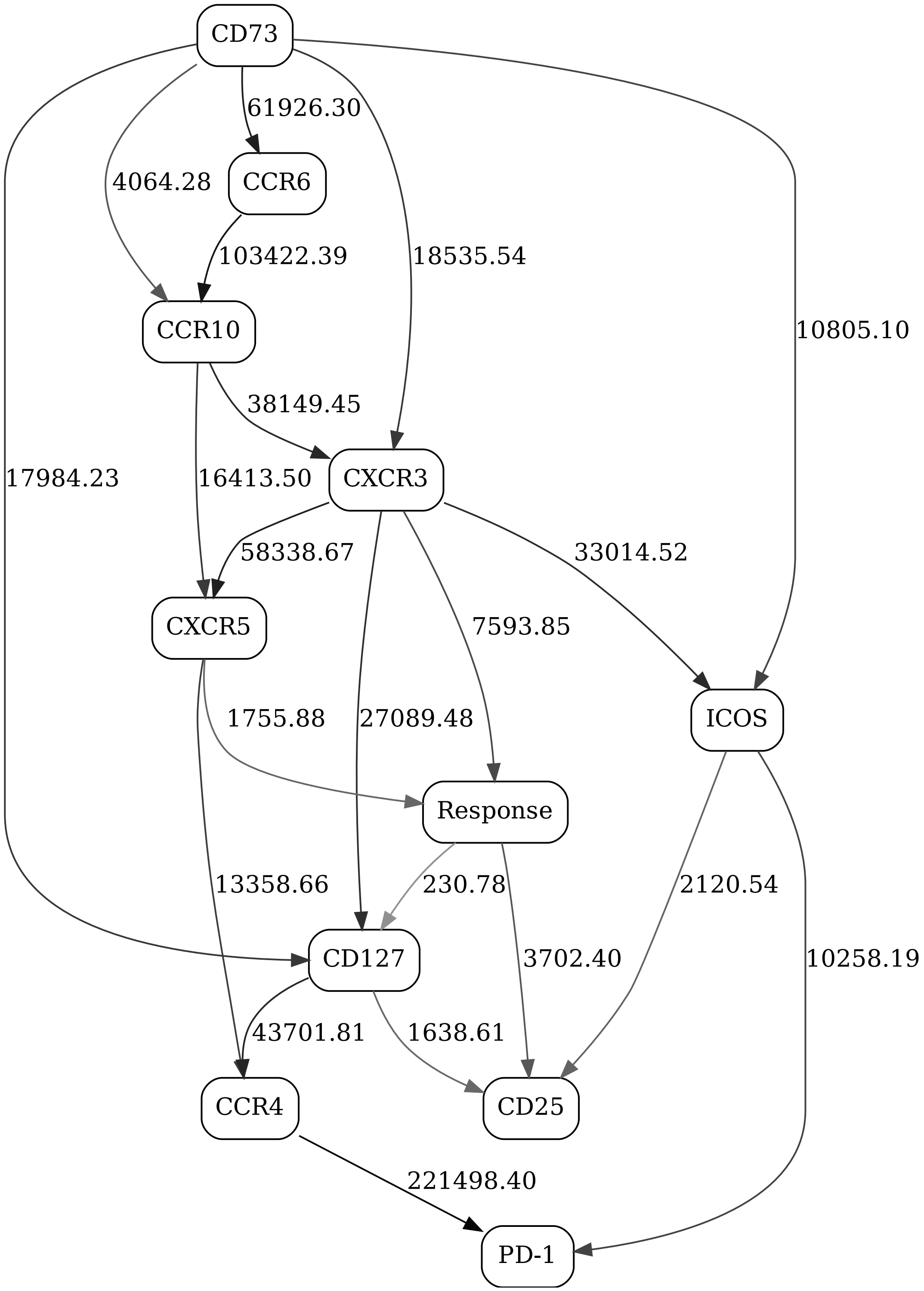

Figure 44: Adaptive immune signaling network panel, naive CD4, day 21, response contrast (2-state "Response" variable)

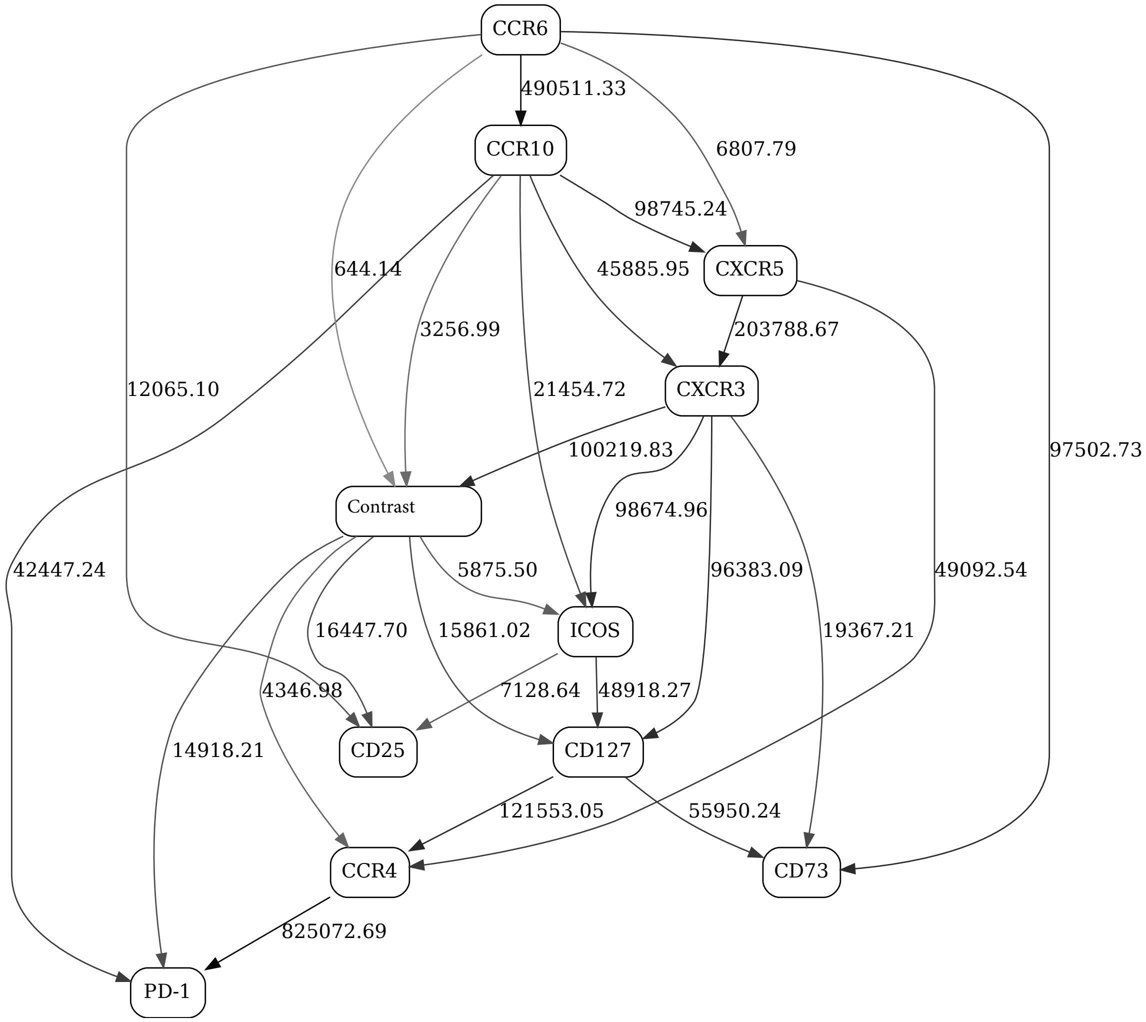

Figure 45: Adaptive immune signaling network panel, naive CD4

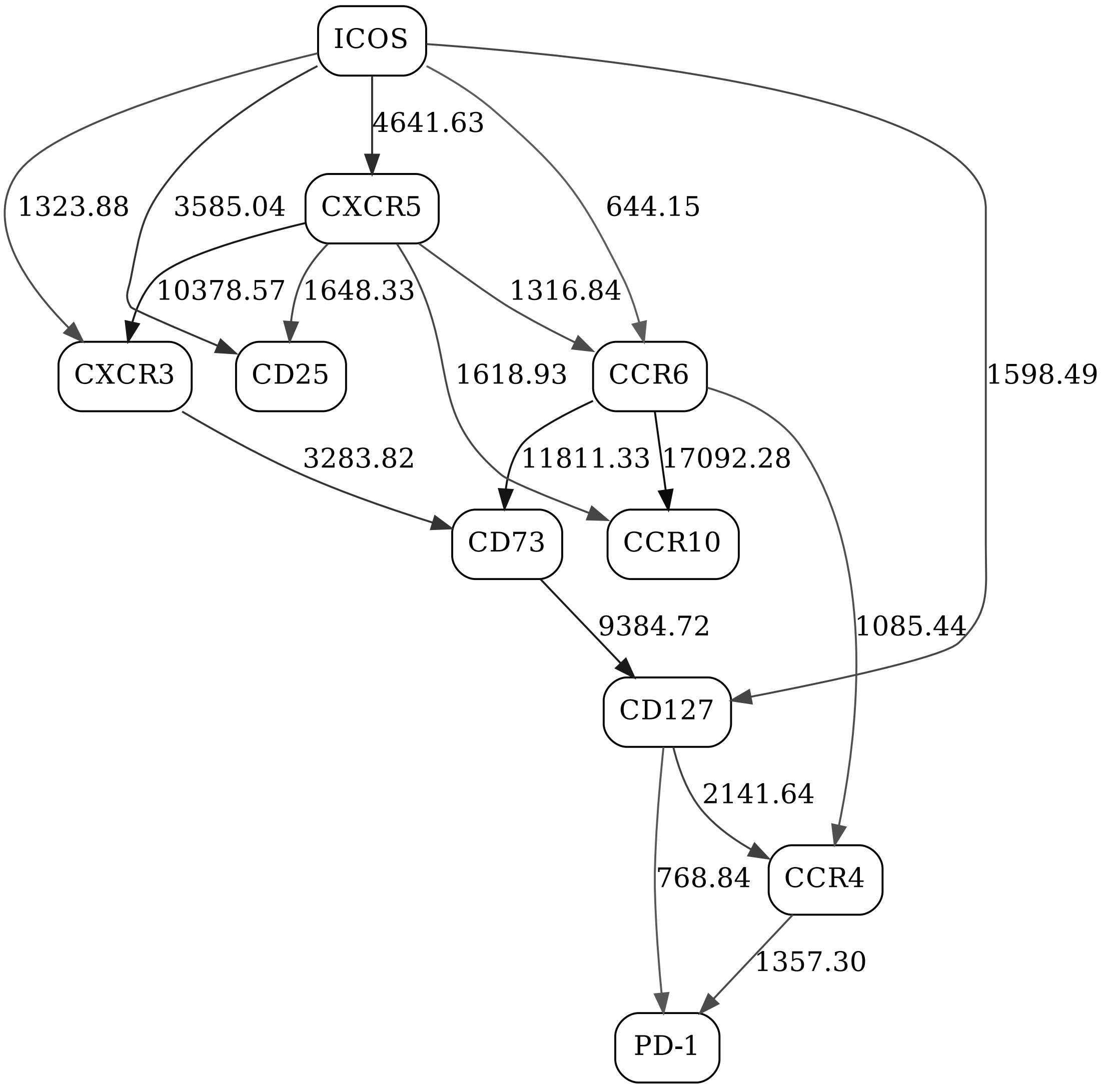

Figure 46: Adaptive immune signaling network panel, non-naive CD8, day 1, responders

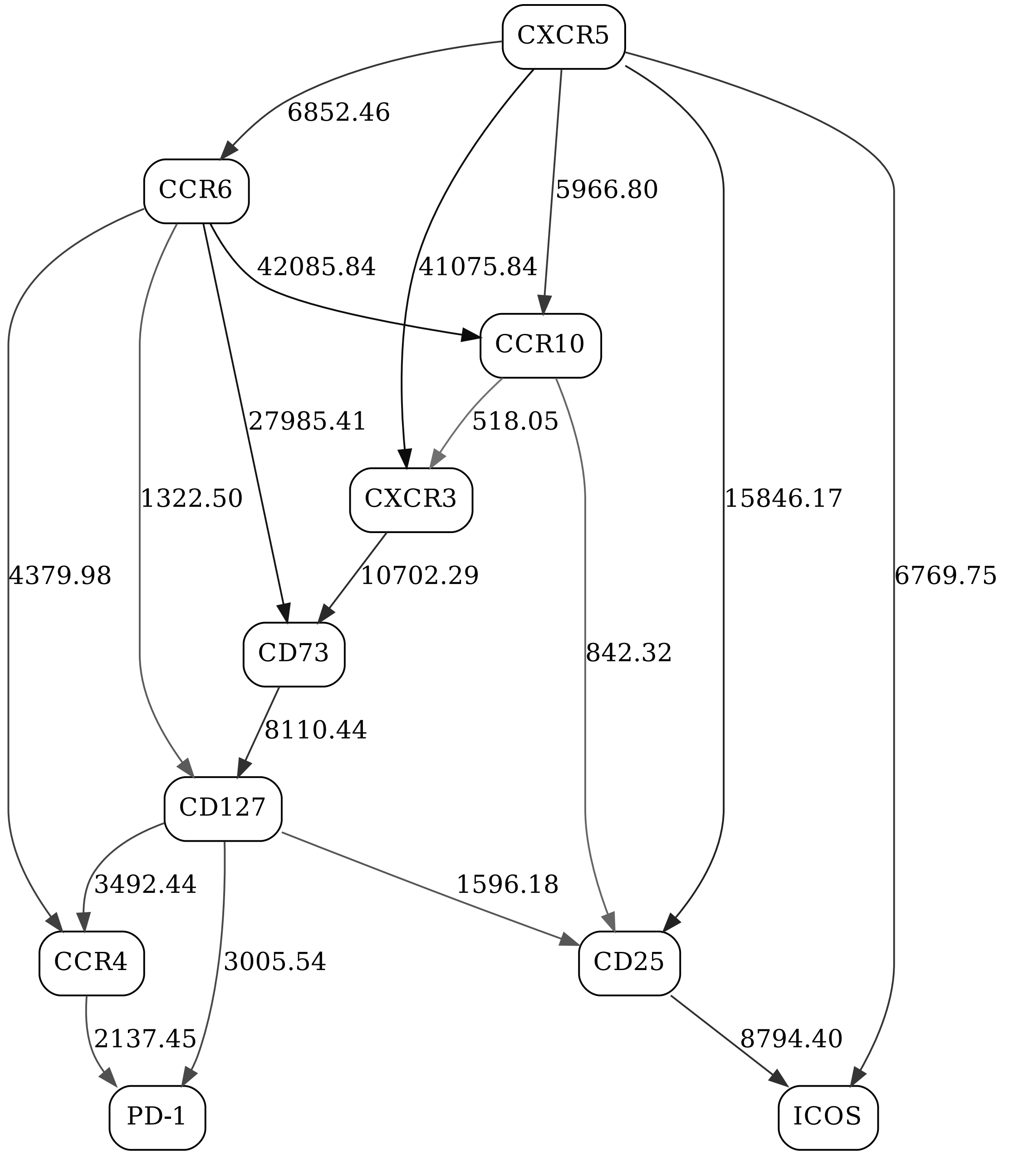

Figure 47: Adaptive immune signaling network panel, non-naive CD8, day 1, non-responders

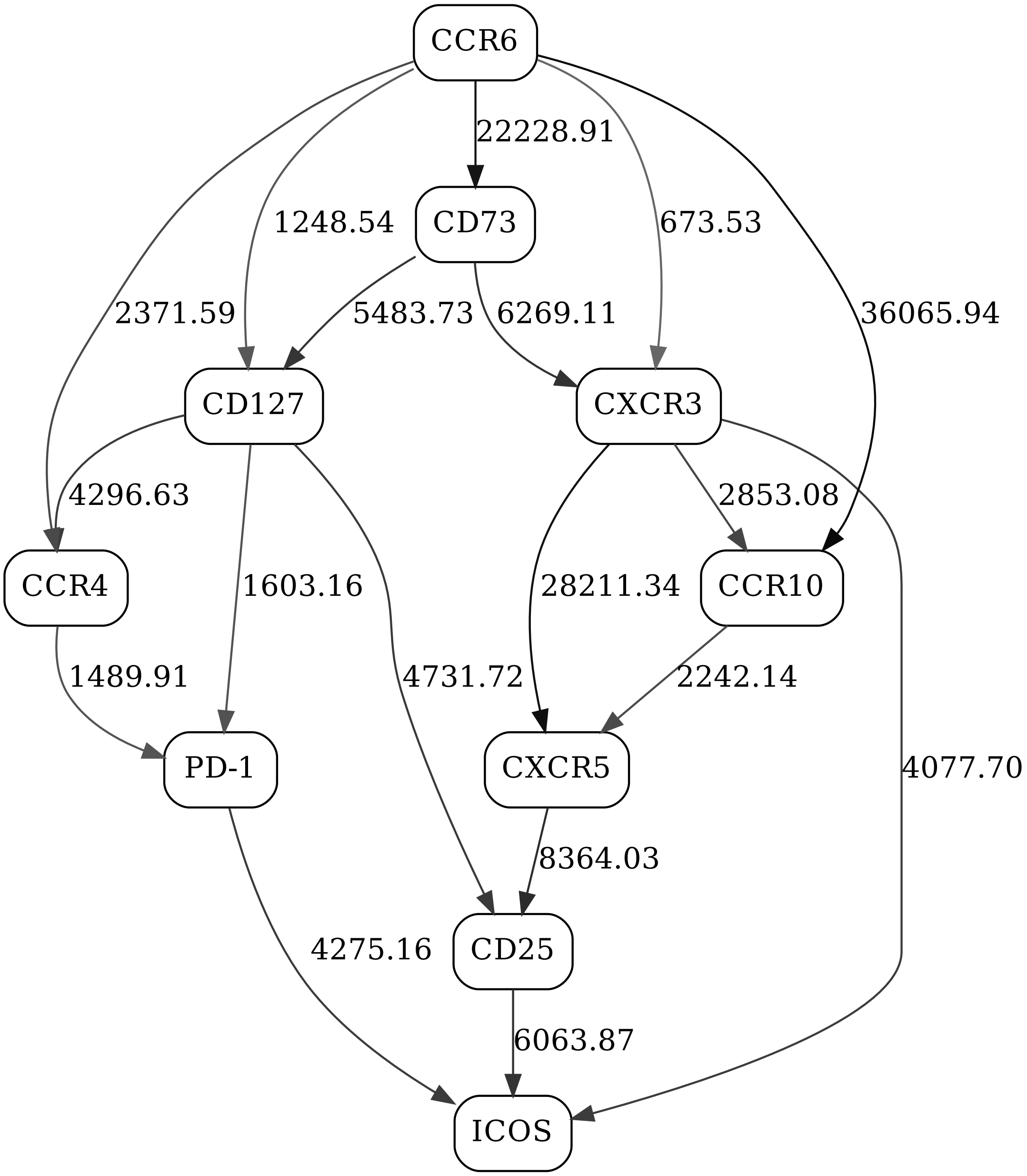

Figure 48: Adaptive immune signaling network panel, non-naive CD8, day 21, responders

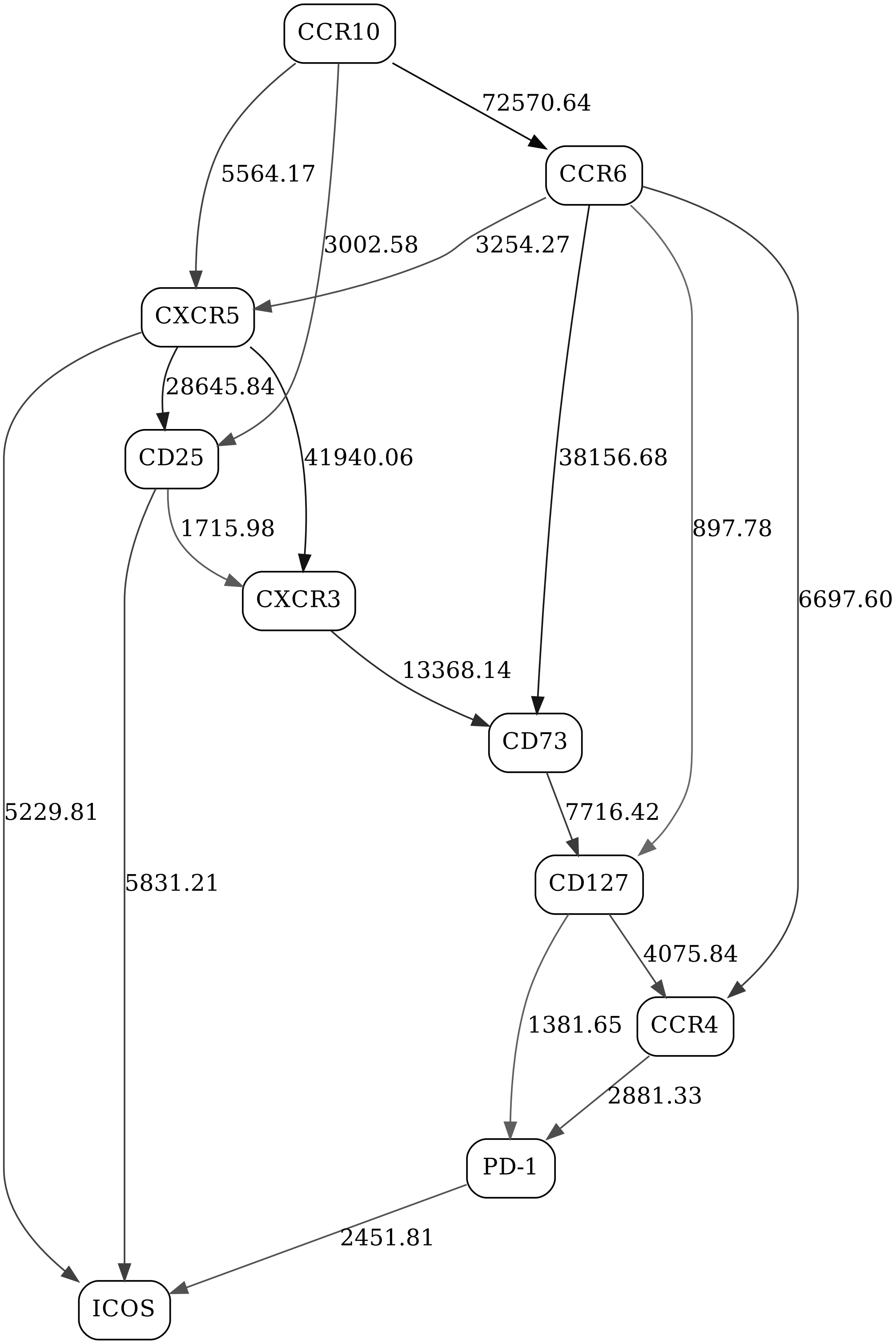

Figure 49: Adaptive immune signaling network panel, non-naive CD8, day 21, non-responders

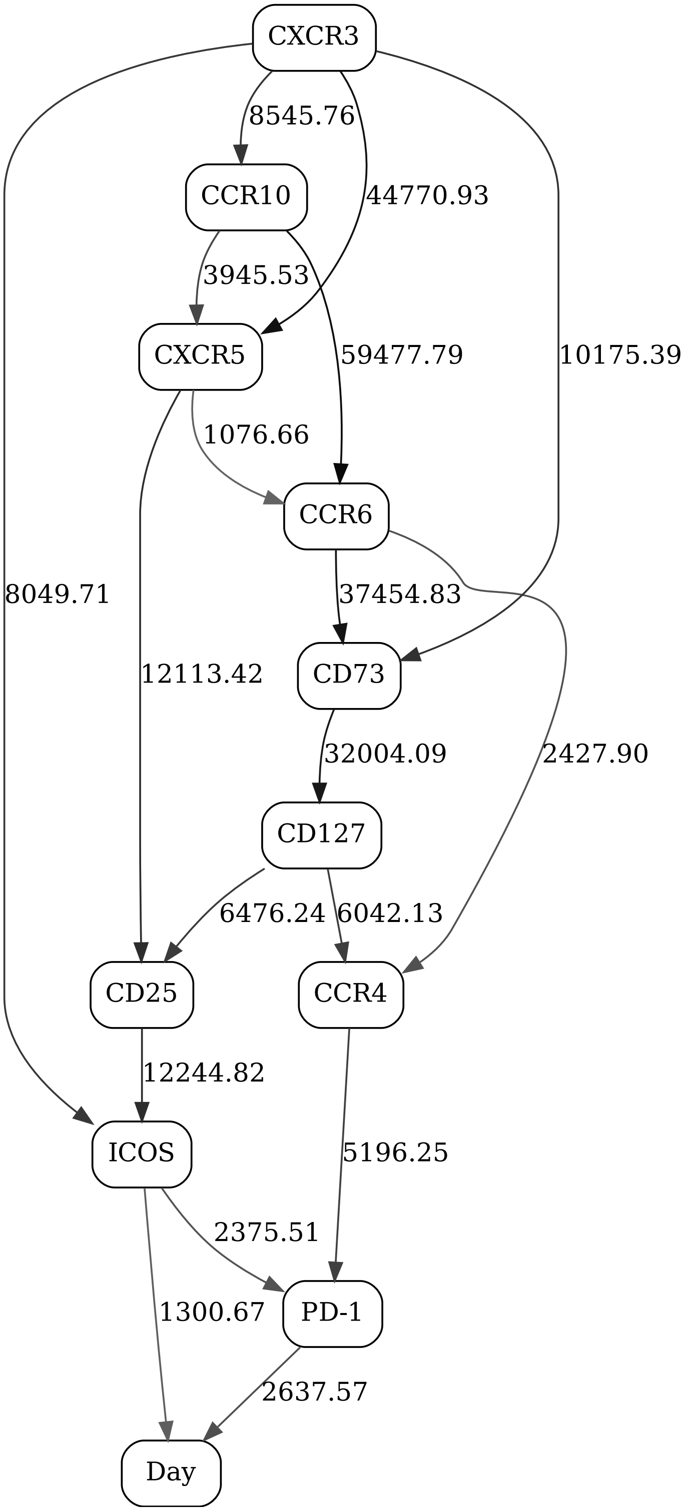

Figure 50: Adaptive immune signaling network panel, non-naive CD8, day contrast (2-state "Day" variable), responders

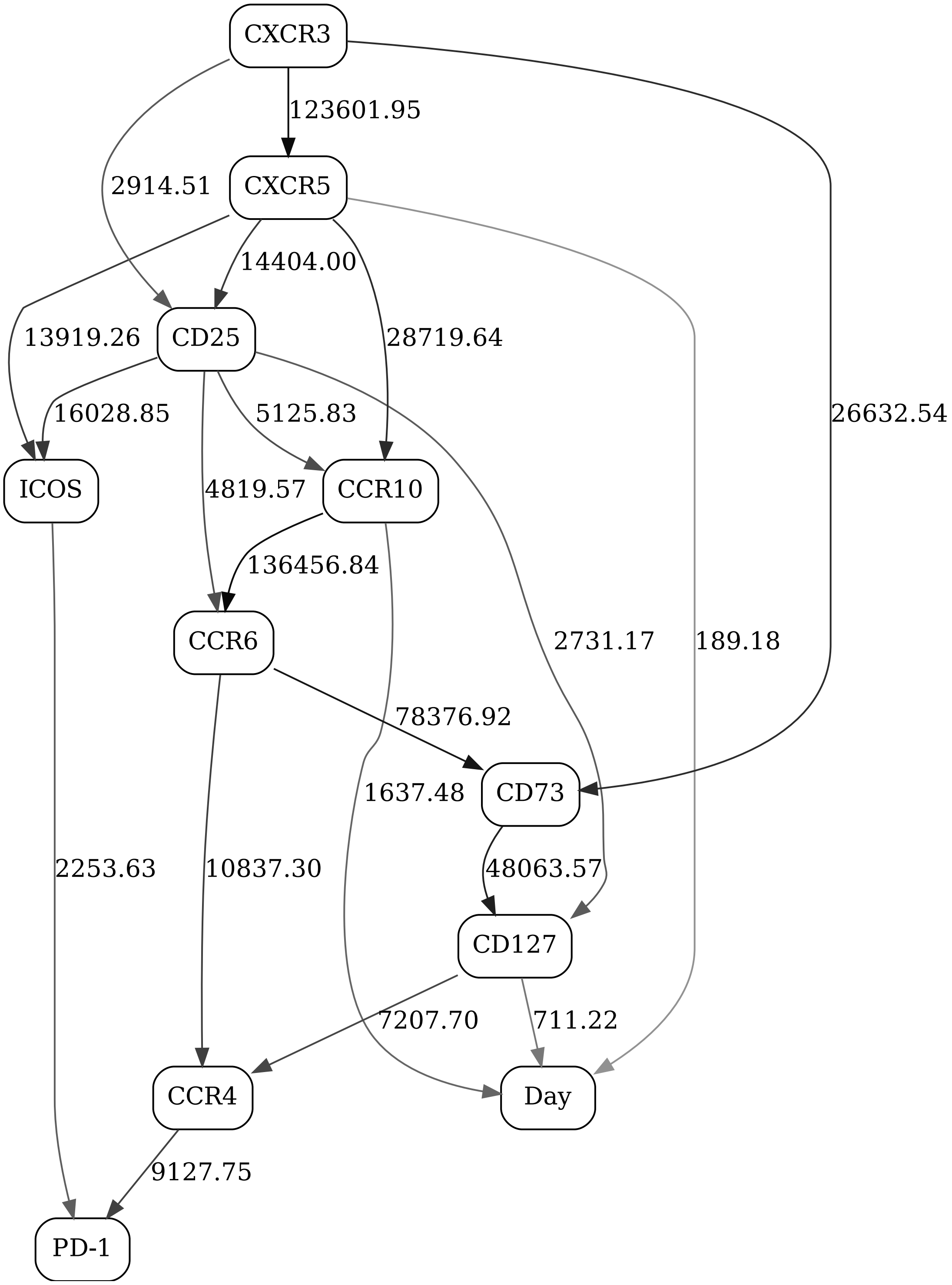

Figure 51: Adaptive immune signaling network panel, non-naive CD8, day contrast (2-state "Day" variable), non-responders

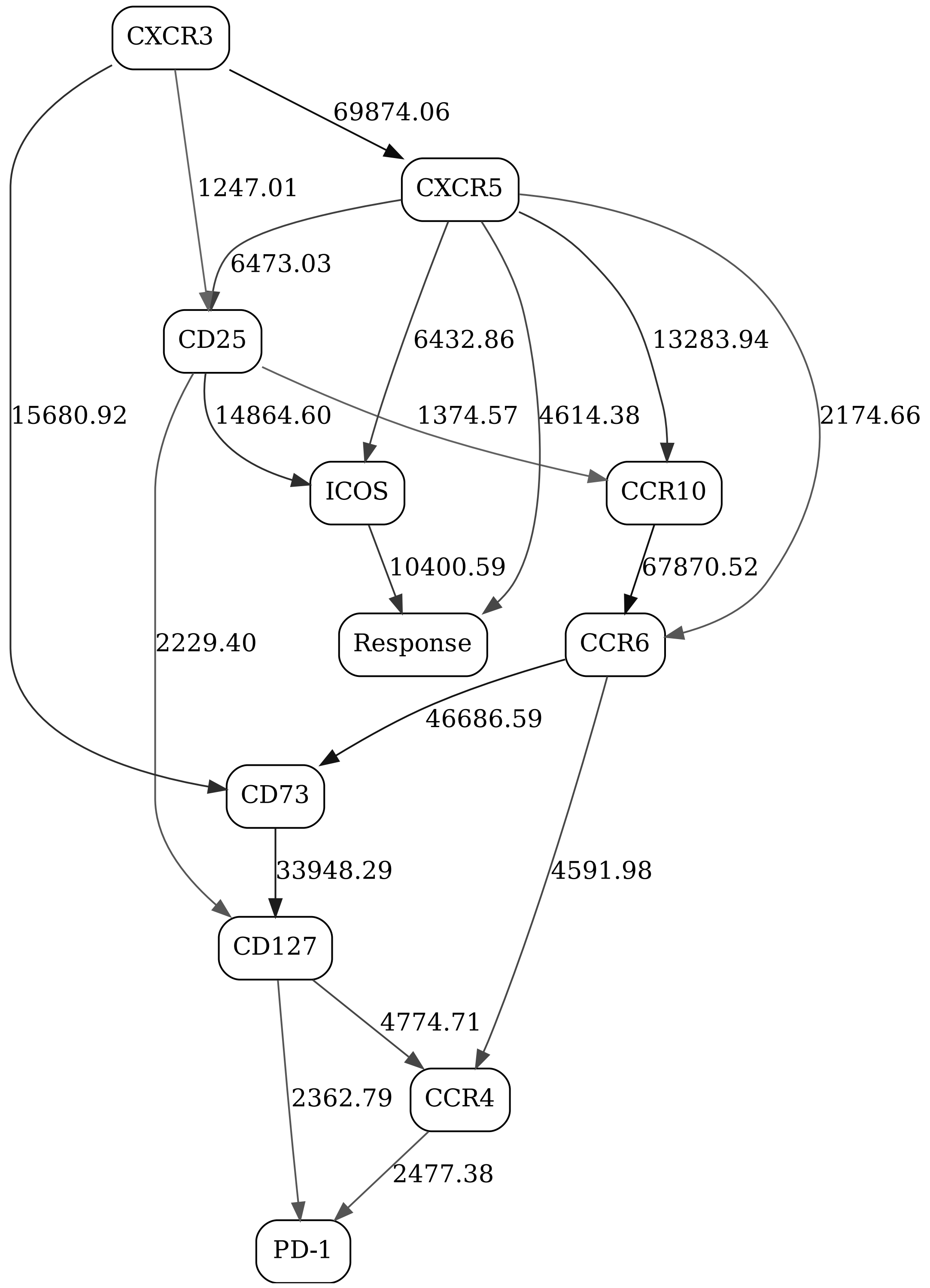

Figure 52: Adaptive immune signaling network panel, non-naive CD8, day 1, response contrast (2-state "Response" variable)

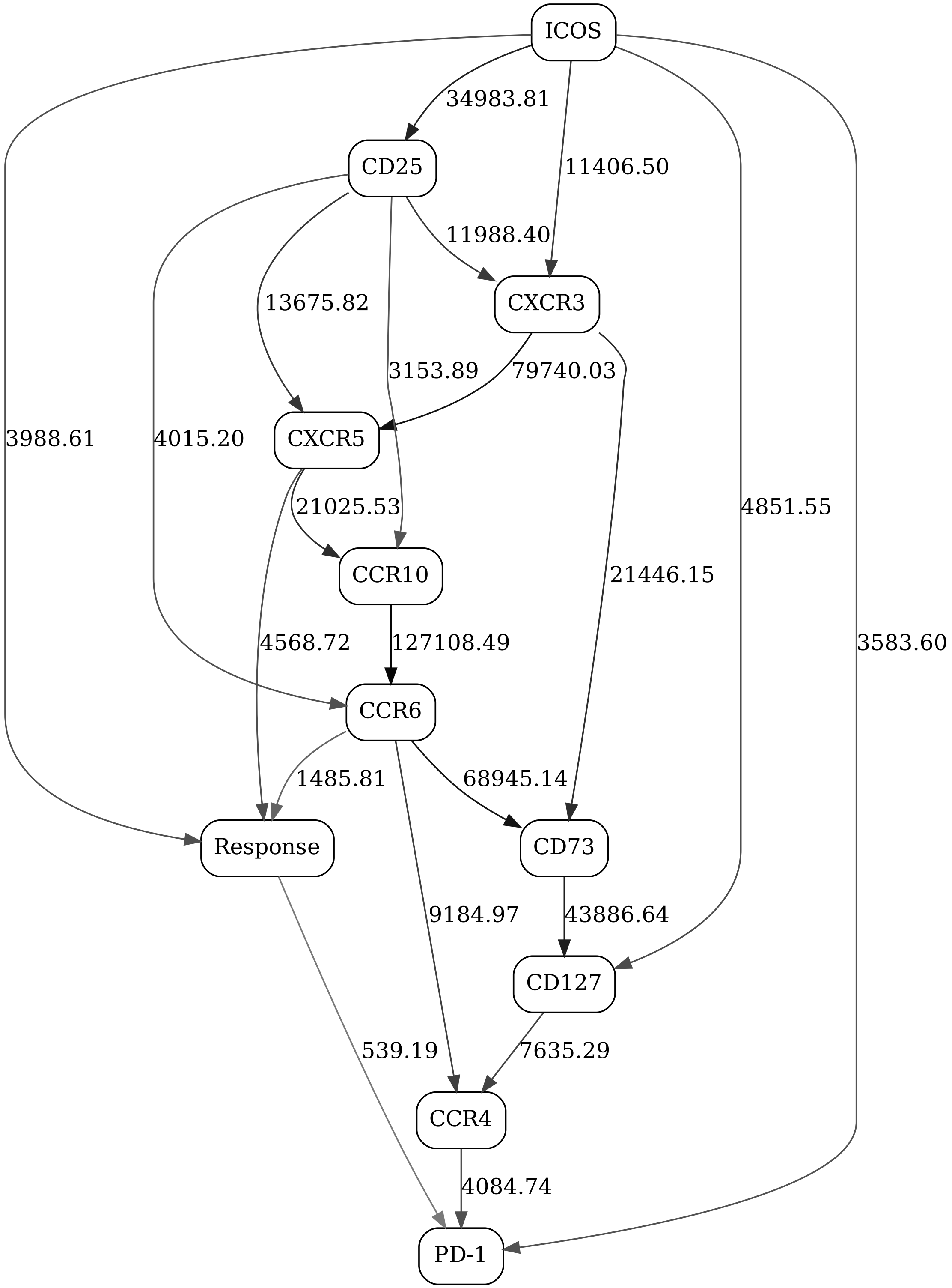

Figure 53: Adaptive immune signaling network panel, non-naive CD8, day 21, response contrast (2-state "Response" variable)

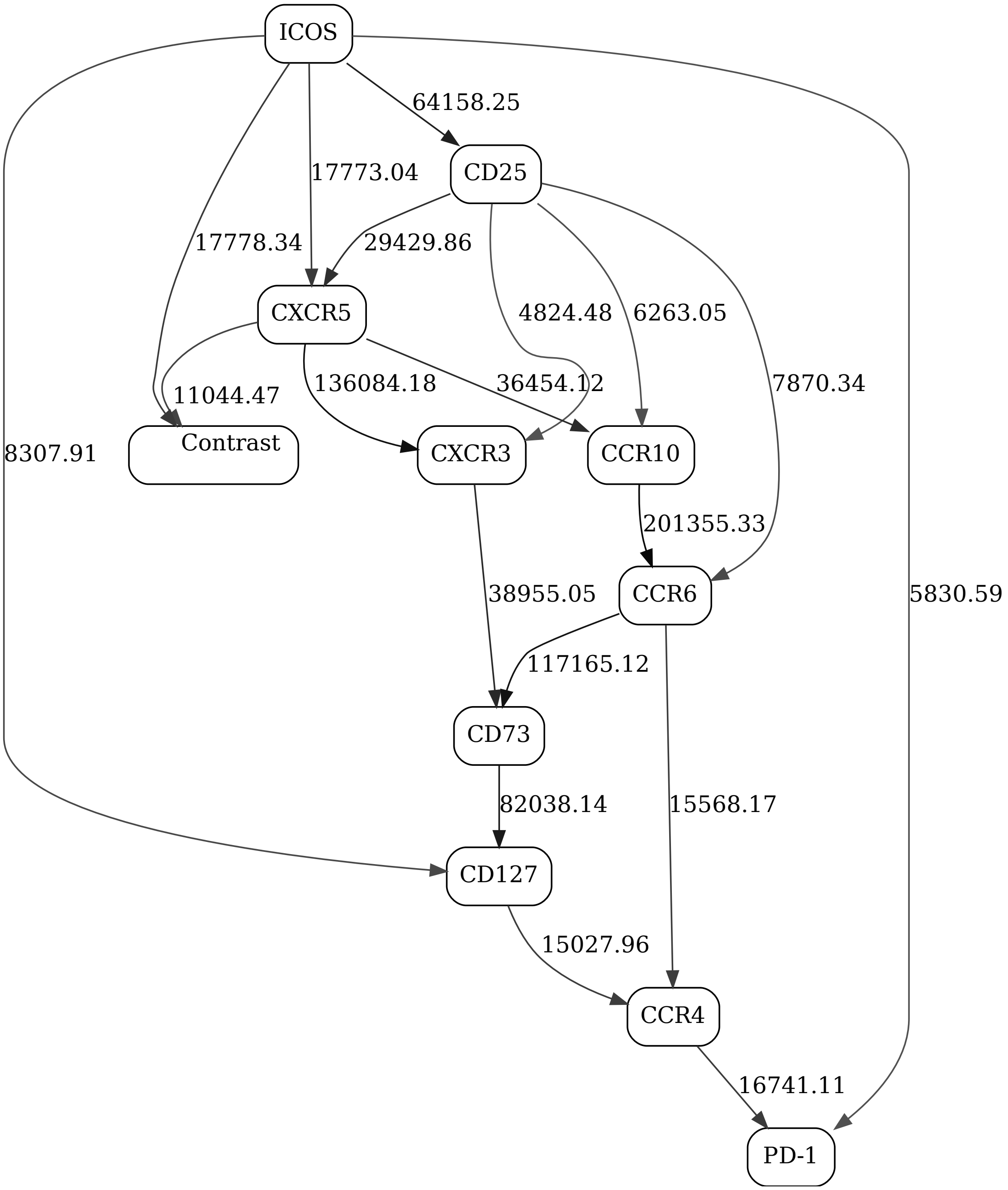

Figure 54: Adaptive immune signaling network panel, non-naive CD8

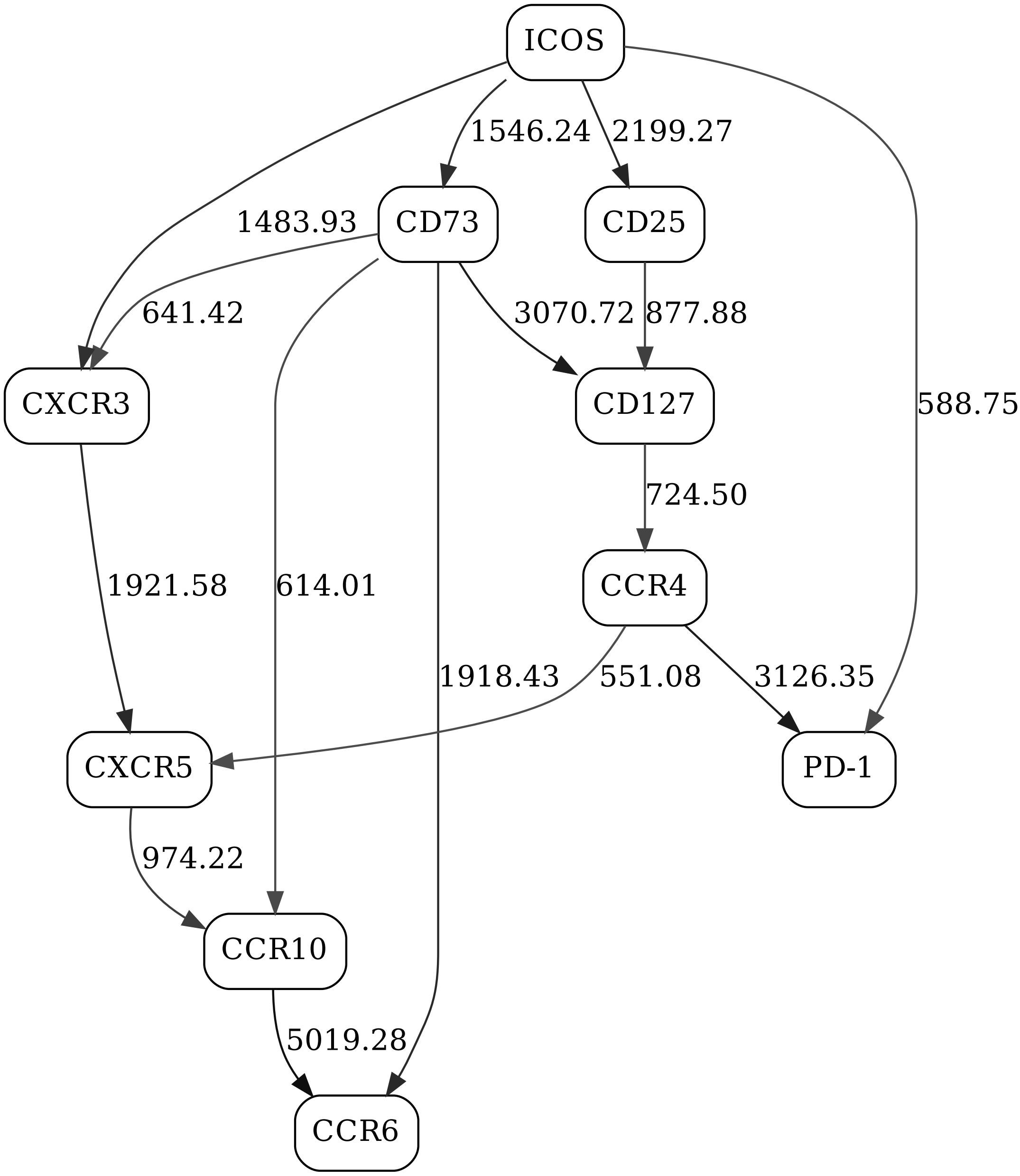

Figure 55: Adaptive immune signaling network panel, naive CD8, day 1, responders

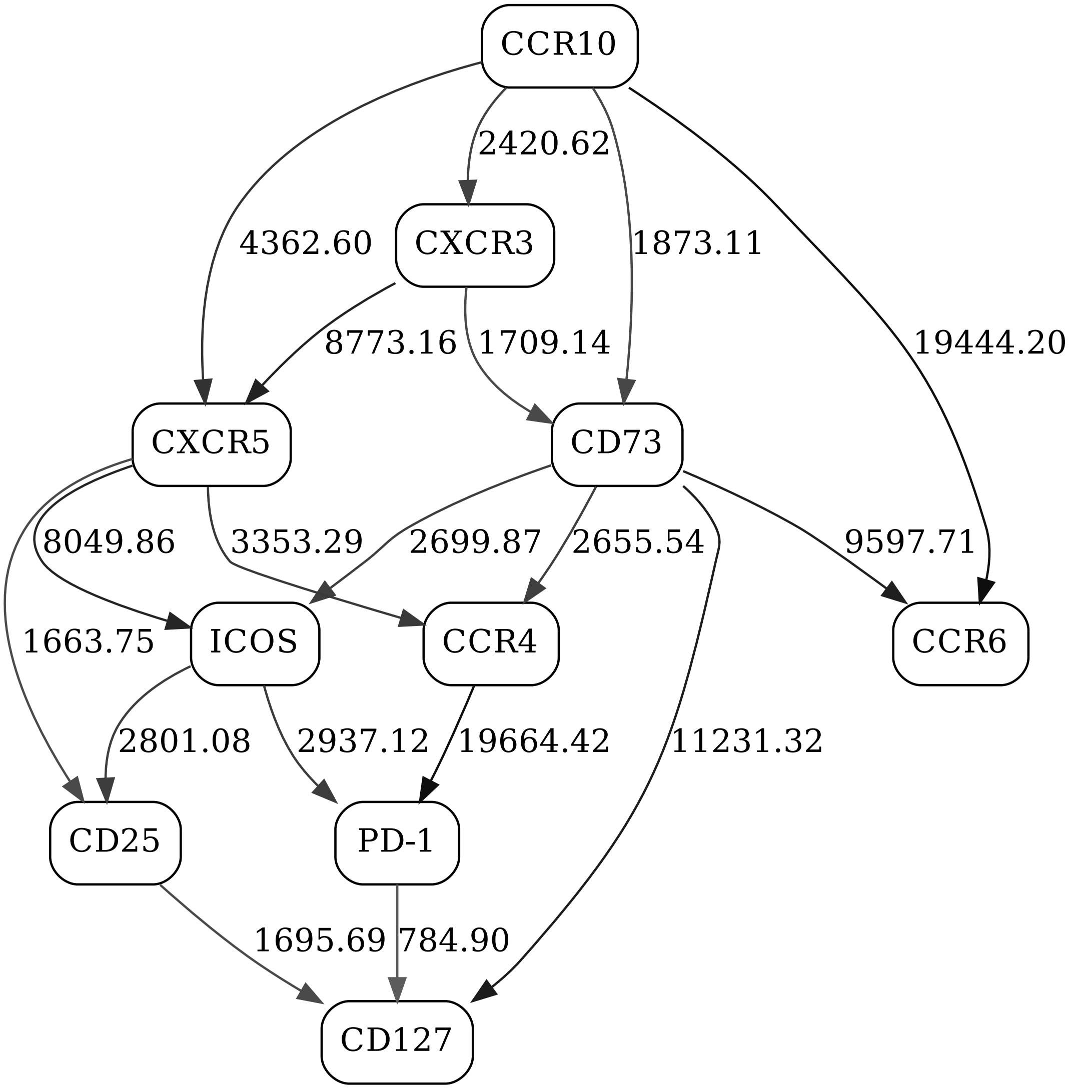

Figure 56: Adaptive immune signaling network panel, naive CD8, day 1, non-responders

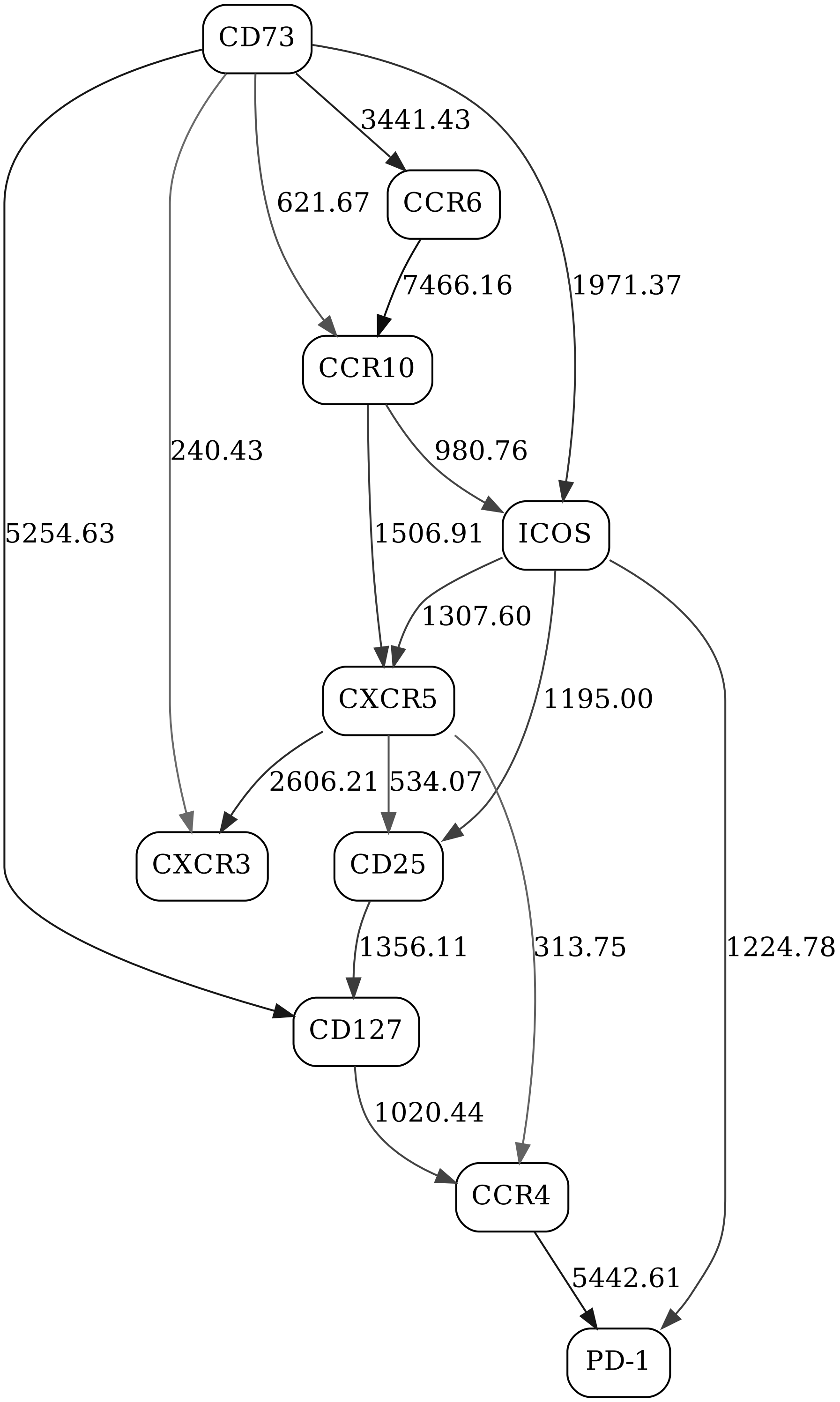

Figure 57: Adaptive immune signaling network panel, naive CD8, day 21, responders

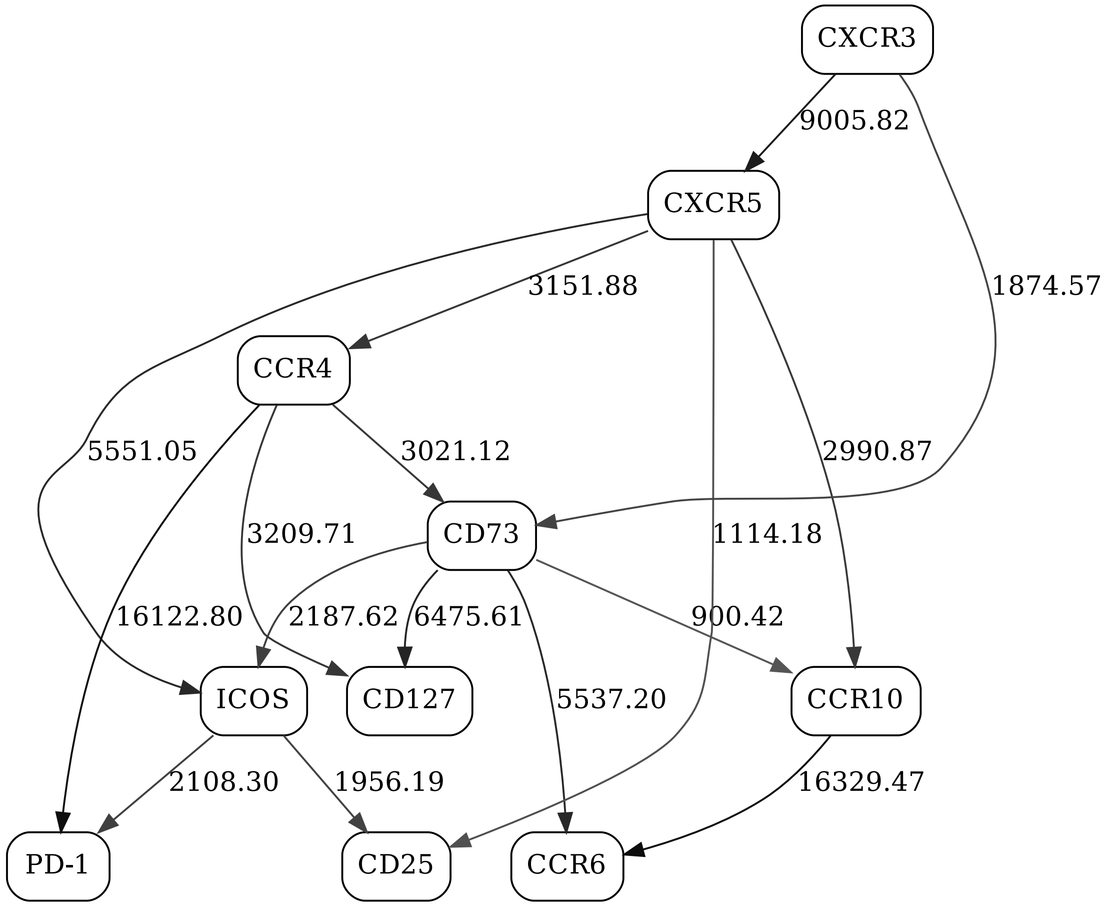

Figure 58: Adaptive immune signaling network panel, naive CD8, day 21, non-responders

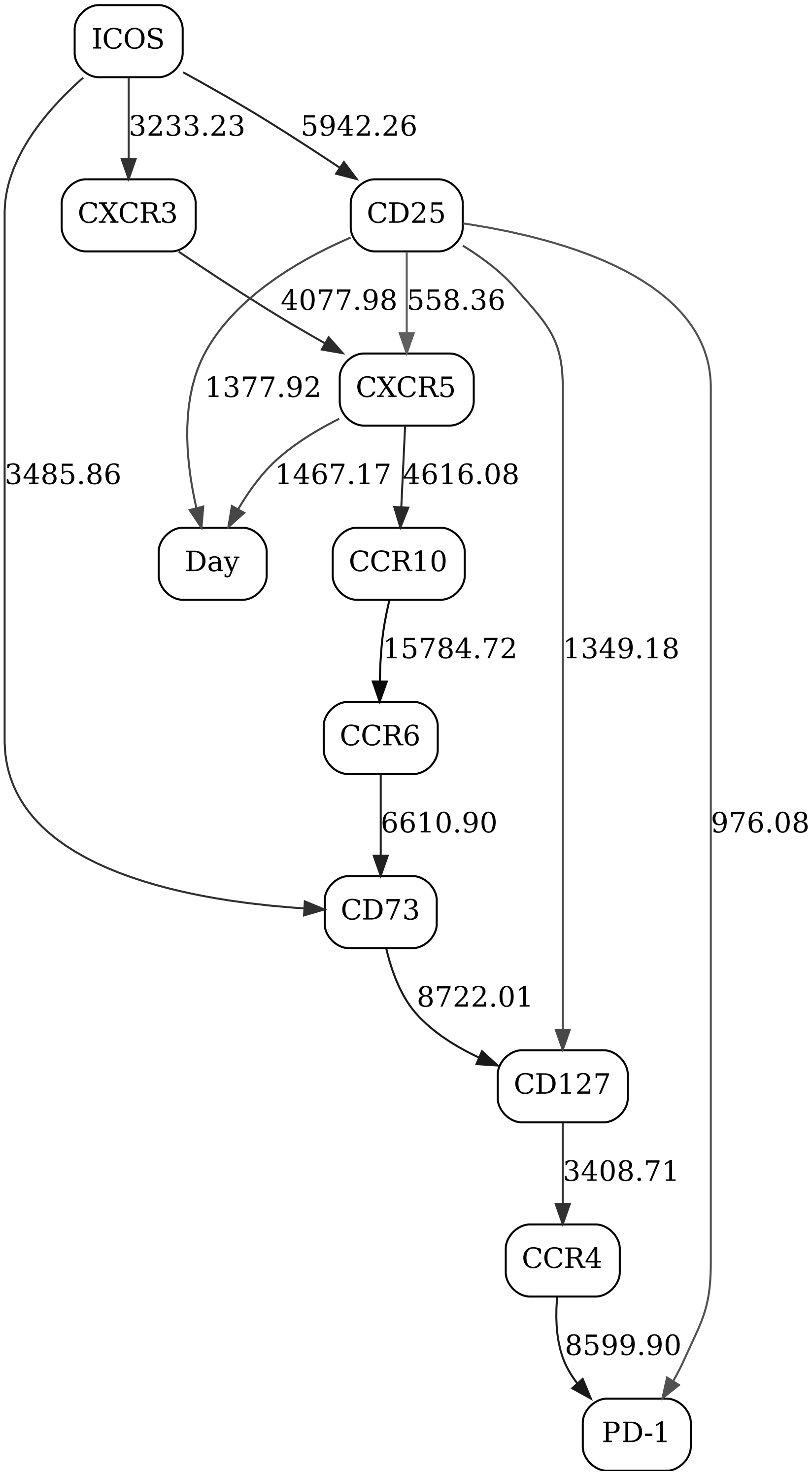

Figure 59: Adaptive immune signaling network panel, naive CD8, day contrast (2-state "Day" variable), responders

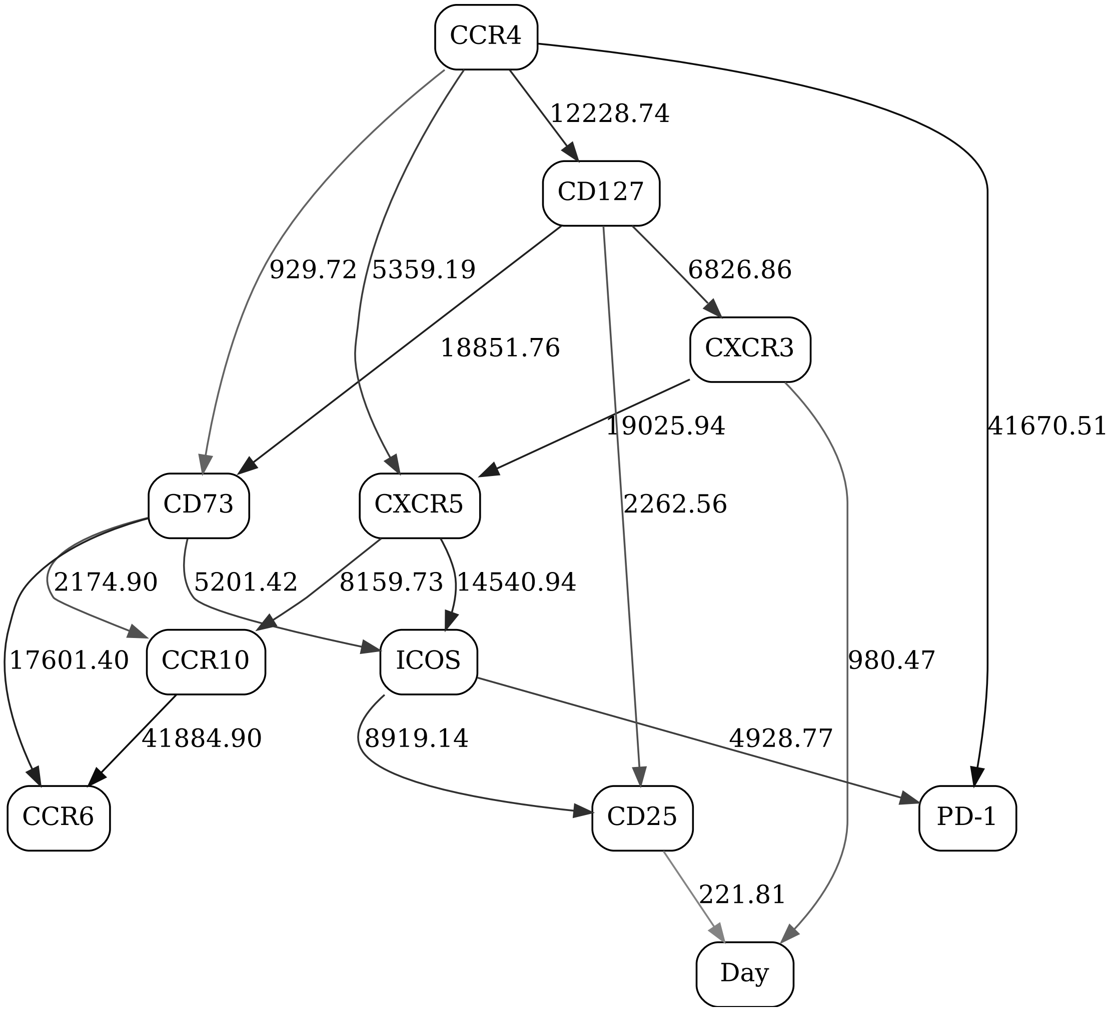

Figure 60: Adaptive immune signaling network panel, naive CD8, day contrast (2-state "Day" variable) non-responders

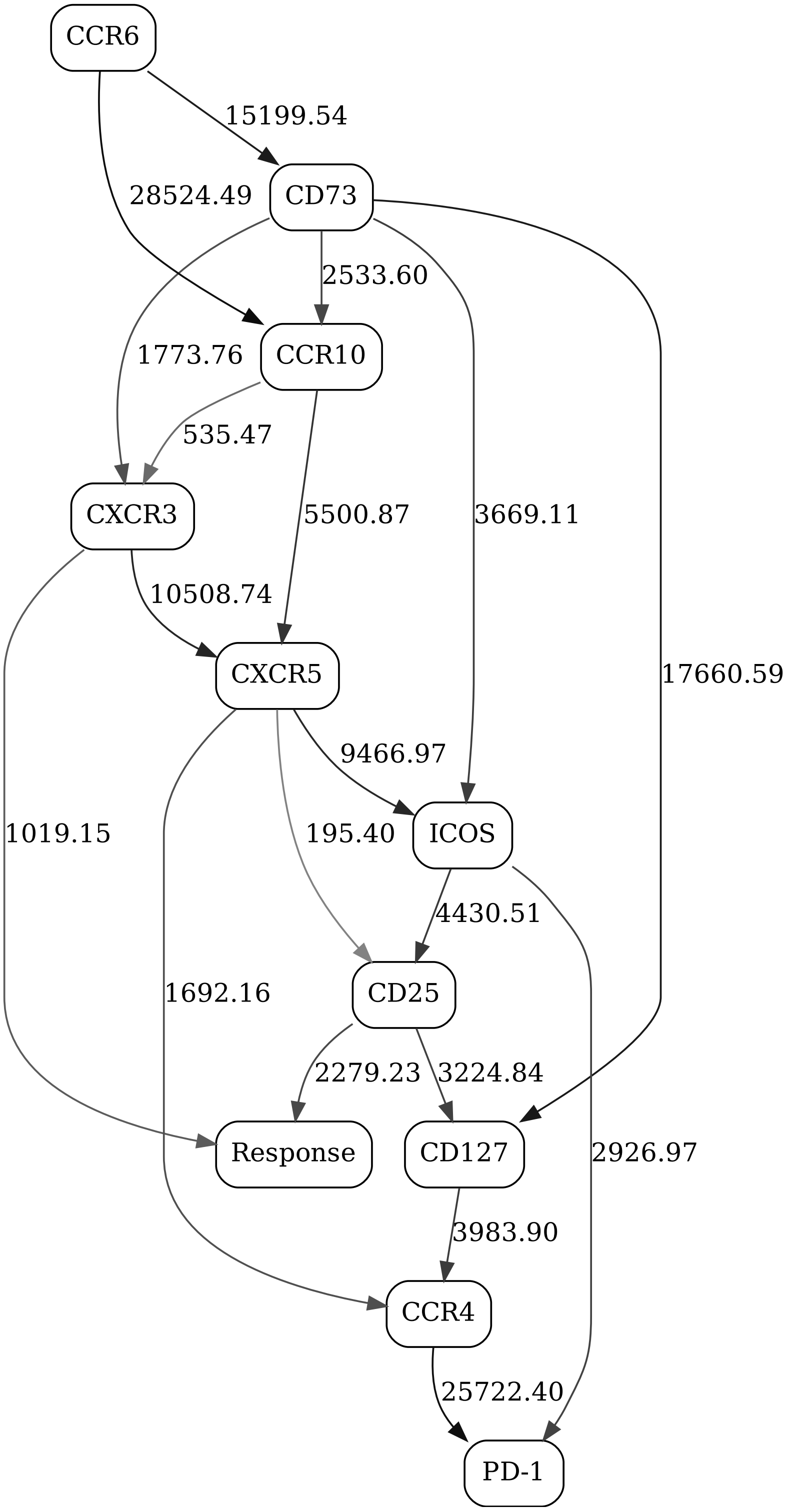

Figure 61: Adaptive immune signaling network panel, naive CD8, day 1, response contrast (2-state "Response" variable)

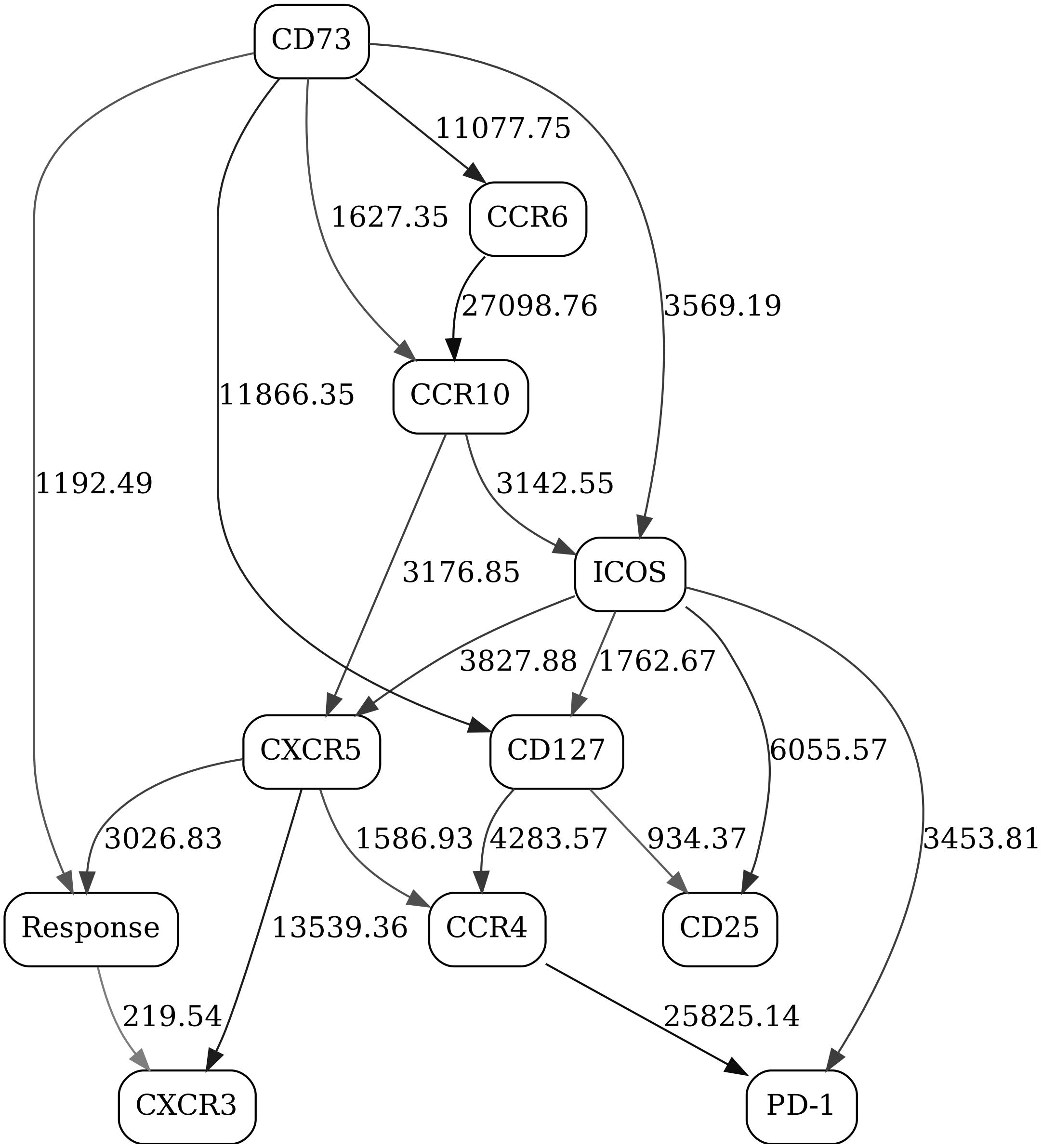

Figure 62: Adaptive immune signaling network panel, naive CD8, day 21, response contrast (2-state "Response" variable)

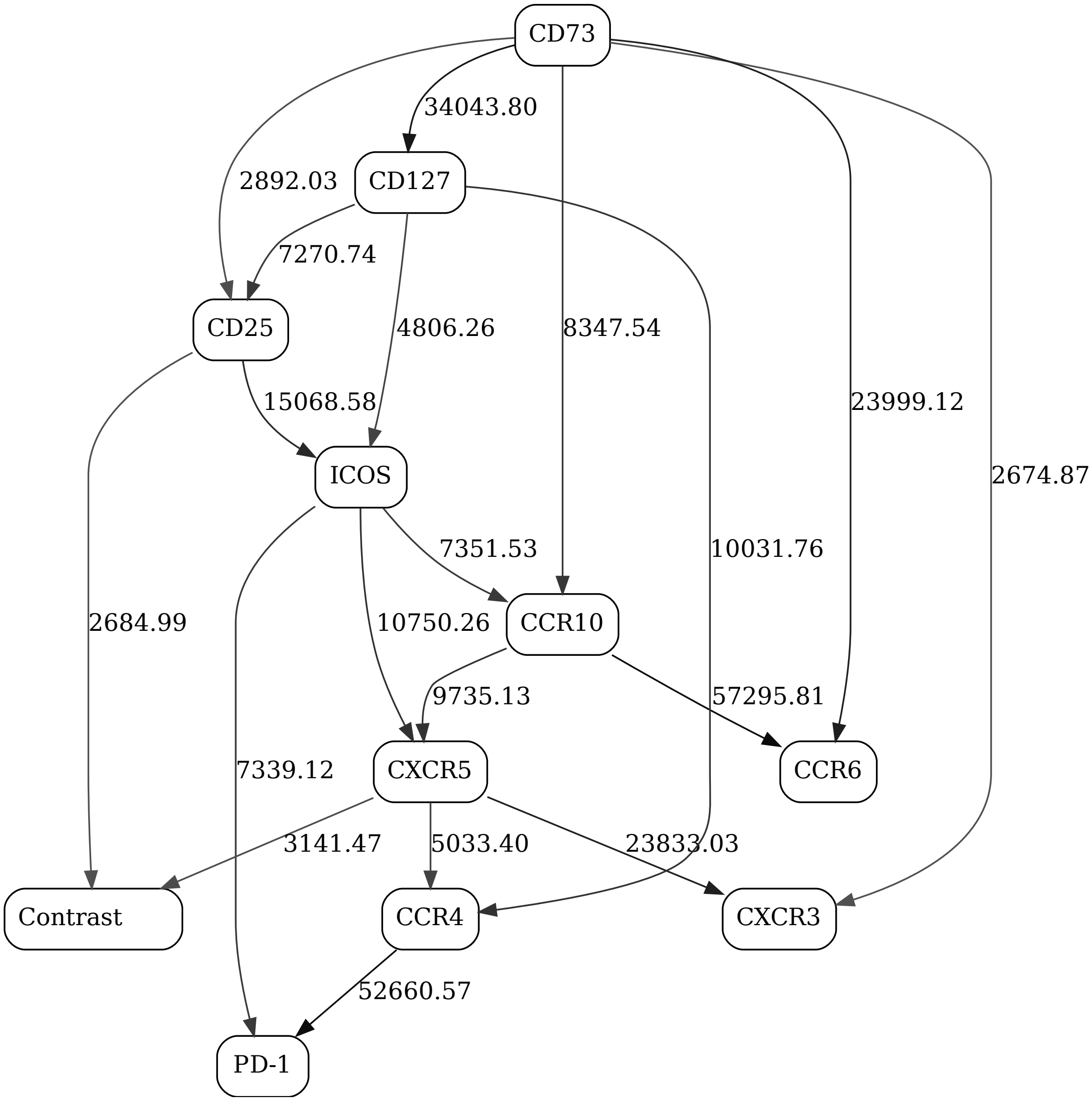

Figure 63: Adaptive immune signaling network panel, naive CD8
